# Supplementary material for: Magnon-mediated qubit coupling determined via dissipation measurements
Source: Proc Natl Acad Sci U S A. 2024 Jan 3;121(2):e2313754120. doi: 10.1073/pnas.2313754120 (PMC10786302; doi:10.1073/pnas.2313754120)
Supplement: Supplementary file 1 — Appendix 01 (PDF) [file pnas.2313754120.sapp.pdf]

Supporting Information for

Magnon-mediated qubit coupling determined via dissipation measurements

Masaya Fukami, Jonathan C. Marcks, Denis R. Candido,
Leah R. Weiss, Benjamin Soloway, Sean E. Sullivan, Nazar Delegan,
F. Joseph Heremans, Michael E. Flatté, and David D. Awschalom*
*Corresponding author. Email: awsch@uchicago.edu.

SI Experimental Setup

SI.1 Diamond slab with NV main symmetry axis parallel to the surface

In this section, we describe the preparation of the diamond slab with the NV axis (NV center's quantization axis, the $\langle 111 \rangle$ diamond crystallographic direction) parallel to the diamond sample surface. The diamond slab used in the experiments is cut out of a $2 \text{ mm} \times 2 \text{ mm} \times \ 0.5 \text{ mm}$ diamond crystal (electric grade, Sumitomo) with the top and side direction being $\langle 001 \rangle$ and $\langle 110 \rangle$, respectively (See Fig. S1). The diamond slab is laser cut and the top surface is subsequently chemically-mechanically polished (CMP) down to $R_q \leq 0.4$ nm (SYNTEK CO. LTD.). To remove the CMP induced damage, the diamond slab goes through an inductively coupled plasma reactive-ion etching (ICP-RIE), removing around 3 μm of material, while maintaining the surface roughness quality. After the ICP-RIE process, the top side of the diamond slab is implanted with $[^{15}\text{N}]$, at 5 keV with a 7° incidence angle. This results in a SRIM simulated implantation depth of 7.7 ± 3.0 nm. The implantation dose of 10^{13} ions/cm^2 results in ensemble NV densities, with ≈ 2000 NV centers observable in a 1-μm-diameter focused laser spot. After the nitrogen implantation, the sample goes through tri-acid cleaning to remove any residual graphitic species and contaminants, followed with a two-step high-temperature anneal [850 °C for 6 hours followed by 1100 °C for 2 hours in argon forming gas (95% argon, 5% H_2)] to activate the NV centers and heal the implantation induced damage, respectively. Finally, the sample istri-acid cleaned again to remove the annealing-induced graphitized layer (*1*).

We characterize the dephasing and the decoherence times of this ensemble of NV centers measured at NV locations with no YIG coverage as shown in Fig. S3A, using Ramsey and Hahn-echo spectroscopy. To this end, we firstly perform a pulsed ODMR (optically detected magnetic resonance) measurement to detect the NV-center frequency for the transition $|m_s = 0\rangle \leftrightarrow |m_s = -1\rangle$ at 30 G external magnetic field as shown in Fig. S2A, which reveals two resonant dips corresponding to the nitrogen [^{15}N] nuclear spin ($I = 1/2$). Then, we set the drive microwave frequency to be at the middle of the two dips in the pulsed ODMR, and we perform the Ramsey interferometry and the Hahn-echo spectroscopy measurements as shown in Figs. S2B and C. The oscillation observed in Fig. S2B is caused by the detuning between the drive frequency and the two NV-center frequencies

dependent on the nitrogen nuclear spin sates. This is confirmed by the frequency of the oscillation, which approximately equals to the half of the frequency difference between the two dips shown in Fig. S2A (recall that our drive frequency is chosen to be at the middle of the two dips). The (Ramsey) dephasing and the (Hahn-echo) decoherence times of the ensemble of NV centers used in this work are $T_2^* = 182(5)$ ns and $T_2 = 3.53(5)$ μs, respectively. In our system, nitrogen concentration is estimated to be 10^{13} ions/cm^2/(2 $\times$ 3 nm) $\approx 1.7 \times 10^{19}$ cm^{-3} $\approx$ 100 ppm, where we used 1 ppm $= 1.76 \times 10^{17}$ cm^{-3} for diamond. Here, the thickness of the nitrogen-doped layer is assumed to be $2 \times (3.0\ \mathrm{nm})$ as the SRIM simulation shows that the NV depth to be $\approx 7.7 \pm 3.0\ \mathrm{nm}$. Our Ramsey and Hahn-echo decay times are well explained by Ref. (*2*) with the above nitrogen concentration, indicating that the dephasing and the decoherence times are dominated by the nearby P1 centers.

SI.2 Calibration of the distance between the diamond slab and the YIG top surface

The calibration of the diamond-YIG distance is performed using the optical interference fringes as shown in Fig. S3. Figs. S3A and S3B shows the optical microscope images of the diamond plate placed on top of the YIG under a white light and a green monochromatic light, respectively. The horizontal rectangle in the image is the YIG/GGG chip and the vertical rectangle is the diamond plate placed on top of the YIG. On the right-hand side of the diamond, PMMA is used as a glue to attach the diamond onto the YIG. The image shown in Fig. S3B is taken after placing the YIG/diamond sample on an aluminium sample mount (with a thermometer) and a copper wire (AWG 30) is placed over the sample. Clear vertical optical interference fringes are visible. Then, we perform an optical reflectivity measurement with 532-nm and 636-nm lasers near the white-squared positions in Fig. S3B. The top images of Fig. S3C and D are the two-dimensional measurement of the reflected laser light, which are performed by replacing the laser-line filters in front of the APD (avalanche photodiode) with OD (optical density) filters. By summing over the intensity of the image in the vertical direction, we obtain the reflection light intensity as a function of the horizontal position, as shown in the bottom figures of Figs. S3C and D.

The horizontal positions for the destructive interference are different between Figs. S3C and S3D.

This is because the destructive interference occurs for

$$h_{\rm diamond-YIG} = \frac{\lambda}{2}n, \quad n = 0, 1, 2, \cdots \tag{S1}$$

where $h_{\rm diamond-YIG}$ is the diamond-YIG distance and λ is the laser light wavelength, i.e. $\lambda = 532$ nm and $\lambda = 636$ nm. We obtain Fig. S3E assuming that the leftmost destructive interference is the $n = n_{\rm lm} = 1$ case of Eq. (S1), as the $n = 0$ interference is not clearly visible due to the boundary of the diamond. The linear fit is shown with a black line, where the slope is $3.73(4)$ nm/μm. This assumption of $n_{\rm lm} = 1$ is validated by computing the linear fit residuals for different leftmost destructive interference label ($n_{{\rm lm},\lambda} = 1, 2, 3, 4, 5$ for $\lambda = 532$ nm and $\lambda = 636$ nm) and minimizing it. The constant slope of the diamond-YIG distance appears to be caused by a wedge between the diamond and the YIG. The wedge appears to be on the edge of the diamond.

For finding the NV center with a desired NV-YIG distance, such as $h_{\rm NV} = 400$ nm, we firstly take a horizontal FSM (fast steering mirror) scan of the PL [see Fig. S9A] and determine the left and right dip positions $X = X_{\rm left}$ and $X = X_{\rm right}$, where X is the horizontal position of the FSM. The corresponding diamond-YIG distances are calculated as $h^{\rm left}_{\rm diamond-YIG} = \lambda_{\rm green} n_{\rm left}/2$ and $h^{\rm right}_{\rm diamond-YIG} = \lambda_{\rm green}(n_{\rm left} + 1)/2$, where $\lambda_{\rm green} = 532$ nm is the wavelength of the NV initialization/readout laser. Here, we assumed that the dips in the PL scan shown in Fig. S9A correspond to the nodes in the reflection image with green (532 nm) illumination as shown in Fig. S3C. This is verified as the horizontal separation ≈ 70 μm of the two dips shown in Fig. S9A is well explained by the slope of the fit shown in Fig. S3B via $(\lambda_{\rm green}/2)/(\rm slope) \approx 70$ μm, where $(\rm slope) = 3.73(4)$ nm/μm is the slope shown in Fig. S3E. In the FSM image shown in Fig. S9A, the node label of the left dip is $n_{\rm left} = 1$ based on the analysis performed in Fig. S3E. To find the NV centers with $h_{\rm NV} = 400$ nm, we set the FSM horizontal position X to be such that $h^{\rm desired}_{\rm diamond-YIG} = h_{\rm NV} - 7.7$ nm, where 7.7 nm is the average NV depth of the NV centers from the diamond surface calculated from the SRIM simulation (see Sec. SI.1). This FSM horizontal position X is determined by finding the solution of

$$h^{\rm desired}_{\rm diamond-YIG} = \frac{(X - X_{\rm left})h^{\rm right}_{\rm diamond-YIG} + (X_{\rm right} - X)h^{\rm left}_{\rm diamond-YIG}}{X_{\rm right} - X_{\rm left}}. \tag{S2}$$

The actual variation of the NV-YIG distance $h_{\rm NV}$ within the ensemble of NV centers in the laser-

focused spot is determined by the following. Firstly, using the focused laser spot size of $\approx 1\ \mu\mathrm{m}$ and the slope $\approx\ 4\ \mathrm{nm}/\mu\mathrm{m}$ obtained from Fig. S3E, the uncertainty originating from the diamond-YIG distance calibration is $\approx (1\ \mu\mathrm{m}) \times (4\ \mathrm{nm}/\mu\mathrm{m}) = 4\ \mathrm{nm}$. Next, we recall that the ensemble NV centers are distributed with deviation $\pm 3\ \mathrm{nm}$ based on the SRIM simulation (see Sec. SI.1). Combining these two sources of the uncertainties, we obtain $\approx \sqrt{(4\ \mathrm{nm})^2 + (3\ \mathrm{nm})^2} = 5\ \mathrm{nm}$ of the uncertainty of the NV-YIG distance h_{NV}.

SI.3 Calibration and optimization of 532 nm laser power for initialization and readout

In the experiment, we set the green (532 nm) laser power for the initialization and readout of the NV centers to be $P_{\mathrm{L}} = 40\ \mu\mathrm{W}$. The initialization rate of the NV centers at this laser illumination power is characterized by a time-resolved continuous-wave ODMR measurement as shown in Fig. S4. In the measurement, the $P_{\mathrm{L}} = 40\ \mu\mathrm{W}$ green (532 nm) laser is illuminated continuously and a microwave with the NV frequency for the transition $|m_s = 0\rangle \leftrightarrow |m_s = -1\rangle$ is applied for $2\ \mu\mathrm{s}$ duration with a repetition time of $36\ \mu\mathrm{s}$. Readout time bin is set to $0.1\ \mu\mathrm{s}$ and the readout position in time is varied after the microwave is applied to observe the disappearance of the ODMR contrast due to the NV initialization. We use this simplified measurement as it does not require a calibration of a π-pulse and can be performed immediately after the continuous-wave ODMR measurement; rather, the initialization and readout times ($20\ \mu\mathrm{s}$ and $5\ \mu\mathrm{s}$, respectively) in the Rabi-oscillation measurements (needed later for the T_1 measurement) is set based on this simplified initialization time measurement. This measurement shows that the NV initialization time (to the level of $1/e$, see Fig. S4) is $6.6(3)\ \mu\mathrm{s} \approx 5\ \mu\mathrm{s}$, or the initialization rate $\approx 0.2\ \mu\mathrm{s}^{-1}$. This initialization rate sets the upper bound of the efficient measurement of the longitudinal relaxation rate, as the initialization is inefficient when the longitudinal spin relaxation is faster than the initialization rate. This power is selected to eliminate the heating effect of the YIG as detailed below.

In Fig. S5A, we show the ODMR of an ensemble of NV centers on the YIG at an external magnetic field $\mu_0 H_{\parallel} = 30$ G, for the transitions $|m_s = 0\rangle \leftrightarrow |m_s = \pm 1\rangle$ with laser powers $P_{\mathrm{L}} = 20\ \mu\mathrm{W}$ and $P_{\mathrm{L}} = 400\ \mu\mathrm{W}$. Clear shifts of the center frequency of the resonance is visible for different laser

powers. In Fig. S4B, we show the laser power dependence of the center frequency of the ODMR for the transitions $|m_s = 0\rangle \leftrightarrow |m_s = \pm 1\rangle$. Interestingly, the shift of the resonance frequencies for the two transitions are opposite, which cannot be explained by the laser heating of the diamond sample that changes the zero-field splitting $D_{\rm NV}$, which shifts both of the transitions to the lower-frequency side by the temperature sensitivity $dD_{\rm NV}/dT \approx -2\pi \times 0.1$ MHz/K.

The shift of the resonance frequencies is attributed to the heating induced change of magnetization (*3*) and the associated change of the fringe magnetic field at the NV position. YIG magnetization $M_{\rm s}$ changes under a temperature change by approximately -0.23 %/K around room temperature, corresponding to $\mu_0 dM_{\rm s}/dT \approx 3.9$ G/K, where T is the temperature and we used $M_{\rm s} = 1716$ G$/\mu_0$ (see Sec. SI.7). The magnetic-field sensitivity of the NV-center resonant frequency is governed by $\gamma = 2\pi \times 2.8$ MHz/G, where γ is the absolute-value of the gyromagnetic ratio. Assuming that the local magnetization change $\delta M_{\rm s}$ produces the static field at the NV-center position by $\delta H_{\rm stat} = \lambda \delta M_{\rm s}$, where λ is a dimensionless parameter of order one, The temperature change is estimated to affect the NV transition frequency via the magnetization change by $df/dT = \pm\gamma\mu_0 dH_{\rm stat}/dT = \pm\gamma\lambda\mu_0 dM_{\rm s}/dT = \pm 11\lambda$ MHz/K. In contrast, the laser-power dependent shift of the NV-center resonant frequencies shown in Fig. S5B have slopes $df/dP_{\rm L} = 6.5(4)$ MHz/mW and $df/dP_{\rm L} = -4.8(2)$ MHz/mW for the upper ($|m_s = 0\rangle \leftrightarrow |m_s = +1\rangle$) and the lower ($|m_s = 0\rangle \leftrightarrow |m_s = -1\rangle$) transitions, respectively. Approximating these frequency responses as $df/dP_{\rm L} \approx \pm 5.7$ MHz/mW, we obtain the temperature response to the laser power $dT/dP_{\rm L} = (5.7\ \text{MHz/mW})/(11\lambda\ \text{MHz/K}) \approx 0.52\lambda^{-1}$ K/mW.

Here we estimate the dimensionless parameter λ to be $\lambda = 1/6$. We consider a simple situation where the laser heating will make the static magnetization to be

$$\mathbf{M}_0(\mathbf{r}) = M_{\rm s}(\mathbf{r})\hat{z}, \tag{S3}$$

$$M_{\rm s}(\mathbf{r}) = (M_{\rm s} - |\delta M_{\rm s}|\Theta(r_0 - r))\Theta(-x)\Theta(x + d), \tag{S4}$$

where the YIG film extends in y-z plane, x is the YIG film thickness direction, $r = \sqrt{x^2 + y^2 + z^2}$, $d = 3$ μm is the YIG thickness, $\Theta(\cdots)$ is the Heaviside step function, $M_{\rm s}$ is the YIG saturation magnetization, and $2r_0 \approx 1$ μm is on the order of the laser focus spot, as the laser will heat the YIG

and reduce the magnetization. We assume the NV position to be right above the YIG film at the center of the laser spot $(x, y, z) = (+0^+, 0, 0)$, where $x = +0^+$ indicates that the NV center is above the YIG film, not inside or on the boundary of the YIG film. Based on the divergence-free condition of the magnetic flux density [i.e. $\nabla \cdot (\delta \mathbf{H}(\mathbf{r}) + \delta \mathbf{M}_0(\mathbf{r})) = 0$ with $\delta \mathbf{M}_0(\mathbf{r}) \equiv \mathbf{M}_0(\mathbf{r}) - M_\mathrm{s}\Theta(-x)\Theta(x+d)\hat{z}$ noting that the magnetic field is zero when $\delta M_\mathrm{s} = 0$], the magnetic field $\delta \mathbf{H}$ generated by this magnetization is described by

$$\nabla \cdot \delta \mathbf{H}(\mathbf{r}) \;=\; -\nabla \cdot \delta \mathbf{M}_0(\mathbf{r}) = -|\delta M_\mathrm{s}| \cos\theta \delta(r - r_0)\Theta(-x), \tag{S5}$$

where $\cos\theta = z/r$ and we used $r_0 < d$ to eliminate $\Theta(x + d)$. This indicates that there are effective positive magnetic charges on $z < 0$ (west half of southern hemisphere) and negative magnetic charges on $z > 0$ (west half of northern hemisphere), where we note that the YIG is on the western hemisphere and the NV is on the eastern hemisphere. As the NV center is assumed to be located at the origin, $r = 0$, the NV center experiences an extra magnetic field generated by the magnetization in $+\hat{z}$ direction. The magnetic field is

$$\delta \mathbf{H}(r = 0) = \int d\mathbf{r} \frac{|\delta M_\mathrm{s}| \cos\theta \delta(r - r_0)\Theta(-x)}{4\pi r^3} \mathbf{r} = \frac{1}{6} |\delta M_\mathrm{s}| \hat{z}. \tag{S6}$$

This indicates the dimensionless parameter λ is $\lambda = |\delta \mathbf{H}(r = 0)|/|\delta M_\mathrm{s}| = 1/6$. The $1/6$ factor makes sense as the demagnetization factor of a full sphere is known to be $1/3$. Currently we have an effective half sphere western hemisphere), so we obtain $(1/2) \times 1/3 = 1/6$. For this parameter $\lambda = 1/6$, the estimated temperature response to the laser power is $dT/dP_\mathrm{L} = 0.52\lambda^{-1}$ K/mW ≈ 3.1 K/mW.

The above estimation of the laser-induced heating $dT/dP_\mathrm{L} \approx 3.1$ K/mW is consistent with the value reported in Ref. (*3*), where a red (660 nm) laser is focused to a 1.2 μm spot on a bulk YIG sample (4 mm $\times$ 2 mm $\times$ 0.4 mm). The reported value of the equivalent stage temperature rise in response to the laser power in the work is $dT/dP_\mathrm{L} \approx 2.2(3)$ K/mW. Although this is for the red laser and the absorption coefficient is higher for the 532-nm light (*4*), the values are close enough to serve as a validation of the above arguments.

SI.4 ODMR and pulse sequences

In Fig. S6A, we show the ODMR scan with and without the YIG under the diamond plate. The continuous-wave 532-nm laser and the pulsed microwave and APD readout are preformed based on Fig. S6B. The readout time of the APD is set to $5\ \mu\mathrm{s}$, as it is approximately the initialization time (see Sec. SI.3), and the microwave is applied at the maximum available power $5\ \mathrm{W}$ for $150\ \mathrm{ns}$ duration. The pulse duration of the microwave is chosen such that it does not significantly generate parametric magnons, which becomes prominent under a similar microwave configuration after $\approx 500\ \mathrm{ns}$ based on Ref. (*5*). Multiple detailed features of the PL reduction is labeled following Ref. (*6, 7*).

Longitudinal (T_1) relaxation time measurement is performed by taking a PL contrast measurement with and without applying a π-pulse at the end of elapsed time t. We can formulate the PL measurment as

$$\langle \mathrm{PL} \rangle = A\langle \mathbb{P}_{|m_s=0\rangle} \rangle + (A - \Delta A)\langle \mathbb{P}_{|m_s=\pm 1\rangle} \rangle, \tag{S7}$$

$$\hat{\mathrm{PL}} \equiv A\mathbb{P}_{|m_s=0\rangle} + (A - \Delta A)\mathbb{P}_{|m_s=\pm 1\rangle}, \tag{S8}$$

where $\mathbb{P}_{|\psi\rangle}$ is an projection operator onto a state $|\psi\rangle$, A is the PL counts when the state is $|m_s = 0\rangle$, and ΔA is the PL contrast. When subtracting the two cases with and without applying the π-pulse, we obtain

$$\begin{aligned} \Delta\mathrm{PL}(t) &\equiv \mathrm{Tr}[\hat{\mathrm{PL}}\hat{\pi}\rho(t)\hat{\pi}^\dagger] - \mathrm{Tr}[\hat{\mathrm{PL}}\rho(t)], \\ &= \mathrm{Tr}[\hat{\pi}^\dagger\hat{\mathrm{PL}}\hat{\pi}\rho(t)] - \mathrm{Tr}[\hat{\mathrm{PL}}\rho(t)] = -\Delta A\mathrm{Tr}[\sigma_z\rho(t)] = -\Delta A\langle \sigma_z(t) \rangle, \end{aligned} \tag{S9}$$

where $\Delta\mathrm{PL}$ is the differential PL, $\hat{\pi} = -i\sigma_x$ is the π-pulse operator, $\sigma_x = |m_s = -1\rangle\langle m_s = 0| + \mathrm{H.c.}$ and $\sigma_z = \mathbb{P}_{|m_s=-1\rangle} - \mathbb{P}_{|m_s=0\rangle}$ are the Pauli operators in the subspace $\{|m_s = 0\rangle, |m_s = -1\rangle\}$, and we used

$$\hat{\pi}^\dagger\hat{\mathrm{PL}}\hat{\pi} = A\mathbb{P}_{|m_s=-1\rangle} + (A - \Delta A)\mathbb{P}_{|m_s=0,+1\rangle}. \tag{S10}$$

To obtain the longitudinal relaxation time T_1, we fit the differential PL signal with $\Delta\mathrm{PL}(t) \propto \exp(-t/T_1)$.

The calibration of the π-pulse is performed at each field conditions. When the π-pulse appears longer than 220 ns even at the maximum microwave power 5 W, we used an approximate π-pulse with 5 W microwave power and 220 ns duration, which does not affect the T_1 relaxation time measurement except for the PL contrast. This is because the off-diagonal elements (i.e. coherence) of the density matrix can be regarded as zero at the end of the elapsed time of the longitudinal relaxometry measurements. This is supported by the much shorter coherence time (see Sec. SI.1) as compared to the longitudinal relaxation time T_1, or it is because the relaxation dynamics does not automatically make a superposition of the states between $|g\rangle = |m_s = 0\rangle$ and $|e\rangle = |m_s = -1\rangle$ after the initialization to the state $|g\rangle$ under a free evolution. We note that the π-pulse is applied at the end of the elapsed time in the longitudinal relaxometry measurement, not the beginning of the elapsed time as performed in some other works. We choose this sequence to avoid possible excitation of magnons by the microwave pulse at the beginning of the elapsed time and affecting the NV evolution during the elapsed time. For this pulse sequence, the insensitivity of the T_1 measurment to the π-pulse calibration is shown as follows. Under the diagonal assumption of the density matrix after the elapsed time, we can write the density matrix to be $\rho(t) = a\mathbb{P}_{|m_s=0\rangle} + b\mathbb{P}_{|m_s=-1\rangle} + c\mathbb{P}_{|m_s=+1\rangle}$ for real numbers (a, b, c) under the constraint $a + b + c = 1$. On the other hand, the imperfect π-pulse operator can be written as $\hat{\pi}_{\text{imp}} = \exp(-i\sigma_x\theta/2) = \cos(\theta/2) - i\sigma_x \sin(\theta/2)$ with $\theta = \Omega t$ for the Rabi rate Ω and the imperfect pulse time $t \neq \pi/\Omega$. Then, the PL signal after this imperfect π-pulse is written by

$$\text{Tr}[\hat{\text{PL}}\hat{\pi}_{\text{imp}}\rho(t)\hat{\pi}^{\dagger}_{\text{imp}}] = \cos^2(\theta/2)\text{Tr}[\hat{\text{PL}}\rho(t)] + \sin^2(\theta/2)\text{Tr}[\hat{\text{PL}}\hat{\pi}\rho(t)\hat{\pi}^{\dagger}], \tag{S11}$$

where we used the condition that $\rho(t)$ is diagonal to eliminate the terms proportional to $\cos(\theta/2)\sin(\theta/2)$. As the differential PL signal [see Eq. (S9)] is obtained by subtracting Eq. (S11) with $\text{Tr}[\hat{\text{PL}}\rho(t)] = \cos^2(\theta/2)\text{Tr}[\hat{\text{PL}}\rho(t)] + \sin^2(\theta/2)\text{Tr}[\hat{\text{PL}}\rho(t)]$, when we apply the imperfect π-pulse, we obtain the differential PL signal as

$$\Delta\text{PL}(t) = -\sin^2(\theta/2)\Delta A\langle\sigma_z(t)\rangle. \tag{S12}$$

This confirms that the imperfect π-pulse [$\theta = \pi + (\text{error})$] only reduces the differential PL signal by a constant factor $\sin^2(\theta/2)$, and does not affect the T_1 measurement when we fit the differential PL

signal by an exponential function $\Delta\mathrm{PL}(t) \propto \exp(-t/T_1)$.

The above argument is not specific for our choice of the timing of applying the π-pulse, where the π-pulse is applied at the end of the elapsed time and not the beginning. Even when the π-pulse is applied at the beggining of the elapsed time, the imperfect π-pulse does not affect the T_1 measurement except for the PL contrast. This is again supported by the shorter coherence time T_2^* as compared with the timescale of T_1 measurements. Starting from the ground state $|g\rangle$, an imperfect π-pulse initially generates a state $\sqrt{\alpha}|g\rangle + \sqrt{1-\alpha}|e\rangle$ with $\alpha \ll 1$. The initial density matrix is

$$\rho_{\mathrm{init}} = [|e\rangle \;\; |g\rangle] \begin{bmatrix} 1-\alpha & \sqrt{\alpha(1-\alpha)} \\ \sqrt{\alpha(1-\alpha)} & \alpha \end{bmatrix} \begin{bmatrix} \langle e| \\ \langle g| \end{bmatrix}. \tag{S13}$$

After an evolution time t many multiples of T_2^*=185 ns, the coherence (off-diagonal terms) die away and the density matrix (ignoring T_1 decay for the time-being) is

$$\rho(t) = [|e\rangle \;\; |g\rangle] \begin{bmatrix} 1-\alpha & 0 \\ 0 & \alpha \end{bmatrix} \begin{bmatrix} \langle e| \\ \langle g| \end{bmatrix}. \tag{S14}$$

Taking this as the starting point of a T_1 measurement, we see the total contrast is $(1-\alpha)-\alpha = 1-2\alpha$. Thus this still performs a T_1 measurement, with reduced contrast.

SI.5 Microwave reflection measurement of the FMR and M_s characterization

To characterize the saturation magnetization M_s of the YIG sample, we performed the microwave reflection (S_{11}) measurement with a vector network analyzer using the same copper wire placed above the YIG sample [see Fig. S3B]. The top panel in Fig. S7 shows the magnetic field dependence of the change of $|S_{11}|$ near the FMR at multiple microwave frequencies. The microwave power used in the measurement is minimized to $10\ \mathrm{nW}$ in order to suppress nonlinear phenomena of magnons (*5, 8, 9*). We regard the field providing the minimum of $\Delta|S_{11}|$ for each microwave frequency f_{MW} as the FMR resonance field $\mu_0 H_{\mathrm{FMR}}(f_{\mathrm{MW}})$, and plot $(\mu_0 H_{\mathrm{FMR}}(f_{\mathrm{MW}}), f_{\mathrm{MW}})$ in the lower panel of Fig. S7 with red circles on top of the heat map of the ODMR shown in Fig. S6A.

We fit the red circles in the lower panel of Fig. S7 using the Kittel equation

$$f_{\mathrm{FMR}}(H) = \gamma\mu_0\sqrt{H(H+M_s)}, \tag{S15}$$

where H is the external magnetic field and f_{FMR} is the FMR frequency. We use the saturation magnetization M_{s} as a fitting parameter, and the best fit is obtain by $\mu_0 M_{\mathrm{s}} = 1814 \pm 11$ G. This value is approximately 6% larger than what we obtain from the fit of $\Delta(1/T_1)$ [$M_{\mathrm{s}} = 1716(1)$ G, see Sec. SI.7]. This would be due to the error in the identification of the FMR field as the field providing the largest change in $\Delta(1/T_1)$. In our measurement, we did not observe a single dip in $\Delta|S_{11}|$, which would be due to the complex transmission property of the copper wire and the other parts of printed circuits board, in addition to the magnon-originated radiation resistance under the complex effects of the surface anisotropy of YIG. While the radiation resistance per unit length of the copper-wire part is calculated to be the largest at the FMR field and monotonically decrease as the field is away from the FMR based on the calculation without the YIG surface anisotropy effects in Sec. SII.4, we did not observe a simple single peak. Additionally, the existence of the YIG edge may add further complexity for the interpretation of $\Delta|S_{11}|$ data.

SI.6 Longitudinal relaxometry measurement

In Figs. S8A and B, we show the elapsed time t dependence of the differential PL signal and their fits for obtaining the T_1 relaxation time at multiple external magnetic fields, which are used for obtaining Fig. 2B. Fig. S8A shows $\Delta\mathrm{PL}(t)$ for $H_\parallel \leq 82$ G while Fig. S8B shows $\Delta\mathrm{PL}(t)$ for $H_\parallel \geq 82$ G. The scanning time range is selected for each field by performing a logarithmic-scale T_1 measurements first. As a reference, we show in Fig. S8C a different view of Fig. 3A, where the horizontal axis is non-uniform for each for sections.

To show the robustness of the T_1 measurements against the NV position in the diamond sample, we perform a simplified two-point T_1 measurement at multiple spatial position in the sample. In the measurement, we only measure the differential PL signal at two elapsed times $t = 0$ and $t = t_{\mathrm{meas}} = 10\ \mu$s. The resonance frequency and the π-pulse of the NV centers are determined at each spatial positions. The longitudinal relaxation time T_1 is quantified by

$$T_1 = t_{\mathrm{meas}} \ln[\Delta\mathrm{PL}(0)/\Delta\mathrm{PL}(t_{\mathrm{meas}})]. \tag{S16}$$

We show the spatial map of T_1 in Fig. S9B at the external magnetic field $\mu_0 H_\parallel = 82$ G. The wider

PL image is shown in Fig. S9A as a reference, where the scanning range of Fig. S9B is marked with a squared part in Fig. S9A. Fig. S9A shows that the NV-YIG distance h_{NV} is approximately the same across the vertical direction. Based on the analysis in Sec. SI.2, the vertical destructive optical interference fringes on the left and right in Fig. S9A correspond to $h_{\mathrm{diamond-YIG}} = (532/2)$ nm and $h_{\mathrm{diamond-YIG}} = 532$ nm, respectively. In Fig. S9C, we visualize the spatial dependence of the NV-center resonance frequency that is used for the π-pulse. Here, $\Delta f_{\mathrm{d}} = f_{\mathrm{dip}} - (D_{\mathrm{NV}} - \gamma\mu_0 H)/2\pi$ is the resonance frequency deviation from the calculated frequency, where f_{dip} is the NV-center frequency determined from the continuous-wave ODMR and D_{NV} is the zero-field splitting of the NV center. This shows there is a small external field magnetic field variation $\approx (2\pi \times 3\ \mathrm{MHz})/\gamma \lesssim 1$ G. Even though there is a slight external field deviation, Fig. S9B clearly shows that the T_1 relaxation rate is large on the left and small on the right, consistent with the NV-YIG distance h_{NV}.

As a reference, we also perform similar measurements of the spatial map of $1/T_1$ as in Fig. S9B on the upper edge of the YIG sample [see Fig. S3A]. Fig. S10A shows the spatial scan of the PL of an ensemble of NV centers scanned in the same way as in Fig. S9A. The horizontal wiggly curve in the middle shows the boundary of the YIG sample. The vertical destructive optical interference fringes on the left and right within the lower half of the image correspond to $h_{\mathrm{diamond-YIG}} = (532/2)$ nm and $h_{\mathrm{diamond-YIG}} = 532$ nm, respectively, based on the analysis in Sec. SI.2. Inside the squared part, we scan the longitudinal relaxation rate $1/T_1$ using the simplified measurement with Eq. (S16) in the same way as in Fig. S9B. Notably, the longitudinal relaxation rate $1/T_1$ drops (i.e. T_1 increases) when we scan the NV centers without the YIG coverage on the upper side of the image. While it may be interesting to explore the effect of edge magnon modes from this type of measurements, we find that the resonance frequency also changes drastically as shown in Fig. S10C, where the magnetic field deviation is as large as $(2\pi \times 120\ \mathrm{MHz})/\gamma \approx 40$ G near this position. Still, this measurement confirms that the control measurement taken with NV centers off the YIG sample shown in Fig. 2B is robust against the choice of the NV position without the YIG coverage.

SI.7 Calculation of the longitudinal relaxation rate

In Fig. 2B we show the theoretical calculation of $1/T_1$ on top of our experimental observation. The saturation magnetization parameter M_s is determined by varying M_s by 1 G$/\mu_0$ step and minimizing the mean square error. The detail of the numerical calculation of the magnon-induced longitudinal relaxation rate is provided in Sec. SIII. At $M_\mathrm{s} = M_{\mathrm{s},*} = 1716$ G$/\mu_0$, where the asterisk subscript signifies the optimal condition (i.e. $M_{\mathrm{s},*}$ is the argument of the mean square error minimum), we obtain the minimum of the room mean square error as

$$(\mathrm{RMSE}) = \sqrt{\frac{\sum_{i=1}^{N_H}(Y_i^{\mathrm{exp}} - Y_i(M_{\mathrm{s},*}))^2}{N_H}} = 0.0039\ \mu\mathrm{s}^{-1}, \tag{S17}$$

where $i = 1, 2, \cdots, N_H$ labels the external magnetic fields where we measured $\Delta(1/T_1)$ in Fig. 2B, $N_H = 27$ is the number of different magnetic fields, Y_i^{exp} is the experimentally obtained $\Delta(1/T_1)$, and $Y_i(M_\mathrm{s})$ is the numerically calculated longitudinal relaxation rate with the saturation magnetization M_s. The root means square error (RMSE) quantifies the goodness of the fit in terms of the mean deviation between the theory and the experiment in the unit of relaxation rate. Following the nonlinear regression analysis, the uncertainty of this best-fit parameter is found to be $\delta M_\mathrm{s} = 0.6$ G$/\mu_0 \approx$ 1 G$/\mu_0$, i.e. our best-fit is obtained by $M_\mathrm{s} = 1716(1)$ G$/\mu_0$. This is achieved by firstly calculating the $(N \times 1)$-Jacobian matrix $\mathbf{J}$ via

$$Y_i(M_{\mathrm{s},*} + \Delta M) - Y_i(M_{\mathrm{s},*}) = J_{i1}\Delta M_\mathrm{s}. \tag{S18}$$

In our analysis, we used the mean of the case with $\Delta M_\mathrm{s} = 1$ G$/\mu_0$ and $\Delta M_\mathrm{s} = -1$ G$/\mu_0$ as a Jacobian matrix to simplify of the numerical computation. Then, the (1×1)-covariance matrix is given by

$$(\mathrm{Cov})_{11} = \left(\sum_{i=1}^{N_H} J_{i1} * J_{i1}\right)^{-1} (\mathrm{RMSE})^2 \times (N_H/(N_H - 1)), \tag{S19}$$

where the factor $(N_H/(N_H - 1))$ is due to our definition of the room mean square error in Eq. (S17). From this analysis, we obtain the error of the fit as

$$\delta M_\mathrm{s} = \sqrt{(\mathrm{Cov})_{11}} \approx 0.6\ \mathrm{G}/\mu_0 \approx 1\ \mathrm{G}/\mu_0. \tag{S20}$$

Another metric of the goodness of the agreement is the reduced chi-squared static χ^2. Specifically, we use the reduced chi-squared per degree of freedom χ^2_ν, where $\nu = N_H - 1$ is the degree of freedom. They are given by

$$\chi^2 = \sum_{i=1}^{N_H} \frac{(Y_i^{\rm exp} - Y_i(M_{{\rm s},*}))^2}{\sigma_i^2}; \;\; \chi^2_\nu = \frac{\chi^2}{N_H - 1} = 15.77, \tag{S21}$$

This shows that the discrepancy between the experiment and the theory is approximately $\sqrt{\chi^2_\nu} = 3.97$ times larger than the error bars on average. While this may indicate a poor agreement, the value $\chi^2_\nu \gg 1$ is mainly due to the small error bars of the $1/T_1$ measurement, where we averaged the measurements long enough to obtain a clear data. This enabled us to observe finer details of $1/T_1$ which the theoretical calculation did not capture. Note that the error bars of the $1/T_1$ measurement are based on the covariance matrix of the fit. They are on average $(\text{mean error bar}) = 0.00132\ \mu\text{s}^{-1}$, which is nearly $\sqrt{\chi^2_\nu} = 3.97$ times smaller than (RMSE). Despite the large χ^2_ν-value, (RMSE) is only $\approx 2.1\%$ of the maximum of $\Delta(1/T_1)$ $[(\text{max}) \approx 0.18\ \mu\text{s}^{-1}]$ and $\approx 11\%$ of the mean of $\Delta(1/T_1)$ $[(\text{mean}) \approx 0.035\ \mu\text{s}^{-1}]$. Furthermore, (RMSE) is approximately 8.1% of the standard deviation of $\Delta(1/T_1)$ $[(\text{std}) \approx 0.048\ \mu\text{s}^{-1}]$, which is related to the coefficient of determination R^2 via $8.1\% = 0.081 = \sqrt{1 - R^2}$. While it is known that the R^2-value is invalid for nonlinear regressions, we obtain $R^2 = 0.9967$ as another unitless metric of the goodness of agreement. Based on these observations, we conclude that the theoretical calculation agrees well with the experiments.

The numerical calculation of the magnon-induced longitudinal relaxation rate $1/T_1$ involves the calculation of the magnon-mode frequency $\omega_{\mathbf{k},n}$ and the NV-magnon coupling $g_{\mathbf{k},n}$ for magnons labeled by $(\mathbf{k}, n)$, where $\mathbf{k}$ is the magnon wave vector and n is the label for the thickness-direction modes (see Sec. SIII). Note that the NV-magnon coupling is proportional to the magnon-originated magnetic field at the NV position, specifically h_+. Following Sec. SIII, we show in Fig. S11 the dispersion relation of magnons and the magnon-originated magnetic fields with colors labeling the different magnons-modes n. Specifically, Figs. S11A and B show the wavenumber dependence for $\mathbf{k} \parallel \mathbf{H}$ and $\mathbf{k} \perp \mathbf{H}$, respectively, and Fig. S11C shows the magnon propagation angle $\phi_\mathbf{k}$ dependence at a fixed amplitude of the wave vector $|\mathbf{k}| = 1/2h_{\rm NV}$, where $\phi_\mathbf{k} = \frac{\mathbf{k}\cdot\mathbf{H}_{\rm ext}}{|\mathbf{k}||\mathbf{H}_{\rm ext}|}$ is the angle between the magnon wave vector and the external magnetic field. We note that $\phi_\mathbf{k} = \pi/2$ corresponds to the

surface wave geometry (see sec. SII) where the spin wave is localized on the upper surface of the YIG film (NV-center side of the YIG film), while $\phi_{\mathbf{k}} = 3\pi/2$ is the case where the spin wave is localized on the opposite side of the YIG film. The dispersion relation of magnons is highly anisotropic. Importantly, under the surface-wave geometry, the field generated by magnons is much larger, especially h_+ which is responsible for the NV-MSSW coupling $g_{\mathbf{k},n}$. In the inset of Fig. S11B, we show a predicted curve from Eq. (S144) in green, showing a good agreement between the numerically calculated results and the analytic results from Eq. (S144) based on the model where the exchange stiffness parameter D_{ex} is set to zero $D_{\text{ex}} = 0$.

In Fig.2C, we presented the dispersion relation of the MSSW mode with a pink curve and the band of other magnon modes as a gray shaded area. However, more accurately there are many thickness-direction modes that are hybridizing to each other, making the distinction between the surface and volume waves unclear. The more accurate visualization of the dispersion relation of magnon modes are shown in Fig. S12A, where the surface localization for each mode is colored with pink, showing that the higher-k modes are less localized due to the exchange interaction. Unlike the case with zero exchange stiffness $D_{\text{ex}} = 0$ in Sec. SII, the surface localization feature is destroyed at higher wavenumber when $D_{\text{ex}} \neq 0$. This surface localization is quantified by

$$(\text{localization}) = \frac{\int dx \mathcal{F}(x) e^{kx} \xi_{\mathbf{k},n}(x)}{\sqrt{\int dx \mathcal{F}(x) |e^{kx}|^2}}, \tag{S22}$$

where $\xi_{\mathbf{k},n}(x)$ is the mode function defined in Eq. (S204) and $\mathcal{F}(x)$ is one (zero) inside (outside) the YIG, which we define in Sec. SIII. Our visualization of the dispersion relation in Fig. 2C can be though of as a simplified version of Fig. S12A to highlight the plateau feature of the MSSW mode.

Furthermore, one can show that the peak in the noise spectrum shown in Fig. 2C arises from the MSSW mode (i.e. magnons propagating in the $\phi_{\mathbf{k}} = \pi/2$ direction) by limiting the integration over the angle $\phi_{\mathbf{k}}$ for the numerical calculation of $\mathcal{S}(\omega)$. More concretely, we numerically compute

$$\mathcal{S}(\omega) = \coth(\beta\omega/2) \sum_n \int \frac{d\mathbf{k}}{(2\pi)^2} |g_{\mathbf{k},n}|^2 \pi \delta(\omega_{\mathbf{k},n} - \omega), \tag{S23}$$

$$\mathcal{S}(\omega | 80^\circ \leq \phi_{\mathbf{k}} \leq 100^\circ) = \coth(\beta\omega/2) \sum_n \int_{80^\circ \leq \phi_{\mathbf{k}} \leq 100^\circ} \frac{d\mathbf{k}}{(2\pi)^2} |g_{\mathbf{k},n}|^2 \pi \delta(\omega_{\mathbf{k},n} - \omega), \tag{S24}$$

where $\mathcal{S}(\omega)$ is the full noise spectrum and $\mathcal{S}(\omega|80^\circ \leq \phi_{\mathbf{k}} \leq 100^\circ)$ is the angle-restricted noise spectrum. In Fig. S12B, we show the full noise spectrum and the angle-restricted noise spectrum. We find that approximately 81% of the peak amplitude in the full noise spectrum $\mathcal{S}(\omega)$ is coming from the magnons propagating in the range $80^\circ \leq \phi_{\mathbf{k}} \leq 100^\circ$. This further confirms that the peaked structure of $\mathcal{S}$ is cauised by the MSSW plateau.

SI.8 Scaling of $\Delta(1/T_1)$ in the inset of Fig. 3A

In Fig. S13, we show a power-law fit $\Delta(1/T_1) \propto h_{\rm NV}^{\alpha}$ for the inset of Fig. 3A. The best fit is obtained by $\alpha = -2.4(1)$. This $\alpha \approx -2$ can be explained by approximating both the coupling constant and dispersion relation as

$$g_{\mathbf{k}} = \frac{\gamma\mu_0}{\sqrt{2}}\sqrt{k\gamma M_{\rm s}}e^{-kh_{\rm NV}}\sin^2\phi_{\mathbf{k}}\mathbb{I}_{0\leq\phi_{\mathbf{k}}\leq\pi}, \tag{S25}$$

$$\omega_{\mathbf{k}} = \omega_H + (\omega_M/2)\sin^2\phi_{\mathbf{k}} + D_{\rm ex}k^2, \tag{S26}$$

where $\mathbb{I}_{0\geq\phi_{\mathbf{k}}\geq\pi} = \Theta(\phi_{\mathbf{k}})\Theta(\pi - \phi_{\mathbf{k}})$ is an indicator function, $D_{\rm ex}$ is the exchange stiffness. These approximated expressions are obtained considering both the NV-MSSW coupling and the magnon plateau frequency with $\phi_{\mathbf{k}} = \pi/2$ in Eqs. (S144) and (S145) in the $D_{\rm ex} \to 0$ and $k \gg 1/d$ limits, along with the dependence of the magnon-originated magnetic field and the magnon-mode frequency shown in Fig. S11C. More concretely, the proposed form of the coupling Eq. (S25) reduces to Eq. (S144) at $\phi_{\mathbf{k}} = \pi/2$ and becomes zero when $\phi_{\mathbf{k}} = 0, \pi$, consistent with the behavior of h_+ in Fig. S11C. We note that Eq. (S25) and Eq. (S144) are different by a factor of $\sqrt{L^2}$ even for $\phi_{\mathbf{k}} = \pi/2$. However, this is merely due to a convention on the commutation relation of the creation and annihilation operators, which is widely known in physics (*10*). For Eq. (S25) the commutation relation is a Dirac delta function, while for Eq. (S144) the commutation relation is a Kronecker delta. Similarly, the proposed form of the dispersion relation Eq. (S26) reduces to the MSSW plateau frequency Eq. (S145) for $\phi_{\mathbf{k}} = \pi/2$ when the wave number k is much smaller than the inverse of the exchange wavelength $a_{\rm ex} \equiv \sqrt{D_{\rm ex}/\omega_M} \approx 17.7$ nm, where $\omega_M = \gamma\mu_0 M_{\rm s}$ is the frequency (energy) scale characterizing the magnetic dipole-dipole interaction between the spins in the ferromagnet (or ferrimagnet) with the saturation magnetization $M_{\rm s}$, i.e. $\omega_M = \mu_0\gamma^2 \times (\text{spin density}) = \mu_0\gamma^2/(\text{spin–spin distance})^3$,

where we used $M_{\mathrm{s}} = \gamma \times (\text{spin density})$. Additionally, the proposed dispersion reaches near the magnon-mode lower bound ω_H [see Sec. SIII and Fig. S11C] for $\phi_{\mathbf{k}} = 0, \pi$.

When the NV frequency is set to the MSSW plateau frequency $\omega_{\mathrm{NV}} = \omega_H + \omega_M/2$, we obtain the longitudinal relaxation rate as (see Sec. SIII)

$$1/T_1 \;=\; \coth(\beta\omega/2) \int \frac{d\mathbf{k}}{(2\pi)^2} |g_{\mathbf{k}}|^2 2\pi\delta(\omega_{\mathbf{k}} - (\omega_H + \omega_M/2)), \tag{S27}$$

where β is the inverse temperature and $\int d\mathbf{k}$ is a two-dimensional integral. The direct evaluation of this integral using the coupling and the dispersion in Eqs. (S25) and (S26) results in the scaling behavior of the longitudinal relaxation rate $1/T_1 \propto h^{\alpha}$ with $\alpha = -2$. By integrating over k first and $\phi_{\mathbf{k}}$ next, we obtain

$$\begin{aligned} 1/T_1 &= \coth(\beta\omega_{\mathrm{NV}}/2) \frac{1}{(2\pi)^2} \int_0^{\pi} d\phi_{\mathbf{k}} \int_0^{\infty} dk k |g_{\mathbf{k}}|^2 2\pi\delta\left((\omega_M/2)\cos^2\phi_{\mathbf{k}} - D_{\mathrm{ex}}k^2\right), \\ &= \coth(\beta\omega_{\mathrm{NV}}/2) \frac{\omega_{a_{\mathrm{ex}}}}{4\sqrt{2}\pi A^2} \left[\frac{A^2}{5} - \frac{3\pi}{2}(I_3(A) - L_3(A))\right], \end{aligned} \tag{S28}$$

where $A = \sqrt{2}h_{\mathrm{NV}}/a_{\mathrm{ex}}$, $\omega_{a_{\mathrm{ex}}} = \mu_0\gamma^2/a_{\mathrm{ex}}^3$, $I_n(z) = \mathrm{BesselI}[n, z]$ is the Bessel function of the first kind, and $L_n(z) = \mathrm{StruveL}[n, z]$ is the modified Struve function. In the limit $A \gg 1$, the term inside the bracket in Eq. (S28) becomes one. Therefore, when $h_{\mathrm{NV}} \gg a_{\mathrm{ex}}$, we obtain

$$1/T_1 \approx \coth(\beta\omega_{\mathrm{NV}}/2) \frac{\omega_{a_{\mathrm{ex}}}}{8\sqrt{2}\pi} \left(\frac{a_{\mathrm{ex}}}{h_{\mathrm{NV}}}\right)^2 = \coth(\beta\omega_{\mathrm{NV}}/2) \frac{\mu_0\gamma^2}{8\sqrt{2}\pi a_{\mathrm{ex}} h_{\mathrm{NV}}^2}. \tag{S29}$$

This is shown with a green dashed curve in Fig. S13, confirming the validity of the above approximations within our experimental conditions. We note that in the opposite limit $A \ll 1$, the part inside the bracket in Eq. (S28) becomes $A^2/5$, leading to an upper bound of $1/T_1$ as

$$1/T_1 \leq \coth(\beta\omega_{\mathrm{NV}}/2) \frac{\omega_{a_{\mathrm{ex}}}}{20\sqrt{2}\pi} = \coth(\beta\omega_{\mathrm{NV}}/2) \frac{\mu_0\gamma^2}{20\sqrt{2}\pi a_{\mathrm{ex}}^3} \approx 39\ \mu\mathrm{s}^{-1}. \tag{S30}$$

This indicates the dependency $1/T_1 \propto h_{\mathrm{NV}}^{-2}$ shown in Eq. (S29) is valid for $h_{\mathrm{NV}} \geq \sqrt{5}a_{\mathrm{ex}} \approx 40$ nm. The last numerical value in Eq. (S30) is obtained using our experimental parameters at room temperature. However, the above analytical expressions are based on our guessed coupling Eq. (S25) inspired by the MSSW computation with $D_{\mathrm{ex}} = 0$, which is valid only for magnons with small wavenumber $k \ll a_{\mathrm{ex}}^{-1}$. Therefore, the validity of the above computation when h_{NV} is small is unclear, as magnons

with large wavenumbers contribute significantly. In such cases, we expect the numerical approach we present in Sec. SIII provides more accurate predictions. Still, this analytical computation supports our numerical calculations within our experimental conditions and provides an insight into what parameters determine the magnitude of $1/T_1$.

SI.9 Qualitative explanation of the dependency in Fig. 3B

Qualitative explanation of the dependency in Fig. 3B can be found as follows. Let H_c be the magnetic field providing the maximum of $\Delta(1/T_1)$ under the change of $H_\parallel$ with fixed $H_\perp$. The observed decrease of H_c under increasing $H_\perp$ in Fig. 3B can be explained by the combination of both the dependency of the NV transition frequency f_NV and the MSSW plateau frequency f_p as a function of $H_\perp$. To this end, we show the NV center frequency f_NV, measured by a continuous-wave ODMR, as a function of the external magnetic field $H_\parallel$ at multiple perpendicular fields $H_\perp$ in Fig. S14A with corresponding colors to Fig. 3B. The error bars and the semi-transparent vertical bars represent the fit error and the line width of the Lorentzian fit function, respectively, as a reference for the accuracy of the resonant frequency detection. Black circles show H_c and the corresponding f_NV. Clearly, the shift in H_c cannot be explained by a magnetic-field-independent noise source with a fixed frequency. We show the calculated f_NV as a function of $H_\parallel$ at multiple $H_\perp$ in Fig. S14B with solid lines with corresponding colors to Figs S14A. While there is a small mismatch between Figs. S14A and B due to the inaccuracy of the external magnetic field calibration, we find an agreement in their general trend. Furthermore, we plot the MSSW plateau frequency $f_\mathrm{p} = (\gamma\mu_0|\mathbf{H}| + \omega_M/2)/2\pi$ in Fig. S14B with dotted lines with corresponding colors, where $|\mathbf{H}| = (H_\parallel^2 + H_\perp^2)^{1/2}$ is the absolute amplutde of the external magnetic field. The crossing conditions between f_NV and f_p for each $H_\perp$ are marked with black circles. We note that there is a small mismatch between the analytical expression of f_p and the numerically calculated plateau frequency, defined by the frequency providing the minimum slope of the magnon dispersion relation, due to the nonzero exchange stiffness $D_\mathrm{ex} \neq 0$. The numerically calculated plateau frequency is approximately 6 MHz higher than the above analytical expression of f_p. Furthermore, the numerically calculated plateau frequency, not the analytical expression, is

the frequency that is approximately equal to the argument of the maximum of the noise spectrum in Fig. 2C within accuracy limited by the frequency binning ($3\,\mathrm{MHz}$) of Fig. 2C. Still, the conditions for the crossing in Fig. S14B qualitatively explains the black circles in Fig. S14A.

Furthermore, the decrease of the peak height of $\Delta(1/T_1)$ at H_c can be explained by the polarization of the magnetic noise caused by the thermally dirven MSSWs. Here, we recall that the NV center's longitudinal relaxation rate is associated with the magnetic noise perpendicular to the NV axis $\hat{n}_\mathrm{NV} \propto \mathbf{H}_{\|}$, with minor corrections due to the non-axial external magnetic field. The polarization of the magnon-induced magnetic field $\mathbf{h}_\mathrm{d}$ due to the magnetic dipole-dipole interaction is circular polarized (*11, 12*) ($\mathbf{h}_\mathrm{d} \propto \hat{\sigma}^{+}_{-\mathbf{k}} = \hat{n} - i\hat{k}$) in the plane spanned by the normal direction $\hat{n}$ of the YIG film and the direction of the magnon wave vector $\hat{k} = \mathbf{k}/|\mathbf{k}|$ [see Eq. (S224)]. Because the MSSW modes have the wave vector $\mathbf{k}$ perpendicular to $\mathbf{H}$, the magnetic noise vector component relevant to the NV center's longitudinal relaxation is maximized when $\mathbf{H} = \mathbf{H}_{\|}$ and $H_\perp = 0$. More concretely, the MSSW modes propagate in the direction of $\mathbf{k} \parallel \mathbf{H} \times \hat{n}$, and the magnetic field component relevant to the NV center's longitudinal relaxation for $|m_s = 0\rangle \leftrightarrow |m_s = -1\rangle$ is obtained by taking the inner product of the positive-frequency magnetic field with $(\hat{n} + i\hat{n}_\mathrm{NV} \times \hat{n})$. Here, the positive-frequency magnetic field is defined as the term with the time dependence taking the form of $\mathbf{h}_\mathrm{d}(t) \propto \mathbf{h}_\mathrm{d} e^{-i\omega t}$ with the magnon-mode frequency ω. Therefore, the thermal MSSW-induced magnetic field responsible for the longitudinal relaxation is given by

$$\begin{aligned}(\hat{n} + i\hat{n}_\mathrm{NV} \times \hat{n}) \cdot \mathbf{h}_\mathrm{d} &\propto (\hat{n} + i\hat{n}_\mathrm{NV} \times \hat{n}) \cdot \hat{\sigma}^{+}_{-\mathbf{k}}, \\ &= (\hat{n} + i\hat{n}_\mathrm{NV} \times \hat{n}) \cdot (\hat{n} - i(\mathbf{H} \times \hat{n})/|\mathbf{H}|) = 1 + \hat{n}_\mathrm{NV} \cdot \mathbf{H}/|\mathbf{H}|. \quad \text{(S31)}\end{aligned}$$

This confirms that the thermal-MSSW-induced magnetic field is maximized for $\hat{n}_\mathrm{NV} \parallel \mathbf{H}$. Therefore, the peak height of $\Delta(1/T_1)$ decreases when $H_\perp$ is increased.

SI.10 Discussion of the generalization of the cooperativity

Let us recall the definition of the cooperativity $\mathcal{C}_\mathrm{cav}$ of a two-level system coupled to a cavity mode (*13, 14*)

$$\mathcal{C}_\mathrm{cav} = |g_\mathrm{c}|^2 T_2^*/\kappa, \quad \text{(S32)}$$

where g_c is the coupling strength between the qubit and a cavity mode (or simply a single discretized mode), κ is the loss rate of the cavity [which makes the time-evolution of a cavity annihilation operator in the interaction picture to be $\langle a_c(t) \rangle \propto \exp(-\kappa t)$ and the energy decay rate to be 2κ], and T_2^* is the dephasing time of the qubit [e.g., the time-evolution of the transverse Pauli operator in the interaction-picture follows $\langle \sigma_-(t) \rangle \propto \exp(-t/T_2^*)$]. While this expression of the cooperativity is only a relevant parameter in describing a qubit-cavity system, its extension to a single waveguide mode (which can be described by a collection of modes labeled by a single continuous variable k, where k is a wavenumber) is widely used (*15–17*). The waveguide cooperativity $\mathcal{C}_{\rm wg}$ is given by

$$\mathcal{C}_{\rm wg} = \Gamma_{\rm 1D}/\Gamma', \tag{S33}$$

where $\Gamma_{\rm 1D}$ is the emission rate into the one-dimensional waveguide and Γ' is the emission rate into all other possible radiative channels.

While Eqs. (S32) and (S33) appear different, they are both related to the imaginary part of the susceptibility defined in Eq. (S287). In the limit where there is only one bosonic cavity mode coupled to the qubit, the susceptibility becomes

$$\chi_{B_-B_+}[\omega] = \frac{|g_{\rm c}|^2}{\omega_{\rm c} - \omega - i\kappa}, \tag{S34}$$

where we replaced $i0^+$ with $i\kappa$ in the denominator to account for the finite lifetime of the cavity boson. Therefore, the cavity cooperativity can be rewritten as

$$\mathcal{C}_{\rm cav} = \chi''_{B_-B_+}[\omega_{\rm c}] T_2^*, \tag{S35}$$

where the double prime indicates the imaginary part. Therefore, the cavity cooperativity $\mathcal{C}_{\rm cav}$ can be understood as a product of the imaginary part of the susceptibility and the spin decoherence time. Similarly, for the waveguide cooperativity $\mathcal{C}_{\rm wg}$, the emission rate into the waveguide can be written in terms of the emitter-waveguide coupling strength g_k based on Fermi's golden rule as

$$\Gamma_{\rm 1D} = 2\pi \sum_k |g_k|^2 \delta(\omega - \omega_k) = 2{\rm Im}\left[\sum_k \mathcal{P}\frac{|g_k|^2}{\omega_k - \omega} + i\pi |g_k|^2 \delta(\omega_k - \omega)\right] = 2\chi''_{B_-B_+}[\omega], \tag{S36}$$

where ω is the emitter frequency, ω_k is the waveguide frequency labeled by the wavenumber k, and we used the Plemelj formula $1/(\omega_k - \omega - i0^+) = \mathcal{P}(\omega_k - \omega)^{-1} + i\pi\delta(\omega_k - \omega)$ in the last equality.

Using Eq. (S36), the waveguide cooperativity can be rewritten as

$$\mathcal{C}_{\rm wg} = \chi''_{B_-B_+}[\omega](\Gamma'/2)^{-1}. \tag{S37}$$

We now argue that Eq. (S37) can be also understood as a product of the the imaginary part of the susceptibility and the spin decoherence time. This is because $\Gamma'/2$ in Eq. (S37) can also be understood to be related to the spin decoherence rate of the emitter based on the following argument. When the interaction between the emitter and the waveguide is small, the emission rate Γ' into other radiative channels except for the waveguide is approximately the same as the bare emission rate Γ_0 (i.e. the longitudinal relaxation rate without the waveguide). Recalling that the spin decoherence time T_2 is limited by twice the longitudinal relaxation time, i.e. $1/T_2 \geq \Gamma_0/2$, we can validate the relation $\Gamma'/2 \lesssim 1/T_2$. Therefore, Eq. (S37) can also be understood as the product of the susceptibility and the spin decoherence time.

As we report in Sec. SI.1, the (Ramsey) dephasing and the (Hahn) decoherence times of the ensemble of NV centers used in this work are $T_2^* = 182(5)$ ns and $T_2 = 3.53(5)$ μs, respectively. To quantify the NV-magnon interaction of our experimental system, we define the following generalization of the cooperativity

$$\mathcal{C}_{T_2^*} = \chi''(H)T_2^*, \tag{S38}$$

where χ'' is the imaginary part of the magnon-induced self-energy (same as the susceptibility, see Sec. SIII.6.4). We call it a generalization (or an extension) of the cooperativity because Eq. (S38) becomes the cavity cooperativity $\mathcal{C}_{\rm cav}$ (waveguide cooperativity $\mathcal{C}_{\rm wg}$) when we have a single boson mode (a single waveguide mode). Multiplying the maximum of the imaginary part of the self-energy $\chi''(H)$ in Fig. 4A ($\approx 2\pi \times 3.0$ Hz) and $T_2^* \approx 180$ ns, we obtain the generalized cooperativity of our system as $\mathcal{C}_{T_2^*} \approx 3.4\times10^{-6}$. If instead the Hahn-echo decoherence time $T_2 \approx 3.5$ μs is used, we obtain $\mathcal{C}_{T_2} \approx 6.6 \times 10^{-5}$. However, these coherence times are limited by the nearby P1 centers as discussed in Sec. SI.1. Using the best-known (to our knowledge) coherence time $T_{2,\rm ref}^* \approx 1$ ms (*18*), we obtain a projected generalized cooperativity as $\mathcal{C}_{\rm proj} \equiv \mathcal{C}_{T_{2,\rm ref}^*} \approx 0.02$. Importantly, YIG structures that couple to the NV center more efficiently will also increase cooperativity. For example, in Ref. (*14*) it is

calculated that a magnon mode of a small YIG micromagnet ($30\ \mathrm{nm} \times 3\ \mu\mathrm{m} \times 5\ \mathrm{nm}$) with $5\ \mathrm{nm}$ YIG-NV distance gives rise to $\mathcal{C}_{\mathrm{cav}} \approx 5 \times 10^4$ with the use of $T^*_{2,\mathrm{ref}} = 1\ \mathrm{ms}$, which is approximately 3×10^6 times larger than the projected generalized cooperativity $\mathcal{C}_{\mathrm{proj}} \approx 0.02$. The calculated value of the cooperativity in Ref. (*14*) corresponds to the imaginary part of the self-energy $\chi'' = \mathcal{C}_{\mathrm{cav}}/T^*_{2,\mathrm{ref}} \approx 2\pi \times 8 \times 10^6$ Hz, which is again approximately 3×10^6 times larger than what we obtained in Fig.4A using the 3-$\mu\mathrm{m}$-thick YIG film with $400\ \mathrm{nm}$ YIG-NV distance.

Furthermore, based on the scaling we obtain in Fig. S13 which is proportional to χ'' and using $\alpha = -2.4 \pm 0.1$, we estimate that the generalized cooperativity is approximately $10^{+2.4(1)} = 250^{+65}_{-52}$ times larger when h_{NV} is ten times smaller. However, in such conditions, $\Delta(1/T_1)$ is also 250 times larger, leading to $\Delta(1/T_1) \approx 250 \times 0.18\ \mu\mathrm{s}^{-1} = 45\ \mu\mathrm{s}^{-1}$, where $0.18\ \mu\mathrm{s}^{-1}$ is the maximum of $\Delta(1/T_1)$ at $h_{\mathrm{NV}} = 400\ \mathrm{nm}$ we observe in Fig. 2B. This is approximately equal to or larger than the theoretical upper bound we derived in Eq. (S30) under the assumption of our model NV-magnon couplings and dispersion relations proposed in Eqs. (S25) and (S26), indicating that the scaling behavior with $\alpha \approx -2.4$ may fail in such small h_{NV} regime. To explore the larger NV-magnon cooperativity in such conditions, lower temperature is necessary [we note that $\coth(\beta\omega_{\mathrm{NV}}/2) \approx 2k_{\mathrm{B}}T/\omega_{\mathrm{NV}}$ at high temperatures in Eq. (S27), where k_{B} is the Boltzmann constant and T is temperature] as the longitudinal decay rate of the NV centers on the YIG film at room temperature becomes much larger than the initialization rate of the NV centers with green lasers, making it difficult to initialize the NV centers.

SI.11 Kramers-Kronig analysis of the experimental data

We derive the equations connecting the longitudinal relaxation rate and the real part of the self-energy from the fluctuation-dissipation and Kramers-Kronig relation (KKR) in Sec. SIV as

$$\chi'(H) \approx \mathcal{P}\int_{-\infty}^{\infty} \frac{dH'}{\pi}\frac{\chi''(H')}{H-H'}, \tag{S39}$$

$$\chi''(H) = \frac{1/T_1(H)}{2\coth(\beta\omega_{\mathrm{NV}}(H)/2)}, \tag{S40}$$

where $\mathcal{P}$ indicates the Cauchy principal value and $1/T_1(H)$ is the longitudinal relaxation rate at the external magnetic field H. To apply Eqs. (S39) and (S40) into the experimental data, we replace

$1/T_1$ by $\Delta(1/T_1)$ to eliminate the offset contributions. We note that $T_1(H)$ in the range $H < 0$ is not experimentally accessible, as the integration in the range $H < 0$ in Eq. (S39) is simply extended for mathematical convenience [see Eq. (S336)]. Additionally, in real experiments, it is impossible to obtain the full spectrum even in the range $H > 0$, which is always the case with any experimental analysis using the KKR (*19–22*). Typically, some sort of extrapolation of the data is performed (*19,20*) when the full spectrum is not available. However, in our experimental analysis, we did not perform any extrapolation of the data. We write $\mathcal{R}_H$ as the experimentally accessible field range. In our experimental analysis, we simply set $\chi''(H) = 0$ outside $\mathcal{R}_H$ because the spectrum of the longitudinal relaxation rate $\Delta(1/T_1(H))$ almost vanishes both at the beginning and at the end of $\mathcal{R}_H$. This eliminates the possibility of obtaining poor estimation of $\chi'(H)$, especially near the edges of $\mathcal{R}_H$, caused by the inaccessible but nonzero contribution of $\chi''(H)$ right outside of the range $\mathcal{R}_H$, though the extrapolated data could be used there if needed. Here, we note that if the major part of the noise spectrum is not captured by the experiment, the extrapolation (*19, 20*) of the spectrum is no longer valid and the KKR analysis will fail. Therefore, the underlying assumption for the KKR analysis presented in this section is that the experiment captures a large enough part of the noise spectrum and the inaccessible part of the spectrum can be either extrapolated or simply ignored.

For the analysis of the experimental data, we had $\mathcal{R}_H = \{H|30\text{ G} \leq \mu_0 H \leq 600\text{ G}\}$. The numerical evaluation of the KKR Eq. (S39) is performed similarly to Refs. (*23,24*) using the following digitized numerical KKR with the Taylor expansion

$$\chi'(H_i) = \sum_j \int_{(H_j+H_{j-1})/2}^{(H_j+H_{j+1})/2} \frac{dH'}{\pi} \mathcal{P} \frac{\chi''(H_j) + (H' - H_j)\partial_H \chi''(H_j)}{H_i - H'}, \tag{S41}$$

where H_i is the discretized field, $\partial_H \chi''(H_j)$ is the estimated slope calculated by

$$\chi''(H_j) = r\frac{\chi''(H_{j+1}) - \chi''(H_j)}{H_{j+1} - H_j} + (1-r)\frac{\chi''(H_j) - \chi''(H_{j-1})}{H_j - H_{j-1}}, \tag{S42}$$

with $r = (H_j - H_{j-1})/(H_{j+1} - H_{j-1})$, and the sum over j is performed for all the fields H_j at which we measured T_1 of the NV center, i.e.

$$\mu_0 H_j \in \{30\text{ G}, 40\text{ G}, \cdots, 70\text{ G}, 71\text{ G}, \cdots, \\ 95\text{ G}, 100\text{ G}, 120\text{ G}, 150\text{ G}, \cdots, 480\text{ G}, 540\text{ G}, 570\text{ G}, 600\text{ G}\}. \tag{S43}$$

We note, however, that Eq. (S41) is not identical to what is presented in Refs. (*23, 24*). They show numerical KKR for the case with evenly sampled data set, and the direct application of the trapezium formula presented in Refs. (*23, 24*) is nontrivial. It would be

$$\chi'(H_i) = \sum_{j\neq i} \frac{\Delta H_j}{\pi} \frac{\chi''(H_j)}{H_i - H_j} + \int_{(H_i+H_{i-1})/2}^{(H_i+H_{i+1})/2} \frac{dH'}{\pi} \mathcal{P} \frac{\chi''(H_i) + (H' - H_i)\partial_H \chi''(H_i)}{H_i - H'}, \tag{S44}$$

where $\Delta H_j = (H_{j+1} - H_{j-1})/2$, the first term is the standard numerical integration contribution in the range without the pole, and the second term is the pole contribution of the principal value integral. The numerator of the integrand is Taylor expanded up to the linear order, as the zeroth-order term vanishes when the data is taken uniformly, i.e. $\Delta H_j = (\text{constant})$. In our analysis, however, the use of Eq. (S44) results in nonphysical artifacts at $\mu_0 H_i = 71$ G and $\mu_0 H_i = 94$ G due to the non-uniform sampling of the data. This is shown in Fig. S15A, where the black markers show spikes at 71 G and 94 G. To avoid this artifact, in Eq. (S41), the first term of Eq. (S44) is also evaluated using the Taylor expanded integral, which removes the case structure of the sum over $j \neq i$ and $j = i$. To validate this approach, we compute $\chi'(H)$ using methods presented in Ref. (*24*) (i.e. the trapezium formula and Maclaurin's formula) after the quadratic interpolation of our experimental data in the field range outside the finest scanned field range (i.e. $\mu_0 H < 70$ G and 95 G $< \mu_0 H$), with $\Delta H_j = 1$ G$/\mu_0$ step, which is the smallest field step in the experiment. We show the results of the interpolated trapezium formula and the interpolated Maclaurin's formula in Fig. S15B with blue upward triangles and green downward triangles, respectively. We find that the differences in these results from the one obtained by Eq. (S41) are small (both less than 2% using the l^2-norm as a metric). In contrast, the difference between the results obtained by the direct application of Eq. (S44) without the interpolation and Eq. (S41) is much larger (17 %). This supports that the features at 71 G and 94 G obtained by the direct application of the trapezium formula Eq. (S44) (the black markers in Fig. S15A) are nonphysical artifacts due to the non-even sampling of the data, and validates the use of Eq. (S41) in our analysis. We note that the direct application of the Maclaurin's formula provided in Ref. (*24*) is even more nontrivial for our non-evenly sampled data according to the way it is defined.

SI.12 Ratio of $g_{\rm eff}$ and $g_{\rm dip}$

In Fig. 4B, we show the calculated NV-NV distance r dependence of $g_{\rm eff}$ and $g_{\rm dip}$, where $g_{\rm dip}$ is given by (writing $\hbar = 1$ more explicitly here to avoid confusion)

$$\hbar g_{\rm dip} = -\frac{\mu_0 \gamma^2 \hbar^2}{4\pi r^3}. \tag{S45}$$

The amplitude of $g_{\rm dip}$ characterises the magnetc dipole-dipole interaction strength between two NV centers separated by r, as the magnetic dipole-dipole interaction Hamiltonian $\mathcal{H}_{\rm dip}$ between two NV centers (NV_1 and NV_2) separated by a distance r in the direction of $\hat{z} = \hat{n}_{\rm NV} \parallel \mathbf{H}_{\parallel}$ [see the illustration in Fig. 4B] is given by

$$\begin{aligned}\mathcal{H}_{\rm dip} &= \frac{\mu_0\gamma^2}{4\pi}\frac{\mathbf{S}_{\mathrm{NV}_1}\cdot\mathbf{S}_{\mathrm{NV}_2} - 3(\hat{z}\cdot\mathbf{S}_{\mathrm{NV}_1})(\hat{z}\cdot\mathbf{S}_{\mathrm{NV}_2})}{r^3} \\ &= -\hbar g_{\rm dip}(S^-_{\mathrm{NV}_1}S^+_{\mathrm{NV}_2} + S^+_{\mathrm{NV}_1}S^-_{\mathrm{NV}_2} - 4S^z_{\mathrm{NV}_1}S^z_{\mathrm{NV}_2})/2,\end{aligned} \tag{S46}$$

where $\mathbf{S}_{\mathrm{NV}_i}$ is the spin-1 operator of the NV center labeled by $i = 1, 2$. The projection of this Hamiltonian onto the subspace spanned by the lowest two energy eigenstates of each NV center is given by

$$\mathbb{P}\mathcal{H}_{\rm dip}\mathbb{P} = -g_{\rm dip}(\sigma^+_{\mathrm{NV}_1}\sigma^-_{\mathrm{NV}_2} + \sigma^-_{\mathrm{NV}_1}\sigma^+_{\mathrm{NV}_2}) + 2g_{\rm dip}|e,e\rangle\langle e,e|, \tag{S47}$$

where $\sigma^+_{\mathrm{NV}_i} = |e\rangle_i\langle g|$, $\sigma^-_{\mathrm{NV}_i} = (\sigma^+_{\mathrm{NV}_i})^\dagger$, $|\alpha,\beta\rangle = |\alpha\rangle_1|\beta\rangle_2$ for $\alpha,\beta \in \{e,g\}$, $|g\rangle_i$ ($|e\rangle_i$) is the ground (excited) state of the NV_i's electron spin, and $\mathbb{P}$ is the projection operator onto the subspace spanned by $\{|g,g\rangle, |e,g\rangle, |g,e\rangle, |e,e\rangle\}$. In contrast, the magnon-mediated NV-NV interaction Hamiltonian takes the form of [see Eq. (S357)]

$$\mathcal{H}_{\rm eff} = -g_{\rm eff}(\sigma^+_{\mathrm{NV}_1}\sigma^-_{\mathrm{NV}_2} + \sigma^-_{\mathrm{NV}_1}\sigma^+_{\mathrm{NV}_2}), \tag{S48}$$

where we used the condition that $g_{\rm eff}$ is a real number. While the magnon-mediated coupling $g_{\rm eff}$ is in general a complex number [see Eq. (S357)], it becomes a real number in the geometry shown in the illustration of Fig.4B. Comparing Eqs. (S47) and (S48), it is reasonable to compare $g_{\rm eff}$ and $g_{\rm dip}$ in Fig. 4B. Furthermore, we show in Fig. S16 the ratio of $g_{\rm eff}$ and $g_{\rm dip}$ as a function of r, which can

be calculated from the curves in Fig. 4B. For the magnon-mediated NV-NV interaction to be useful, it needs to bigger than one in the optically resolvable distance, or larger than the diffraction limit, on the order of a micrometer. The non-monotonic behavior of the ratio for the curves at 82 G and 83 G magnetic field conditions are due to the node of the oscillation of the magnon-mediated coupling g_{eff} as a function of the distance (see Fig. 4B).

SII Relationship between NV-magnon coupling and surface-magnon mediated efficient driving of NV centers

In this section, we show that the surface-magnon mediated efficient long-distance driving of NV centers can be understood in terms of the combination of NV-magnon and magnon-MSL (microstrip line) couplings, as mentioned in the main text. Based on Eqs. (11) (12), (8), and (7) of Ref. (*25*), the magnetic field $\mathbf{H}^{\mathrm{I}}$ at the NV position generated by a MSL with width w carrying a current $I = I_0 e^{-i\omega t}$ (oscillating with frequency ω) is

$$\boldsymbol{H}^{\mathrm{I}}(y,h) = \begin{pmatrix} H_y^{\mathrm{I}}(y,h) \\ H_x^{\mathrm{I}}(y,h) \end{pmatrix} = \begin{pmatrix} 1 \\ i \end{pmatrix} H^{\mathrm{I}}(y,h)/\sqrt{2}, \tag{S49}$$

$$H^{\mathrm{I}}(y,h) = \frac{1}{\sqrt{2\pi}} \int_0^\infty H^{\mathrm{I}}(k,h)\, e^{iky} dk, \tag{S50}$$

$$H^{\mathrm{I}}(k,h)/\sqrt{2} = C_1(k)\, e^{-kh}, \tag{S51}$$

$$C_1(k) = j_0 \frac{\sin(kw/2)}{k} \frac{\omega_M(\omega_H + \omega_M + \omega) - e^{2kd}(\omega_H + \omega_M - \omega)(2\omega_H + \omega_M + 2\omega)}{\omega_M^2 + e^{2kd}\left[4\omega^2 - (2\omega_H + \omega_M)^2\right]}, \tag{S52}$$

where $j_0 = (2/\pi)^{1/2} I_0/w$ is the surface current density, k is the wave number, ω is the applied microwave frequency, d is the thickness of the YIG film, $h = x - d/2$ is the NV-YIG distance, $\omega_H = \gamma\mu_0 H_{\mathrm{ext}}$, $\omega_M = \gamma\mu_0 M_{\mathrm{s}}$, γ is the absolute value of the electronic gyromagnetic ratio, μ_0 is the vacuum permeability, M_{s} is the saturation magnetization, and H_{ext} is the applied external magnetic field. Here, we rewrite $(x, z, \hat{x}, \hat{z}, k_x, \omega_0)$ in Ref. (*25*) into $(y, h, \hat{y}, \hat{x}, k, \omega_H)$ to match our notation and the coordinate system as shown in Figs. S17A and B. At the NV position $(y,h) = (y_0, h_0)$, the circular component of the magnetic field generated by magnons is

$$H_+^{\mathrm{I}}(y_0,h_0) = H_x(y_0,h_0) + iH_y(y_0,h_0) = \int_0^\infty \frac{dk}{2\pi}\left[2i\sqrt{2\pi}C_1(k)e^{-kh_0}\right]e^{iky_0}. \tag{S53}$$

After some algebra to simplify the term $2i\sqrt{2\pi}C_1(k)e^{-kh_0}$, the field at the NV position becomes

$$H_+^{\mathrm{I}}(y_0,h_0) = \int_0^\infty \frac{dk}{2\pi}\left[\left(\mathcal{A}_{+k}\mathcal{F}_{+k}\frac{i}{\omega - \omega_0(k)} + \mathcal{A}_{-k}\mathcal{F}_{-k}\frac{i}{\omega + \omega_0(k)}\right) I_0 + iI_0\frac{\sin(kw/2)}{kw/2}e^{-kh_0}\right]e^{iky_0}, \tag{S54}$$

where the third term is the direct MSL induced magnetic field [see Eq. (S146)]. Here, $A_{\nu k}$ is the NV-magnon coupling and $F_{\nu k}$ is the magnon-MSL coupling with $\nu = \pm 1$, which appear in the NV-

MSSW (magnetostatic surface spin waves, or surface magnons) interaction Hamiltonian $\mathcal{H}_{\rm int}^{\mathbf{k}\parallel\pm\hat{y}}$ and MSL-magnon interaction Hamiltonian $\mathcal{H}_{\rm MSL}$, respectively,

$$\mathcal{H}_{\rm int}^{\mathbf{k}\parallel\pm\hat{y}} = \frac{\gamma\mu_0}{2L}\sum_{k\geq 0}[S_{\rm NV}^{-}(\mathcal{A}_{+k}\beta_{+k,0} + \mathcal{A}_{-k}\beta_{-k,0}^{\dagger})e^{iky_0} + \text{H.c.}], \tag{S55}$$

$$\mathcal{H}_{\rm MSL} = -\sum_{\nu,k} i[\mathcal{F}_{\nu k}(\alpha_{\nu k,0} - \alpha_{\nu k,0}^{\dagger})]I, \tag{S56}$$

where the superscript $\mathbf{k} \parallel \pm\hat{y}$ in Eq. (S55) indicates that the Hamiltonian is for magnons propagating in $\pm\hat{y}$ direction (i.e. MSSW), $L \to \infty$ is the length of the YIG film in the in-plane directions ($\hat{y}$ and $\hat{z}$) used for the periodic boundary condition, the discrete sum of k is taken for $k = (\text{integer}) \times 2\pi/L$, we have $\sum_k = L\int dk/2\pi$ following the standard notation (*26*), $\beta_{\pm k,0}^{\dagger}$ ($\beta_{\pm k,0}$) is the creation (annihilation) operator of MSSWs, with wave vector $\pm k\hat{y}$, I is the current in the MSL, and we set $\hbar = 1$. Eqs. (S55) and (S56) will be derived in the following sections.

SII.1 Second quantization of the magnetostatic surface spin waves

In this section, we perform a canonical quantization of the MSSWs in ferromagnets [and ferrimagnets such as yttrium iron garnet, YIG, which has been widely known as a standard material for studying MSSWs (*26, 27*)]. We apply an external magnetic field along the $\hat{z}$ direction, $\mathbf{H}_{\rm ext} = H_{\rm ext}\hat{z}$, where the MSSWs are the waves propagating along $\pm\hat{y}$ direction with surface localization. The magnetization $\mathbf{M}(\mathbf{r})$ exist in $-d/2 \leq x \leq d/2$. The magnon Hamiltonian $\mathcal{H}_{\rm m}$ within the magnetostatic approximation is given by

$$\mathcal{H}_{\rm m} = -\mu_0\int d\mathbf{r}\mathbf{H}_{\rm ext}\cdot\mathbf{M}(\mathbf{r}) + \frac{\mu_0}{2}\int d\mathbf{r}d\mathbf{r}'(\nabla\cdot\mathbf{M}(\mathbf{r}))G\left(\mathbf{r}-\mathbf{r}'\right)\left(\nabla'\cdot\mathbf{M}\left(\mathbf{r}'\right)\right), \tag{S57}$$

where $G(\mathbf{r}-\mathbf{r}') = 1/4\pi|\mathbf{r}-\mathbf{r}'|$ is the Green's function for the (negative of the) Laplacian $-\nabla^2 G(\mathbf{r}-\mathbf{r}') = \delta(\mathbf{r}-\mathbf{r}')$. At equilibrium, $\mathbf{M}(\mathbf{r})$ is along the $\hat{z}$ axis, $\mathbf{M}(\mathbf{r}) = \mathbf{M}_0(\mathbf{r}) = M_{\rm s}(x)\hat{z}$, where $M_{\rm s}(x) = M_{\rm s}\mathcal{F}(x)$, $M_{\rm s}$ is the YIG saturation magnetization, $\mathcal{F}(x) = \Theta(x+d/2)\Theta(d/2-x)$ is one (zero) inside (outside) the ferromagnet, and $\Theta(\cdots)$ is the Heaviside step function. We denote the variation of $\mathbf{M}(\mathbf{r})$ with respect to the equilibrium magnetization as $\delta\mathbf{M}(\mathbf{r}) = \mathbf{M}(\mathbf{r}) - \mathbf{M}_0(\mathbf{r})$. Using the Holstein-Primakoff transformation [see Ref. (*27*) and Eqs. (S171)-(S173)], up to quadratic order

in the complex canonical variables $a(\mathbf{r})$ and $a^*(\mathbf{r})$ (*26*), we can write

$$\delta M_x(\mathbf{r}) \approx m_x(\mathbf{r}), \tag{S58}$$

$$\delta M_y(\mathbf{r}) \approx m_y(\mathbf{r}), \tag{S59}$$

$$\delta M_z(\mathbf{r}) \approx M_\mathrm{s}(x) - \frac{\mathbf{m}(\mathbf{r}) \cdot \mathbf{m}(\mathbf{r})}{2M_\mathrm{s}(x)}, \tag{S60}$$

where $\mathbf{m}(\mathbf{r}) = m_x(\mathbf{r})\hat{x} + m_y(\mathbf{r})\hat{x}$ is linear in the complex canonical variables a and a^*, known as the two-dimensional linearized magnetization deviation obeying

$$m_-(\mathbf{r}) = m_x(\mathbf{r}) - im_y(\mathbf{r}) = \sqrt{2\gamma M_\mathrm{s}(x)}a(\mathbf{r}), \tag{S61}$$

$$m_+(\mathbf{r}) = m_x(\mathbf{r}) + im_y(\mathbf{r}) = \sqrt{2\gamma M_\mathrm{s}(x)}a^*(\mathbf{r}). \tag{S62}$$

We perform the two-dimensional Fourier transform for the in-plane position $\boldsymbol{\rho} = y\hat{y} + z\hat{z}$ with a wave vector $\mathbf{k} = k_y\hat{y} + k_z\hat{z}$, namely

$$\mathbf{m}(\mathbf{r}) = \sum_{\mathbf{k}} \frac{e^{i\mathbf{k}\cdot\boldsymbol{\rho}}}{\sqrt{L^2}} \mathbf{m}_{\mathbf{k}}(x), \tag{S63}$$

where $\sqrt{L^2} = \sqrt{(\text{Area})}$ in the denominator makes the basis function $e^{i\mathbf{k}\cdot\boldsymbol{\rho}}/\sqrt{L^2}$ normalized. Then, we obtain the magnon Hamiltonian $\mathcal{H}_\mathrm{m} = \int dx \sum_{\mathbf{k}} \mathcal{H}_\mathrm{m}^{\mathbf{k}}(x)$ with

$$\mathcal{H}_\mathrm{m}^{\mathbf{k}}(x) \approx \frac{\mu_0}{2} \left[\frac{H_\mathrm{ext}}{M_\mathrm{s}(x)} \mathbf{m}_{-\mathbf{k}}(x) \cdot \mathbf{m}_{\mathbf{k}}(x) - \mathbf{m}_{-\mathbf{k}}(x) \cdot \int dx' \hat{D}_{\mathbf{k}}(x - x') \cdot \mathbf{m}_{\mathbf{k}}(x') \right], \tag{S64}$$

where $\hat{D}_{\mathbf{k}}(x - x')$ is the Fourier representation of the dipole tensor:

$$\hat{D}_{\mathbf{k}}(x - x') = \frac{ke^{-k|x-x'|}}{2} \hat{\sigma}_{\mathbf{k}}^{-\mathrm{sgn}(x-x')} \otimes \hat{\sigma}_{\mathbf{k}}^{-\mathrm{sgn}(x-x')} - \hat{x} \otimes \hat{x}\delta(x - x'), \tag{S65}$$

$$\hat{\sigma}_{\mathbf{k}}^{\pm} = \hat{x} \pm i\hat{k} = \hat{x} \pm i\mathbf{k}/|\mathbf{k}|. \tag{S66}$$

The Fourier transform for the complex canonical variables is defined as

$$a(\mathbf{r}) = \sum_{\mathbf{k}} \frac{e^{i\mathbf{k}\cdot\boldsymbol{\rho}}}{\sqrt{L^2}} a_{\mathbf{k}}(x), \tag{S67}$$

$$a^*(\mathbf{r}) = \sum_{\mathbf{k}} \frac{e^{-i\mathbf{k}\cdot\boldsymbol{\rho}}}{\sqrt{L^2}} a_{\mathbf{k}}^*(x), \tag{S68}$$

which leads to the following relation between the Fourier components of the magnetization deviation and the Fourier components of the canonical variables

$$m^{-}_{\mathbf{k}}(x) = \sqrt{2\gamma M_{\rm s}(x)}a_{\mathbf{k}}(x), \tag{S69}$$

$$m^{+}_{\mathbf{k}}(x) = \sqrt{2\gamma M_{\rm s}(x)}a^{*}_{-\mathbf{k}}(x). \tag{S70}$$

The transformation is made such that when promoting the complex canonical variables to the quantum creation and annihilation operators, we have the commutation relation $[a_{\mathbf{k}}(x), a^{\dagger}_{\mathbf{k}'}(x')] = \delta_{\mathbf{k},\mathbf{k}'}\delta(x - x')$.

To obtain the second-quantized form of MSSW modes, we focus on the their corresponding momentum sector $\mathbf{k} = k_y\hat{y} = \nu k\hat{y}$ with $k = |\mathbf{k}|$ and $\nu = \pm 1$. We perform the following classical Bogoliubov transformation to define new complex canonical variables $\beta^{*}_{\nu k,\mu}$ and $\beta_{\nu k,\mu}$

$$a_{\nu k\hat{y}}(x) = \sum_{\mu=0}^{\infty}\left[\xi^{(\mu)}_{\nu k}(x)\beta_{\nu k,\mu} + \eta^{(\mu)}_{-\nu k}(x)\beta^{*}_{-\nu k,\mu}\right], \tag{S71}$$

$$a^{*}_{-\nu k\hat{y}}(x) = \sum_{\mu=0}^{\infty}\left[\eta^{(\mu)}_{\nu k}(x)\beta_{\nu k,\mu} + \xi^{(\mu)}_{-\nu k}(x)\beta^{*}_{-\nu k,\mu}\right], \tag{S72}$$

where their inverses are given by

$$\beta_{\nu k,\mu} = \int dx\left[\xi^{(\mu)}_{\nu k}(x)a_{\nu k\hat{y}}(x) - \eta^{(\mu)}_{\nu k}(x)a^{*}_{-\nu k\hat{y}}(x)\right], \tag{S73}$$

$$\beta^{*}_{-\nu k,\mu} = \int dx\left[-\eta^{(\mu)}_{-\nu k}(x)a_{\nu k\hat{y}}(x) + \xi^{(\mu)}_{-\nu k}(x)a^{*}_{-\nu k\hat{y}}(x)\right]. \tag{S74}$$

The functions $\xi^{(\mu)}_{\nu k}(x)$ and $\eta^{(\mu)}_{\nu k}(x)$ satisfy proper orthogonality relations (for detail, see Secs. SII.1.1-SII.1.3). The subscript/superscript μ labels the x-directional (thickness-directional) modes where the surface localized mode (MSSW) corresponds to $\mu = 0$. The modes with $\mu = 1, 2, \cdots$ are orthogonal to the MSSW mode under the following map (which is an inner product when applied to the vector space of positive-frequency solutions) involving the integral over x [see Ref. (*28*) and Sec. SII.1.3]

$$\begin{aligned}((\mathbf{m}_{\mathbf{k},\mu_1}, \mathbf{m}_{\mathbf{k},\mu_2})) &\equiv +i\int dx\left(\bar{m}^{x}_{\mathbf{k},\mu_1}(x)m^{y}_{\mathbf{k},\mu_2}(x) - \bar{m}^{y}_{\mathbf{k},\mu_1}(x)m^{x}_{\mathbf{k},\mu_2}(x)\right)/\gamma M_{\rm s}(x), \\ &= \int dx\left(\bar{m}^{-}_{\mathbf{k},\mu_1}(x)m^{-}_{\mathbf{k},\mu_2}(x) - \bar{m}^{+}_{\mathbf{k},\mu_1}(x)m^{+}_{\mathbf{k},\mu_2}(x)\right)/2\gamma M_{\rm s}(x),\end{aligned} \tag{S75}$$

where $\mathbf{m}_{\mathbf{k},\mu}(x)$ is the magnon-mode function labeled by $(\mathbf{k},\mu)$ [i.e. $m^{-}_{\nu k\hat{y},\mu}(x) = \sqrt{2\gamma M_{\rm s}(x)}\xi^{(\mu)}_{\nu k}(x)$ and $m^{+}_{\nu k\hat{y},\mu}(x) = \sqrt{2\gamma M_{\rm s}(x)}\eta^{(\mu)}_{\nu k}(x)$] and $\overline{\cdots}$ indicates the complex conjugation to avoid confusion with the asterisk in the complex canonical variables. With this transformation, the ferromagnet Hamiltonian Eq. (S64) can be written as

$$\int dx \sum_{\mathbf{k}=\pm k\hat{y}} \mathcal{H}^{\mathbf{k}}_{\rm m}(x) = \sum_{\nu,\mu} \omega_\mu(k)\beta^{*}_{\nu k,\mu}\beta_{\nu k,\mu}, \tag{S76}$$

where the energy of the MSSW (i.e. the mode with $\mu = 0$) is given by

$$\omega_0(k) = \sqrt{\omega_H(\omega_H + \omega_M) + \omega_M^2(1 - e^{-2kd})/4}. \tag{S77}$$

Promoting the complex canonical variables to the quantum creation and annihilation operators, we obtain the magnon Hamiltonian for $\mathbf{k} \parallel \pm\hat{y}$ as

$$\mathcal{H}^{\mathbf{k}\parallel\pm\hat{y}}_{\rm m} = \int dx \sum_{\mathbf{k}\parallel\pm\hat{y}} \mathcal{H}^{\mathbf{k}}_{\rm m}(x) = \sum_{k,\nu,\mu} \omega_\mu(k)\beta^{\dagger}_{\nu k,\mu}\beta_{\nu k,\mu}, \tag{S78}$$

where the commutation relation of the creation and annihilation operators is $[\beta_{\nu k,\mu}, \beta^{\dagger}_{\nu' k',\mu'}] = \delta_{k,k'}\delta_{\nu,\nu'}\delta_{\mu,\mu'}$. We note that there is a limitation in this treatment and this Hamiltonian will fail to capture the reality in a large momentum regime because the classical description of the magnetization is only accurate in the continuum limit $k \ll 1/a_{\rm F}$, where $a_{\rm F}$ is the spacing of the ferromagnetic spins. In addition, we have ignored the exchange interaction in the Hamiltonian, which is valid only for $D_{\rm ex}k^2 \ll \omega_M$, where $D_{\rm ex}$ is the exchange stiffness (for YIG, $D_{\rm ex}/\omega_M \approx 3 \times 10^{-4}\ \mu{\rm m}^2$). We also note that the magnetostatic approximation implies $k \gg \omega/c$, where c is the speed of light and ω is the spin-wave frequency. Accordingly, the currently studied model only describes accurately the real material when $\omega/c \ll k \ll \sqrt{\gamma D_{\rm ex}/\omega_M}$.

SII.1.1 Bogoliubov transformation of the MSSW

The functions that define the Bogoliubov transformation in Eqs. (S71) and (S72) are given by

$$\xi^{(\mu)}_{\nu k}(x) = \begin{cases} \frac{-1}{\sqrt{(\chi_0^2-\kappa_0^2)4\kappa_0}}(\chi_0+\kappa_0)q_+ h_{\nu k}(x) & ,\mu=0, \\ \frac{-1}{\sqrt{4\chi_\mu\kappa_\mu}}(\chi_\mu+\kappa_\mu)f_{\mu,\nu k}(x) & ,\mu\in(2\mathbb{Z}_+-1), \\ \frac{-1}{\sqrt{4\chi_\mu\kappa_\mu}}(\chi_\mu+\kappa_\mu)g_{\mu,\nu k}(x) & ,\mu\in 2\mathbb{Z}_+, \end{cases} \tag{S79}$$

$$\eta^{(\mu)}_{\nu k}(x) = \begin{cases} \frac{-1}{\sqrt{(\chi_0^2-\kappa_0^2)4\kappa_0}}(\chi_0-\kappa_0)q_- h_{-\nu k}(x) & ,\mu=0, \\ \frac{-1}{\sqrt{4\chi_\mu\kappa_\mu}}(\chi_\mu-\kappa_\mu)f_{\mu,-\nu k}(x) & ,\mu\in(2\mathbb{Z}_+-1), \\ \frac{-1}{\sqrt{4\chi_\mu\kappa_\mu}}(\chi_\mu-\kappa_\mu)g_{\mu,-\nu k}(x) & ,\mu\in 2\mathbb{Z}_+, \end{cases} \tag{S80}$$

$$q_\nu = \sqrt{(\chi_0+2-\nu\kappa_0)(\chi_0-\nu\kappa_0)}; \quad \chi_\mu = \frac{\omega_H\omega_M}{\omega_H^2-\omega_\mu^2}; \quad \kappa_\mu = \frac{\omega_\mu\omega_M}{\omega_H^2-\omega_\mu^2}, \tag{S81}$$

$$\omega_\mu(k) = \begin{cases} \sqrt{\omega_H(\omega_H+\omega_M)+\omega_M^2(1-e^{-2kd})/4}, & \mu=0, \\ \sqrt{\omega_H(\omega_H+\omega_M)} & ,\mu\neq 0, \end{cases} \tag{S82}$$

with $\mathbb{Z}_+$ being the set of positive integers. Here, $\mu=0$ corresponds to the MSSW mode and the modes with $\mu=1,2,\cdots$ are orthogonal to the MSSW mode. It turns out that the modes with $\mu=1,2,\cdots$ does not generate magnetic field outside the ferromagnet. It may be worth noting that the magnetic potential $\phi^{(\mu)}_{\mathrm{m}}(\mathbf{r})$ (*27*) for these modes are of form

$$\phi^{(\mu)}_{\mathrm{m}}(\mathbf{r}) \propto e^{i\nu ky}\Theta(x+d/2)\Theta(d/2-x)\begin{cases} \cos(\mu\pi x/d), \mu\in(2\mathbb{Z}_+-1), \\ \sin(\mu\pi x/d), \mu\in 2\mathbb{Z}_+, \end{cases} \tag{S83}$$

which leads to the zero magnetic field $\mathbf{h}^{(\mu)}_{\mathrm{d}}(\mathbf{r}) = -\nabla\phi^{(\mu)}_{\mathrm{m}}(\mathbf{r})$, outside the YIG film. The existence of these modes are depicted, for example, in Ref. (*29*). Although they are not paid attention often because they are not important as they do not couple to external magnetic fields, they are essential for the set of functions to be complete in the transformation. We further note that the prefactors of the functions in Eqs. (S79) and (S80) for the $\mu=0$ case satisfy

$$\left[((\chi_0^2-\kappa_0^2)4\kappa_0)^{-1/2}(\chi_0+\kappa_0)q_+\right]^2 - \left[((\chi_0^2-\kappa_0^2)4\kappa_0)^{-1/2}(\chi_0-\kappa_0)q_-\right]^2 = 1, \tag{S84}$$

which can be used to check the normalization. In the large wavenumber limit ($kd \gg 1$), we obtain

$$((\chi_0^2-\kappa_0^2)4\kappa_0)^{-1/2}(\chi_0+\kappa_0)q_+ \to 1, \tag{S85}$$

$$((\chi_0^2-\kappa_0^2)4\kappa_0)^{-1/2}(\chi_0-\kappa_0)q_- \to 0. \tag{S86}$$

This indicates that the magnon-mode functions are purely exponential for a large wavenumber k and $\mu = 0$ based on Eqs. (S79) and (S80) with the following definition of $h_{\nu k}(x)$. The functions $\{f_{\mu,\nu k}(x), g_{\mu,\nu k}(x), h_{\nu k}(x)\}$ are non-zero only when $-d/2 \leq x \leq d/2$ and are given by

$$f_{\mu,\nu k}(x) = \sqrt{\frac{2\mathcal{F}(x)d}{d^2k^2 + \mu^2\pi^2}} \left(\frac{\mu\pi}{d} \sin\frac{\mu\pi x}{d} - \nu k \cos\frac{\mu\pi x}{d}\right), \quad \text{(S87)}$$

$$g_{\mu,\nu k}(x) = \sqrt{\frac{2\mathcal{F}(x)d}{d^2k^2 + \mu^2\pi^2}} \left(\frac{\mu\pi}{d} \cos\frac{\mu\pi x}{d} + \nu k \sin\frac{\mu\pi x}{d}\right), \quad \text{(S88)}$$

$$h_{\nu k}(x) = \sqrt{\frac{\mathcal{F}(x)}{d}\frac{kd}{\sinh kd}} e^{\nu k x}. \quad \text{(S89)}$$

We note that as we are considering the case without the exchange interaction, we do not need to impose any additional surface-spin pinning conditions unlike Sec. SIII. The only condition we use is that the magnon-mode profile function is zero outside the ferromagnet (i.e. $x > d/2$ or $-d/2 > x$), and boundary conditions arising from the Maxwell's equations can be derived from the dipole integral kernel $\hat{D}_{\mathbf{k}}(x - x')$ (see Sec. SII.1.4). This is because the Hamiltonian equations of motion derived from Eq. (S64) is an integral equation (in spatial coordinates), not a differential equation. The set of functions $\{f_{2n-1,\nu k}(x), g_{2n,\nu k}(x), h_{\nu k}(x) | n \in \mathbb{Z}_+\}$ forms a complete orthonormal basis in $d/2 \geq x \geq -d/2$ with the conventional inner product $\langle\phi|\phi'\rangle = \int dx \phi(x)\phi'(x)$ (see Sec. SII.1.5). Using this orthogonality relation, we can verify that the functions $\xi^{(\mu)}_{\nu k}(x)$ and $\eta^{(\mu)}_{\nu k}(x)$ satisfy

$$\int_{-d/2}^{d/2} dx \left[\xi^{(\mu)}_{\nu k}(x)\xi^{(\mu')}_{\nu k}(x) - \eta^{(\mu)}_{\nu k}(x)\eta^{(\mu')}_{\nu k}(x)\right] = \delta_{\mu,\mu'} \quad \text{(S90)}$$

$$\int_{-d/2}^{d/2} dx \left[\xi^{(\mu)}_{\nu k}(x)\eta^{(\mu')}_{-\nu k}(x) - \eta^{(\mu)}_{\nu k}(x)\xi^{(\mu')}_{-\nu k}(x)\right] = 0, \quad \text{(S91)}$$

which can be used to check the commutation relations or the Poisson-bracket relations (see SII.1.2) of $\beta_{\nu k,\mu}$ and $\beta^{\dagger}_{\nu k,\mu}$ defined in Eqs. (S73) and (S74).

SII.1.2 Poisson bracket

We note that in the classical description, checking the commutation relation corresponds to checking the Poisson bracket for physical quantities A and B

$$\{A, B\} = \int d\mathbf{r} \left(\frac{\delta A}{\delta\phi(\mathbf{r})}\frac{\delta B}{\delta\pi(\mathbf{r})} - \frac{\delta B}{\delta\phi(\mathbf{r})}\frac{\delta A}{\delta\pi(\mathbf{r})}\right), \quad \text{(S92)}$$

where $\phi(\mathbf{r}) = m_y(\mathbf{r})/\sqrt{\gamma M_\mathrm{s}(\mathbf{r})}$ is the field variable and $\pi(\mathbf{r}) = m_x(\mathbf{r})/\sqrt{\gamma M_\mathrm{s}(\mathbf{r})}$ is the conjugate momentum as in the standard classical field theory. The identification of these variables is made such that we have a correct equation of motion for the magnetization dynamics. Importantly, in terms of the complex canonical variables, we obtain

$$\{A, B\} = -i \int d\mathbf{r} \left(\frac{\delta A}{\delta a(\mathbf{r})} \frac{\delta B}{\delta a^*(\mathbf{r})} - \frac{\delta B}{\delta a(\mathbf{r})} \frac{\delta A}{\delta a^*(\mathbf{r})} \right), \tag{S93}$$

which makes the following Hamiltonian equations of motion for the complex canonical variables more comprehensible

$$\partial_t a(\mathbf{r}) = -i \frac{\delta \mathcal{H}_\mathrm{m}}{\delta a^*(\mathbf{r})} = \{a(\mathbf{r}), \mathcal{H}_\mathrm{m}\}, \tag{S94}$$

$$\partial_t a^*(\mathbf{r}) = +i \frac{\delta \mathcal{H}_\mathrm{m}}{\delta a(\mathbf{r})} = \{a^*(\mathbf{r}), \mathcal{H}_\mathrm{m}\}. \tag{S95}$$

With the definition of the Fourier components of the complex canonical variables presented in Eqs. (S67) and (S68), we obtain

$$\{A, B\} = -i \int dx \sum_{\mathbf{k}} \left(\frac{\delta A}{\delta a_{\mathbf{k}}(x)} \frac{\delta B}{\delta a^*_{\mathbf{k}}(x)} - \frac{\delta B}{\delta a_{\mathbf{k}}(x)} \frac{\delta A}{\delta a^*_{\mathbf{k}}(x)} \right). \tag{S96}$$

Using now the definition of the Bogoliubov transformation Eq. (S71)-(S74), the $\mathbf{k} \parallel \pm\hat{y}$ part of the Poisson bracket is changed and it reads

$$\begin{aligned} \{A, B\} = \quad & -i \int dx \sum_{\mathbf{k} \nparallel \pm\hat{y}} \left(\frac{\delta A}{\delta a_{\mathbf{k}}(x)} \frac{\delta B}{\delta a^*_{\mathbf{k}}(x)} - \frac{\delta B}{\delta a_{\mathbf{k}}(x)} \frac{\delta A}{\delta a^*_{\mathbf{k}}(x)} \right) \\ & -i \sum_{k,\nu,\mu} \left(\frac{\partial A}{\delta \beta_{\nu k,\mu}} \frac{\partial B}{\delta \beta^*_{\nu k,\mu}} - \frac{\partial B}{\delta \beta_{\nu k,\mu}} \frac{\partial A}{\delta \beta^*_{\nu k,\mu}} \right), \end{aligned} \tag{S97}$$

where we used Eqs. (S90) and (S91) to obtain the second term. Recalling the Dirac's quantization rule $i\{A, B\} \to [A, B]$, the correspondence between the classical complex canonical variables with $i\{a(\mathbf{r}), a^*(\mathbf{r}')\} = \delta(\mathbf{r} - \mathbf{r}')$ and the quantum creation and annihilation operators with $[a(\mathbf{r}), a^\dagger(\mathbf{r}')] = \delta(\mathbf{r} - \mathbf{r}')$ is clear. The normalization of the basis functions of the Bogoliubov transformation is in fact determined such that the Poisson bracket takes an appropriate form $i\{\beta_{\nu k,\mu}, \beta^*_{\nu' k',\mu'}\} = \delta_{k,k'}\delta_{\nu,\nu'}\delta_{\mu,\mu'}$.

SII.1.3 Inner product

Based on Ref. (*28*), to obtain an orthonormal basis from the positive-frequency solutions $\mathcal{Y}_\mu(\mathbf{r}) = (\phi_\mu(\mathbf{r}), \pi_\mu(\mathbf{r}))$ for general harmonic-oscillator problems [i.e. solutions proportional to $\exp(-i\omega_\mu t)$], we need to normalize the solutions of the Hamiltonian equations of motion following

$$((\mathcal{Y}_{\mu_1}, \mathcal{Y}_{\mu_2})) = \delta_{\mu_1,\mu_2}, \tag{S98}$$

where the map $((\cdots,\cdots)) : \mathcal{S}^{\mathbb{C}} \times \mathcal{S}^{\mathbb{C}} \to \mathbb{C}$ (where $\mathcal{S}^{\mathbb{C}}$ is a vector space of solutions in $\mathbb{C}$) is defined by

$$((\mathcal{Y}_{\mu_1}, \mathcal{Y}_{\mu_2})) \equiv -i\Omega(\bar{\mathcal{Y}}_{\mu_1}, \mathcal{Y}_{\mu_2}) \equiv -i \int d\mathbf{r} \left(\bar{\phi}_{\mu_1}(\mathbf{r})\pi_{\mu_2}(\mathbf{r}) - \bar{\pi}_{\mu_1}(\mathbf{r})\phi_{\mu_2}(\mathbf{r})\right). \tag{S99}$$

Note that this map satisfies all the properties of an inner product on $\mathcal{S}^{\mathbb{C}}$, except that it fails to be positive definite (*28*). However, when applied to the positive-frequency solutions, it is positive definite, and hence it becomes an inner product. Here, $\Omega(\cdots,\cdots)$ is the symplectic structure (*28*) and $\overline{\cdots}$ indicates the complex conjugation in this subsection to avoid confusion with the asterisk in the complex canonical variables. For the magnon case, recalling the identification $\phi(\mathbf{r}) = m^y(\mathbf{r})/\sqrt{\gamma M_s(\mathbf{r})}$ and $\pi(\mathbf{r}) = m^x(\mathbf{r})/\sqrt{\gamma M_s(\mathbf{r})}$, this becomes

$$\delta_{\mu_1,\mu_2} = ((\mathcal{Y}_{\mu_1}, \mathcal{Y}_{\mu_2})) \equiv -i \int d\mathbf{r} \left(\bar{m}^y_{\mu_1}(\mathbf{r})m^x_{\mu_2}(\mathbf{r}) - \bar{m}^x_{\mu_1}(\mathbf{r})m^y_{\mu_2}(\mathbf{r})\right)/\gamma M_{\mathrm{s}}(\mathbf{r}). \tag{S100}$$

Now, the oscillating solutions in space with the wave vector $\mathbf{k} = k_y\hat{y} + k_z\hat{z}$ can be written as

$$m^x_{\mathbf{k},\mu}(\mathbf{r}) = \frac{\mathrm{e}^{i\mathbf{k}\cdot\boldsymbol{\rho}}}{\sqrt{L^2}} m^x_{\mathbf{k},\mu}(x); \quad m^y_{\mathbf{k},\mu}(\mathbf{r}) = \frac{\mathrm{e}^{i\mathbf{k}\cdot\boldsymbol{\rho}}}{\sqrt{L^2}} m^y_{\mathbf{k},\mu}(x), \tag{S101}$$

where we replaced the solution label $\mu \to (\mathbf{k}, \mu)$. Substituting Eq. (S101) into Eq. (S100), we obtain

$$\begin{aligned}
\delta_{\mu_1,\mu_2} = ((\mathcal{Y}_{\mathbf{k},\mu_1}, \mathcal{Y}_{\mathbf{k},\mu_2})) &= -i \int dx \left(\bar{m}^y_{\mathbf{k},\mu_1}(x)m^x_{\mathbf{k},\mu_2}(x) - \bar{m}^x_{\mathbf{k},\mu_1}(x)m^y_{\mathbf{k},\mu_2}(x)\right)/\gamma M_{\mathrm{s}}(x), \\
&= \int dx \left(\bar{m}^-_{\mathbf{k},\mu_1}(x)m^-_{\mathbf{k},\mu_2}(x) - \bar{m}^+_{\mathbf{k},\mu_1}(x)m^+_{\mathbf{k},\mu_2}(x)\right)/2\gamma M_{\mathrm{s}}(x), \\
&= \int dx \left(\bar{a}_{\mathbf{k},\mu_1}(x)a_{\mathbf{k},\mu_2}(x) - \bar{a}^*_{\mathbf{k},\mu_1}(x)a^*_{\mathbf{k},\mu_2}(x)\right),
\end{aligned} \tag{S102}$$

which is also presented in Eq. (S75). Then, the normalization condition is given by

$$1 = ((\mathcal{Y}_{\mathbf{k},\mu}, \mathcal{Y}_{\mathbf{k},\mu})) = \int dx (|a_{\mathbf{k},\mu}(x)|^2 - |a^*_{\mathbf{k},\mu}(x)|^2). \tag{S103}$$

We note that $a^*_{\mathbf{k},\mu}(x)$ is not the complex conjugation of $a_{\mathbf{k},\mu}(x)$. For example, the positive-frequency solutions in the momentum $\mathbf{k} = \nu k\hat{y}$ are given by the combination of $a_{\nu k\hat{y},\mu}(x) = \xi^{(\mu)}_{\nu k}(x)$ and $a^*_{\nu k\hat{y},\mu} = \eta^{(\mu)}_{\nu k}(x)$. Under this identification, the orthogonality relation Eq. (S102) becomes Eq. (S90). In fact, the normalization of the positive-frequency solutions with the inner product defined in Eq. (S99) is a part of the general approach of quantizing a collection of classical Harmonic oscillators (*28*).

SII.1.4 Hamiltonian equations of motion for MSSW

In this section, we show that the Hamiltonian equations of motion governed by the magnon Hamiltonian Eq. (S64) automatically derives the Maxwell's equations and their corresponding boundary conditions. Conceptually, this is because the spin-wave dynamics is described by the coupled equations of (i) the magnetization precession equation [i.e. $\partial_t \mathbf{M}(t) = \gamma\mu_0 \mathbf{H} \times \mathbf{M}$ for the magnetization $\mathbf{M}$ and the magnetic field $\mathbf{H}$] and (ii) the magnetostatic Maxwell equations [i.e. the Maxwell's equations with $\nabla \times \mathbf{H} \approx 0$]. One way to solve them is to solve the former equations [i.e. (i)] first using the Polder tensor and then substitute the solution into (ii), which makes the problem into the magetostatics problem called the Walker's equation (*27*). An alternative approach is to solve the latter equations [i.e. (ii)] first using the magnetostatic Green's function and then substitute the solution into (ii). Hamiltonian equation of motion derived from the magnon Hamiltonian Eq. (S64) provides the equation obtained from the latter approach.

In the following, we do not assume the Maxwell's equation with a priori. We only use the constraint that the magnetization is zero outside the magnet. The Hamiltonian equations of motion derived from Eq. (S64) is

$$\partial_t \mathbf{m}_\mathbf{k}(x) = \hat{z} \times \left[\omega_H \mathbf{m}_\mathbf{k}(x) - \omega_M \int dx' \hat{D}_\mathbf{k}(x-x') \cdot \mathbf{m}_\mathbf{k}(x')\right]. \tag{S104}$$

Now, we define a thee-dimensional vector $\mathbf{h}_\mathbf{k}(x)$ (which turns out to be a magnetic field later) by

$$\begin{aligned} \mathbf{h}_\mathbf{k}(x) &\equiv \int dx' \hat{D}_\mathbf{k}(x-x') \cdot \mathbf{m}_\mathbf{k}(x'), \\ &= \int dx' \left[\frac{ke^{-k|x-x'|}}{2} \hat{\sigma}_\mathbf{k}^{-\mathrm{sgn}(x-x')} \otimes \hat{\sigma}_\mathbf{k}^{-\mathrm{sgn}(x-x')} - \hat{x} \otimes \hat{x}\delta(x-x')\right] \cdot \mathbf{m}_\mathbf{k}(x'), \end{aligned} \tag{S105}$$

where we used Eq. (S65) for the explicit form of $\hat{D}_\mathbf{k}(x-x')$. We note that $\mathbf{h}_\mathbf{k}(x)$ has a z component

unlike $\mathbf{m_k}(x)$, which is defined to be a two-dimensional vector in the x-y plane. Then, Eq. (S104) for positive-frequency solutions with frequency ω becomes

$$-i\omega\mathbf{m_k}(x) = \hat{z} \times [\omega_H \mathbf{m_k} - \mathbf{h_k}(x)]. \tag{S106}$$

Applying $\hat{z}\times$ on both-hand sides and rearranging terms, we obtain

$$\mathbb{P}\mathbf{h_k}(x) = \frac{1}{\omega_M}(\omega_H \mathbf{m_k}(x) - i\omega\hat{z} \times \mathbf{m_k}(x)) = \frac{\omega_H - i\omega(\hat{y}\otimes\hat{x} - \hat{x}\otimes\hat{y})}{\omega_M} \cdot \mathbf{m_k}(x), \tag{S107}$$

where $\mathbb{P} = \mathbb{I} - \hat{z}\otimes\hat{z}$ represents the projection onto the x-y plane. Inverting this equation, we obtain

$$\mathbf{m_k}(x) = \frac{\omega_H\omega_M + i\omega\omega_M(\hat{y}\otimes\hat{x} - \hat{x}\otimes\hat{y})}{\omega_H^2 - \omega^2} \cdot \mathbb{P}\mathbf{h_k}(x) \equiv \bar{\boldsymbol{\chi}} \cdot \mathbf{h_k}(x), \tag{S108}$$

where we defined the Polder susceptibility tensor $\bar{\boldsymbol{\chi}}$ by

$$\bar{\boldsymbol{\chi}} \equiv \frac{\omega_H\omega_M + i\omega\omega_M(\hat{y}\otimes\hat{x} - \hat{x}\otimes\hat{y})}{\omega_H^2 - \omega^2} \cdot \mathbb{P} = [\hat{x} \quad \hat{y}] \begin{bmatrix} \chi & -i\kappa \\ \kappa & \chi \end{bmatrix} \begin{bmatrix} \hat{x} \\ \hat{y} \end{bmatrix}, \tag{S109}$$

with $\chi = \omega_H\omega_M/(\omega_H^2 - \omega^2)$ and $\kappa = \omega\omega_M/(\omega_H^2 - \omega^2)$.

Now, we derive the magnetostatic Maxwell's equations from Eq. (S105). Integrating by parts using the condition that the Fourier representation of the magnetization $\mathbf{m_k}(x)$ is zero at $x \to \pm\infty$, it can be rewritten as

$$\mathbf{h_k}(x) = (\hat{x}\partial_x + i\mathbf{k}) \int dx' G_\mathbf{k}(x - x')(\hat{x}\partial'_x + i\mathbf{k}) \cdot \mathbf{m_k}(x'), \tag{S110}$$

where we defined the Fourier representation of the Green's function for the (negative of the) Laplace equation

$$G_\mathbf{k}(x - x') \equiv \frac{1}{2k} e^{-|x-x'|}; \quad (\partial_x^2 - k^2) G_\mathbf{k}(x - x') = -\delta(x - x'). \tag{S111}$$

Applying the rotation $[(\hat{x}\partial_x + i\mathbf{k})\times]$ and the divergence $[(\hat{x}\partial_x + i\mathbf{k})\cdot]$ to the both-hand sides of Eq. (S110), and using the Green's function's property Eq. (S111), we obtain

$$(\hat{x}\partial_x + i\mathbf{k}) \times \mathbf{h_k}(x) = 0, \tag{S112}$$

$$(\hat{x}\partial_x + i\mathbf{k}) \cdot \mathbf{h_k}(x) = -(\hat{x}\partial_x + i\mathbf{k}) \cdot \mathbf{m_k}(x), \tag{S113}$$

where the first line is the Ampere's law under the magnetostatic approximation, and the second line is the divergence-free condition for the Fourier representation of the magnetic-flux density defined by

$$\mathbf{b}_{\mathbf{k}}(x) \equiv \mu_0[\mathbf{h}_{\mathbf{k}}(x) + \mathbf{m}_{\mathbf{k}}(x)], \tag{S114}$$

which turns Eq. (S113) into a more apparent expression of the Gauss's law for the magnetic-flux density

$$(\hat{x}\partial_x + i\mathbf{k}) \cdot \mathbf{b}_{\mathbf{k}}(x) = 0. \tag{S115}$$

We note that the component-wise computation of Eq. (S112) results in

$$\mathbf{h}_{\mathbf{k}}(x) = -(\hat{x}\partial_x + i\mathbf{k})\phi_{\mathbf{k},\mathrm{m}}(x), \tag{S116}$$

where $\phi_{\mathbf{k},\mathrm{m}}(x)$ is a Fourier representation of the magnetic potential [which is defined by $\phi_{\mathbf{k},\mathrm{m}}(x) = i\mathbf{k} \cdot \mathbf{h}_{\mathbf{k}}(x)/k^2$ in the current setting]. Combining Eqs. (S108) and (S114)- (S116), we obtain the Walker's equation

$$(\hat{x}\partial_x + i\mathbf{k}) \cdot (\mathbb{I} + \bar{\boldsymbol{\chi}}) \cdot (\hat{x}\partial_x + i\mathbf{k})\phi_{\mathbf{k},\mathrm{m}}(x) = 0, \tag{S117}$$

where $\mathbb{I}$ is an identity operator $\mathbb{I} = \hat{x} \otimes \hat{x} + \hat{y} \otimes \hat{y} + \hat{z} \otimes \hat{z}$.

The Maxwell's equation's boundary conditions are also derived from the definition of the Fourier representation of the magnetic field given by Eq. (S105). Firstly, using the condition that the Fourier representation of the magnetization $\mathbf{m}_{\mathbf{k}}(x)$ is zero outside the ferromagnet (i.e. $x > d/2$ or $-d/2 > x$), we obtain from Eq. (S105) and Eq. (S114)

$$\lim_{x\to\pm\infty} \mathbf{h}_{\mathbf{k}}(x) = \lim_{x\to\pm\infty} \mathbf{b}_{\mathbf{k}}(x) = 0. \tag{S118}$$

Next, for the boundary conditions at $x = \pm d/2$, we compute the difference of Eq. (S105) evaluated at $x = x_{\mathrm{out}} \equiv \pm(d/2 + \epsilon)$ and $x = x_{\mathrm{in}} \equiv \pm(d/2 - \epsilon)$ for a small $\epsilon \to 0$. Then, we obtain

$$\mathbf{h}_{\mathbf{k}}(x_{\mathrm{out}}) - \mathbf{h}_{\mathbf{k}}(x_{\mathrm{in}}) = \int dx' \, [\hat{x} \otimes \hat{x}\delta(x_{\mathrm{in}} - x')] \cdot \mathbf{m}_{\mathbf{k}}(x') = \hat{x}[\hat{x} \cdot \mathbf{m}_{\mathbf{k}}(x_{\mathrm{in}})], \tag{S119}$$

where the contribution from $\delta(x_{\mathrm{out}} - x')$ is zero as the integration over x' is performed after multiplied with $\mathbf{m}_{\mathbf{k}}(x')$ which is zero outside the ferromagnet. Also, the integration of the term with $e^{-|x-x'|}$

approaches to zero when $\epsilon \to 0$. Recalling the definition of $\mathbf{b}_{\mathbf{k}}(x)$ in Eq. (S114) and $\mathbf{m}_{\mathbf{k}}(x_{\rm out}) = 0$, we find that Eq. (S119) is the standard boundary conditions for the Maxwell's equations, i.e.

$$\hat{x} \times \mathbf{h}_{\mathbf{k}}(x_{\rm out}) = \hat{x} \times \mathbf{h}_{\mathbf{k}}(x_{\rm in}), \tag{S120}$$

$$\hat{x} \cdot \mathbf{b}_{\mathbf{k}}(x_{\rm out}) = \hat{x} \cdot \mathbf{b}_{\mathbf{k}}(x_{\rm in}), \tag{S121}$$

where $\pm\hat{x}$ is the unit normal vector at the ferromagnet's surface $x = \pm d/2$.

SII.1.5 Orthonormality of functions provided in Eqs. (S87)-(S89)

The orthonormality of the functions provided in Eqs. (S87)-(S89) can be verified by noticing that they are the eigenfunctions of ∂_x^2 in the range $d/2 \geq x \geq -d/2$ under the following boundary conditions. They are the complete set of solutions of

$$\begin{cases} \partial_x^2 F(x) = \lambda F(x); \quad (d/2 \geq x \geq -d/2), \\ \partial_x F(x)|_{x=\pm d/2} = \nu k F(x)|_{x=\pm d/2}\,. \end{cases} \tag{S122}$$

However, we should note that we did not impose any extra boundary conditions to obtain these functions from the Hamitonian equations of motion. In fact, the orthonormality Eqs. (S90) and (S91) can be derived from the property of the solutions to the Hamiltoian equations of motion together with the inner product [or simply the map, as discussed in Ref. (*28*) and Sec. SII.1.3] defined in Eq. (S75), instead of using the orthonormality of the functions in Eqs. (S87)-(S89).

SII.1.6 Magnon-mode functions with mode labels $\mu = 1, 2, \cdots$

The magnon-mode functions with mode labels $\mu = 1, 2, \cdots$ in Eqs. (S79) and (S80) are derived in the following way. We would like to obtain the complete set of solutions of the Walker's equation Eq. (S117) for the momentum sector $\mathbf{k} = \mu k\hat{y}$

$$(\partial_x^2 - k^2)\phi^{\rm I}_{\nu k\hat{y},{\rm m}}(x) = 0; \quad (\text{Region II}\,, x > d/2), \tag{S123}$$

$$(1+\chi)(\partial_x^2 - k^2)\phi^{\rm II}_{\nu k\hat{y},{\rm m}}(x) = 0; \quad (\text{Region II}, |x| \leq d/2), \tag{S124}$$

$$(\partial_x^2 - k^2)\phi^{\rm III}_{\nu k\hat{y},{\rm m}}(x) = 0; \quad (\text{Region III}, x < -d/2). \tag{S125}$$

The solution for $\chi \neq -1$ is well studied, and known to show the MSSW dispersion (*27*). By normalizing the solution following Sec. SII.1.3, we obtain the mode functions with the mode label $\mu = 0$. As

we need a complete set of solutions to perform the Bogoliubov transformation, in the following we show the solution for $\chi = -1$. In this case, Eq. (S124) is satisfied for any functions $\phi^{\rm II}_{\nu k\hat{y},{\rm m}}(x) \equiv F(x)$. Therefore, using the boundary conditions at $x \to \pm\infty$, we write the magnetic potential for each region as

$$\phi^{\rm I}_{\nu k\hat{y},{\rm m}}(x) = Ce^{-k(x-d/2)}, \tag{S126}$$

$$\phi^{\rm II}_{\nu k\hat{y},{\rm m}}(x) = F(x), \tag{S127}$$

$$\phi^{\rm III}_{\nu k\hat{y},{\rm m}}(x) = De^{+k(x+d/2)}. \tag{S128}$$

From the boundary conditions at $x = \pm d/2$ [i.e. Eqs. (S120) and (S121)], we obtain $C = F(d/2) = -\kappa\nu F(d/2)$ and $D = F(-d/2) = \kappa\nu F(-d/2)$. This results in $C = D = 0$, and $F(x)$ is any smooth functions with

$$F(d/2) = F(-d/2) = 0. \tag{S129}$$

Therefore, a complete set of solutions of the magnetic potential for the case $\chi = -1$ is the one provided in Eq. (S83). By normalizing the positive-frequency solutions obtained by this procedure following Sec. SII.1.3, we obtain the magnon-mode functions Eqs. (S79) and (S80).

SII.2 NV-magnon coupling

Here we compute the interaction Hamiltonian between the NV center and magnons. This interaction is due to the magnetic dipole-dipole interaction between the NV-center's spin $\mathbf{S}_{\rm NV}$ and the magnetization $\mathbf{M}(\mathbf{r})$, which is equal to the Zeeman interaction between the NV-center's spin and the dipole field $\mathbf{h}_{\rm d}(\mathbf{r}_0)$ generated by magnons at the position of the NV center $\mathbf{r}_0 = \boldsymbol{\rho}_0 + x_0\hat{x}$ (with $x_0 > d/2$). As we consider the case where the NV center is located well outside the YIG, the wave function of the electrons in YIG do not overlap with the wave functions of the electrons constituting the NV center levels. Acordingly, we do not consider the exchange interaction between the YIG spins and the NV spin as this is negligible. The interaction Hamiltonian reads

$$\mathcal{H}_{\rm int} = \gamma\mu_0\mathbf{S}_{\rm NV}\cdot\mathbf{h}_{\rm d}(\mathbf{r}_0), \tag{S130}$$

where

$$\mathbf{h}_{\rm d}(\mathbf{r}_0) = \nabla \int d\mathbf{r}' G(\mathbf{r}-\mathbf{r}')\nabla' \cdot \mathbf{M}(\mathbf{r}')\Big|_{\mathbf{r}=\mathbf{r}_0} = \sum_{\mathbf{k}} \frac{e^{i\mathbf{k}\cdot\boldsymbol{\rho}_0}}{\sqrt{L^2}} \mathbf{h}_{\rm d,\mathbf{k}}(x_0), \tag{S131}$$

$$\mathbf{h}_{\rm d,\mathbf{k}}(x) = \int dx' \hat{D}_{\mathbf{k}}(x-x') \cdot \mathbf{M}_{\mathbf{k}}(x'), \tag{S132}$$

$$\mathbf{M}(\mathbf{r}) = \sum_{\mathbf{k}} \frac{e^{i\mathbf{k}\cdot\boldsymbol{\rho}}}{\sqrt{L^2}} \mathbf{M}_{\mathbf{k}}(x); \quad \mathbf{M}_{\mathbf{k}}(x) \approx \mathbf{m}_{\mathbf{k}}(x) + M_{\rm s}(x)\sqrt{L^2}\delta_{\mathbf{k},\mathbf{0}}\hat{z}. \tag{S133}$$

In Eq. (S133), the approximation means that the equality is accurate up to linear order in the complex canonical variables. It is worth noting that the static magnetic field generated by the magnetization is zero [i.e. the contribution of the static magnetization $M_{\rm s}(x)\sqrt{L^2}\delta_{\mathbf{k},\mathbf{0}}\hat{z}$ in Eq. (S133) on the dipole field $\mathbf{h}_{\rm d,\mathbf{k}}(x)$ is zero] after a direct computation for the geometry under consideration. We focus on the magnetic field with Fourier components $\mathbf{k} \parallel \pm\hat{y}$, as our interest is the MSSW modes, which are the modes propagating along $\pm\hat{y}$ direction, and therefore the Fourier domain magnetic fields with $\mathbf{k} \parallel \pm\hat{y}$ are the only terms that will contain $\beta_{\nu k,\mu}$ and $\beta^*_{\nu k,\mu}$. Up to quadratic order in the complex canonical variables, the Fourier components of the dipole field reads

$$h^-_{{\rm d},\nu k\hat{y}}(x_0) = (\mathcal{A}_{-k}\beta_{-k,0} + \mathcal{A}_{+k}\beta^*_{+k,0})\delta_{\nu,-1}, \tag{S134}$$

$$h^+_{{\rm d},\nu k\hat{y}}(x_0) = (\mathcal{A}_{+k}\beta_{+k,0} + \mathcal{A}_{-k}\beta^*_{-k,0})\delta_{\nu,+1}, \tag{S135}$$

$$h^z_{{\rm d},\nu k\hat{y}}(x_0) = 0. \tag{S136}$$

where $h^{\mp}_{\rm d,\mathbf{k}}(x_0) = h^x_{\rm d,\mathbf{k}}(x_0) \mp i h^y_{\rm d,\mathbf{k}}(x_0)$ and we defined

$$\mathcal{A}_{\nu k} = \sqrt{2k\gamma M_{\rm s}}\mathcal{M}_{\nu k}e^{-kh_0}, \tag{S137}$$

$$\mathcal{M}_{\nu k} = \nu\sqrt{(e^{-kd}\sinh kd)\,(\chi_0 + 2 - \nu\kappa_0)(\chi_0 + \nu\kappa_0)/4\kappa_0}. \tag{S138}$$

Here, $h_0 = x_0 - d/2$ is the distance between the NV and the top YIG surface. Spin waves with $\mu \neq 0$ do not generate magnetic field outside the ferromagnetic film and hence does not contribute to the interaction, which can be verified by an explicit calculation. Note that $\mathcal{M}_{-k}$ is smaller than $\mathcal{M}_{+k}$ due to the chirality of the magnetic field generated by the magnetization texture. In the long-wavelength

limit ($k \to 0$) and the short-wavelength limit ($k \to \infty$ or $k \gg 1/d$), we obtain

$$\mathcal{M}_{\nu k} \to (1/2)(\sqrt{\omega_H/\omega_{\rm K}} + \nu\sqrt{\omega_{\rm K}/\omega_H})\sqrt{kd} \approx 0, (k \to 0), \tag{S139}$$

$$\mathcal{M}_{+k} \to 1/\sqrt{2}, (k \to \infty), \tag{S140}$$

$$\mathcal{M}_{-k} \to -(\omega_M/(2\omega_H + \omega_M))e^{-kd}/2\sqrt{2} \approx 0, (k \to \infty), \tag{S141}$$

where $\omega_{\rm K} = \lim_{k\to 0}\omega_0(k) = \sqrt{\omega_H(\omega_H + \omega_M)}$ is the Kittel frequency. The Eqs. (S134) and (S135) are linear in the complex canonical variables because we have dropped higher order terms in the Holstein-Primakoff transformation defined by Eqs. (S171)-(S173). In contrast, Eq. (S136) is zero for all orders in the complex canonical variables because $\hat{D}_{\nu k\hat{y}}(x_0 - x') \cdot \mathbf{M}_{\nu k\hat{y}}(x') \propto (\hat{x} - i\nu\hat{y})$ in Eq. (S132) does not contain the z component.

Substituting Eqs. (S134)-(S136) and (S131) into Eq. (S130), we obtain the NV-MSSW interaction Hamiltonian:

$$\begin{aligned} \mathcal{H}_{\rm int}^{\mathbf{k}\|\pm\hat{y}} &= \sum_{\mathbf{k}\|\pm\hat{y}} \frac{e^{i\mathbf{k}\cdot\boldsymbol{\rho}_0}}{\sqrt{L^2}}\gamma\mu_0\mathbf{S}_{\rm NV}\cdot\mathbf{h}_{\rm d,\mathbf{k}}(x_0), \\ &= \frac{\gamma\mu_0}{2L}\sum_{k\geq 0}[S_{\rm NV}^-(\mathcal{A}_{+k}\beta_{+k,0} + \mathcal{A}_{-k}\beta_{-k,0}^\dagger)e^{iky_0} + {\rm H.c.}], \end{aligned} \tag{S142}$$

which is shown in Eq. (S55) at the beginning of Section SII.

SII.2.1 NV-MSSW coupling constant

When the NV center's main symmetry axis is along the $\hat{z}$ direction and the intensity of the external magnetic field is smaller than NV center's zero-field splitting (by converting the unit by the absolute value of the electron gyromagnetic ratio γ), the ground state and the first excited state of the NV center is $|g\rangle = |m_s = 0\rangle$ and $|e\rangle = |m_s = -1\rangle$, respectively (see Sec. SIII). Under the rotating-wave approximation within the Hilbert space spanned by these two levels, we obtain the interaction Hamiltonian $\mathcal{H}_{\rm int}^{\mathbf{k}\|\pm\hat{y}}$ and the NV-MSSW coupling $g_k^{\rm (MSSW)}$ as

$$\mathcal{H}_{\rm int}^{\mathbf{k}\|\pm\hat{y}} = \sum_{k\geq 0}[g_k^{\rm (MSSW)}\beta_{+k,0}e^{iky_0}\sigma_+ + {\rm H.c.}]; \quad g_k^{\rm (MSSW)} = \frac{\gamma\mu_0}{\sqrt{2}L}\mathcal{A}_{+k}, \tag{S143}$$

where $\sigma_+ = |e\rangle\langle g| \approx S_{\rm NV}^-/\sqrt{2}$. It is worth noting that the coupling $g_k^{\rm (MSSW)}$ is independent of the external magnetic field in the limit $k \gg 1/d$, because we obtain $\mathcal{A}_{+k} \approx \sqrt{k\gamma M_{\rm s}}e^{-kh_0}$ using

Eqs. (S137) and (S140), which results in

$$g_k^{(\mathrm{MSSW})} \approx \frac{\gamma\mu_0}{\sqrt{2}L}\sqrt{k\gamma M_{\mathrm{s}}}e^{-kh_0}, (k \gg 1/d), \tag{S144}$$

$$\omega_0(k) \approx \omega_H + \omega_M/2, (k \gg 1/d), \tag{S145}$$

where we used Eq. (S77) for the second equation. Notably, the wavenumber (k) dependence of the NV-MSSW coupling is $g_k^{(\mathrm{MSSW})} \propto \sqrt{k}e^{-kh_0}$ based on Eq. (S144), instead of $\propto ke^{-kh}$, which may be anticipated from the dipole tensor Eq. (S65) applied to Eq. (S132). The presence of the factor $\sqrt{k}$ instead of k is a consequence of the integration over x' in Eq. (S132) (adding a factor $\propto 1/k$), as well as the normalization ($\propto \sqrt{k}$) of the exponential mode function in Eq. (S89). More concretely, in the large wavenumber limit, we obtain the MSSW mode function $m^-_{+k\hat{y}}(x) \propto \xi^{(0)}_{+k}(x) \underset{\sim}{\propto} \sqrt{k/e^{kd}}e^{kx}$. Using this expression, we find that Eq. (S132) results in $h^+_{\mathrm{d},+k\hat{y}}(x = h_0 + d/2) \underset{\sim}{\propto} \int_{-d/2}^{d/2} dxke^{-k(h_0+d/2-x)}\sqrt{k/e^{kd}}e^{+kx} \underset{\sim}{\propto} \sqrt{k}e^{-kh_0}$. This computation validates the wavenumber dependence of the NV-MSSW coupling shown in Eq. (S144).

SII.3 Magnon-MSL coupling

In this section, we compute the interaction Hamiltonian between the magnons and MSL fabricated on the YIG film. We model the current density as $j = j(x, y) = (I/w)\Theta(y+w/2)\Theta(w/2-y)\delta(x-d/2)$, where I is the net current, w is the width of the MSL, and $\Theta(\cdot)$ is the Heaviside step function. The magnetic field generated by the MSL arises from the Biot-Savart law and reads

$$\mathbf{H}_{\mathrm{MSL}}(x, y) = \frac{I}{2}\sum_{\nu=\pm 1}\int_0^\infty \frac{dk}{2\pi}\frac{\sin(kw/2)}{kw/2} i\nu\hat{\sigma}_{\nu k\hat{y}}^{-\mathrm{sgn}(x-d/2)} e^{i\nu ky - k|x-d/2|}. \tag{S146}$$

One obtains the interaction Hamiltonian between MSL and magnons by calculating

$$\begin{aligned}\mathcal{H}_{\mathrm{MSL}} &= -\mu_0 \int d\mathbf{r}\mathbf{H}_{\mathrm{MSL}}(x, y)\cdot\mathbf{M}(\mathbf{r}), \\ &= -\sum_{\nu,k} i[\mathcal{F}_{\nu k}(\beta_{\nu k,0} - \beta^\dagger_{\nu k,0})]I \equiv -\hat{A}I,\end{aligned} \tag{S147}$$

which is presented in Eq. (S56). Here, in the second line we approximated the terms up to linear order in the creation and annihilation operators, and we defined

$$\mathcal{F}_{\nu k} = -\frac{\nu\mu_0}{2}\sqrt{2\gamma M_s/k}\frac{\sin(kw/2)}{kw/2}\mathcal{M}_{\nu k}, \tag{S148}$$

$$\hat{A} = \sum_{\nu,k} i[\mathcal{F}_{\nu k}(\beta_{\nu k,0} - \beta^{\dagger}_{\nu k,0})], \tag{S149}$$

where $\mathcal{M}_{\nu k}$ is defined in Eq. (S138). We note that we did not exclude by hand the summation over $\mu \neq 0$. The contribution of $\mu \neq 0$ modes automatically vanishes after a direct computation. This makes sense as the MSL field $\mathbf{H}_{\mathrm{MSL}}(x, y)$ decays exponentially along the x direction in the YIG and hence it only couples to the MSSW mode, as the modes with $\mu \neq 0$ have profiles which are orthogonal to the exponential function. It also makes sense as the modes with $\mu \neq 0$ do not produce magnetic field outside the YIG. For more detailed explanation on the relation between the absence of magnetic field produced by the $\mu \neq 0$ modes and the consequent absence of interaction between the MSL and these magnon modes, see the next sub-subsection.

SII.3.1 Alternative expression of the magnon-MSL interaction

An intuition into the relationship between the absence of magnetic field generated by $\mu \neq 0$ magnon modes and no magnon-MSL interaction is obtained by considering the interaction between the magnetization and a more generalizable MSL current density $\mathbf{j}_{\mathrm{MSL}}(\mathbf{r}) = \int ds I_{\mathrm{MSL}}\delta(\mathbf{r} - \mathbf{r}_{\mathrm{MSL}}(s))\hat{t}(s)$, where $\mathbf{r}_{\mathrm{MSL}}(s)$ is the MSL position, s parametrizes the position of the MSL, and $\hat{t}(s)$ is the unit tangent vector along the MSL. The magnon-MSL interaction Hamiltonian Eq. (S147) can be firstly rewritten as

$$\begin{aligned}\mathcal{H}_{\mathrm{MSL}} &= -\mu_0 \int d\mathbf{r}\left(\nabla \times \int d\mathbf{r}' G(\mathbf{r}-\mathbf{r}')\mathbf{j}_{\mathrm{MSL}}(\mathbf{r}')\right)\cdot \mathbf{M}(\mathbf{r}) \\ &= -\mu_0 \int d\mathbf{r} d\mathbf{r}' G(\mathbf{r}-\mathbf{r}')\mathbf{j}_{\mathrm{MSL}}(\mathbf{r}')\cdot \mathbf{j}_m(\mathbf{r}), \\ &= -\mu_0 I_{\mathrm{MSL}} \int_{\mathrm{MSL}} d\vec{s}\cdot \int d\mathbf{r}\frac{\nabla \times \mathbf{M}(\mathbf{r})}{4\pi|\mathbf{r}-\mathbf{r}_{\mathrm{MSL}}(s)|},\end{aligned} \tag{S150}$$

where the first line is the integral expression of the Biot–Savart law and in the second line we used integration by parts. Here, $\mathbf{j}_m \equiv \nabla \times \mathbf{M}(\mathbf{r})$ is magnetization's equivalent current density,

and $\int_{\text{MSL}} d\vec{s} \equiv \int ds\hat{t}(s)$ indicates the line integral along the MSL. As shown in the second line of Eq. (S150), the role of the magnetization and the MSL current is symmetric. Assuming that the MSL wire is enclosed (as is always the case when using MSL as a microwave transducer to launch magnons) while the part not directly fabricated on top of the YIG film does not contribute to the interaction Hamiltonian, we can rewrite

$$\begin{aligned}
\mathcal{H}_{\text{MSL}} &\approx -\mu_0 I_{\text{MSL}} \oint_{\text{MSL}} d\vec{s}\cdot \int d\mathbf{r} \frac{\nabla\times\mathbf{M}(\mathbf{r})}{4\pi|\mathbf{r}-\mathbf{r}_{\text{MSL}}(s)|}, \\
&= -\mu_0 I_{\text{MSL}} \int_{\text{MSL}} dS'\hat{n}'\cdot\nabla'\times\int d\mathbf{r}\frac{\nabla\times\mathbf{M}(\mathbf{r})}{4\pi|\mathbf{r}-\mathbf{r}'|}, \\
&= -\mu_0 I_{\text{MSL}} \int_{\text{MSL}} dS'\hat{n}'\cdot\left(\nabla'\int d\mathbf{r}\frac{\nabla\cdot\mathbf{M}(\mathbf{r})}{4\pi|\mathbf{r}-\mathbf{r}'|}+\mathbf{M}(\mathbf{r}')\right), \\
&= -\mu_0 I_{\text{MSL}} \int_{\text{MSL}} dS'\hat{n}'\cdot\int d\mathbf{r}\hat{D}(\mathbf{r}'-\mathbf{r})\cdot\mathbf{M}(\mathbf{r}) = -I_{\text{MSL}}\Phi[\mathbf{M}], \qquad \text{(S151)}
\end{aligned}$$

where in the first line we approximated the integral into the closed integral, and in the second line we applied the Stokes' theorem where $\hat{n}'$ is the normal unit vector and $\int_{\text{MSL}} dS'$ is the surface integral on the surface enclosed by the MSL. Additionally, in the third line we used multiple integration by parts and the property of the Green's function $\nabla^2 G(\mathbf{r}-\mathbf{r}') = -\delta(\mathbf{r}-\mathbf{r}')$. Finally, in the last line we assumed that the magnetization is absent on the surface S' enclosed by the MSL [i.e. $\int_{\text{MSL}} dS'\hat{n}'\cdot\mathbf{M}(\mathbf{r}') = 0$] and used the integration by parts. We defined the flux $\Phi[\mathbf{M}]$ of the magnetic field generated by the magnetization $\mathbf{M}(\mathbf{r})$ that penetrate through the MSL loop via

$$\Phi[\mathbf{M}] = \mu_0 \int_{\text{MSL}} dS'\hat{n}'\cdot\int d\mathbf{r}\hat{D}(\mathbf{r}'-\mathbf{r})\cdot\mathbf{M}(\mathbf{r}), \qquad \text{(S152)}$$

with the dipole tensor $\hat{D}(\mathbf{r}-\mathbf{r}')$ defined by

$$\hat{D}(\mathbf{r}-\mathbf{r}') = -(\nabla\otimes\nabla')G(\mathbf{r}-\mathbf{r}'). \qquad \text{(S153)}$$

Based on the final expression of Eq. (S151), the magnon-MSL interaction Hamiltonian can be understood as the current-flux interaction. Hence, recalling the magnon modes with $\mu\neq 0$ do not generate magnetic field outside the YIG film, there is no flux generated at the MSL loop position and hence the interaction vanishes for these modes.

SII.4 Linear response theory on the magnon-mediated driving of NV centers

Based on the linear response theory or Kubo formula, when we drive the current $I = I_0 e^{-i\omega t}$ with frequency ω, the response of the field $\hat{h}_{\rm d}^+ = \hat{h}_{\rm d}^+(\mathbf{r}_0)$ is given by

$$\delta\langle\hat{h}_{\rm d}^+(t)\rangle = \chi_{h_{\rm d}^+ A}[\omega] I_0 e^{-i\omega t}, \tag{S154}$$

$$\begin{aligned}\chi_{h_{\rm d}^+ A}[\omega] &= i\int_{-\infty}^{\infty} dt e^{i\omega t}\langle[\hat{h}_{\rm d}^+(t), \hat{A}(0)]\rangle_{\rm eq}\Theta(t), \\ &= \int_0^\infty \frac{dk}{2\pi}\left[\mathcal{A}_{+k}\mathcal{F}_{+k}\frac{i}{\omega-\omega_0(k)+i0^+} + \mathcal{A}_{-k}\mathcal{F}_{-k}\frac{i}{\omega+\omega_0(k)+i0^+}\right] e^{iky_0},\end{aligned} \tag{S155}$$

$$\mathcal{A}_{\nu k}\mathcal{F}_{\nu k} = -\nu\omega_M \frac{\sin(kw/2)}{kw/2}\frac{\left(e^{-kd}\sinh kd\right)(\chi_0+2-\nu\kappa_0)(\chi_0+\nu\kappa_0)}{4\kappa_0} e^{-kh_0}, \tag{S156}$$

where $\langle\cdots\rangle_{\rm eq}$ represents the equilibrium average, $\chi_{h_{\rm d}^+ A}[\omega]$ is the susceptibility, and $(\omega \mp \omega_0(k) + i0^+)^{-1}$ is related to the MSSW propagator with infinitesimal $0^+ \to +0$. This result is identical to the classical calculation in Ref. (*25*) and Eq. (S54), except for the infinitesimal $\pm i0^+$ in the pole of Eq. (S155).

SII.4.1 Radiation resistance of the microstrip antenna due to the emission of MSSW

As a sanity check of the Hamiltonian formalism of the MSSW, we show that this formalism can successfully derive the radiation resistance due to the radiation of MSSW presented in various literature (*27*). To this end, we consider applying current $I = I_0(e^{-i\omega_{\rm d}t} + e^{+i\omega_{\rm d}t})\Theta(t)$ in (S147). From the Hamiltonian equations of motion, we obtain

$$\beta_{\nu k,\mu}(t) = e^{-i\omega_\mu t}\beta_{\nu k,\mu}(0) + I_0\mathcal{F}_{\nu k}\delta_{\mu,0}\int_0^t d\tau e^{-i\omega_\mu(t-\tau)}\left(e^{-i\omega_{\rm d}\tau} + e^{+i\omega_{\rm d}\tau}\right), \tag{S157}$$

$$\beta^*_{\nu k,\mu}(t) = e^{+i\omega_\mu t}\beta^*_{\nu k,\mu}(0) + I_0\mathcal{F}_{\nu k}\delta_{\mu,0}\int_0^t d\tau e^{+i\omega_\mu(t-\tau)}\left(e^{-i\omega_{\rm d}\tau} + e^{+i\omega_{\rm d}\tau}\right). \tag{S158}$$

Taking the long time limit $t \to \infty$ and assuming $\beta_{\nu k,\mu}(0) = \beta^*_{\nu k,\mu}(0)$=0, where we do not need to consider the thermal magnon contribution as we are currently working in a regime of classical mechanics and not statistical mechanics to obtain the radiation resistance, we obtain

$$\beta_{\nu k,\mu}(t) = I_0\mathcal{F}_{\nu k}\delta_{\mu,0}e^{-i(\omega_\mu+\omega_{\rm d})t/2}\frac{\sin\left[(\omega_\mu-\omega_{\rm d})t/2\right]}{(\omega_\mu-\omega_{\rm d})/2}. \tag{S159}$$

Now, the energy $\mathcal{E}_{\rm F}$ radiated into the ferromagnet is

$$\begin{aligned}
\mathcal{E}_{\rm F} &= \sum_{k,\nu,\mu} \omega_\mu(k)|\beta_{\nu k,\mu}|^2, \\
&\to tL \int_0^\infty \frac{dk}{2\pi} \sum_{\nu=\pm1} \omega_0(k) \left|I_0 \mathcal{F}_{\nu k}\right|^2 \delta\left(\omega_0(k) - \omega_{\rm d}\right), \\
&= tL \sum_{\nu=\pm1} \frac{\omega_{\rm d}}{v_0\left(k_{\rm d}\right)} \left|I_0 \mathcal{F}_{\nu k_{\rm d}}\right|^2,
\end{aligned} \tag{S160}$$

where $t \to \infty$ is used in the second line, $k_{\rm d} \geq 0$ is the wavenumber of the on-resonant MSSW, i.e. $\omega_0(k_{\rm d}) = \omega_{\rm d}$, and $v_0(k) = d\omega_0(k)/dk$ is the MSSW group velocity. The radiation power per unit length is

$$\mathcal{P}_{\rm F}/L = \mathcal{E}_{\rm F}/tL = \sum_{\nu=\pm1} \frac{\omega_{\rm d}}{v_0\left(k_{\rm d}\right)} \left|I_0 \mathcal{F}_{\nu k_{\rm d}}\right|^2, \tag{S161}$$

and the radiation resistance per unit length is

$$\begin{aligned}
r_{\rm r} &= \frac{\mathcal{P}_{\rm F}/L}{I_{\rm RMS}^2} = \frac{\mathcal{P}_{\rm F}/L}{2I_0^2}, \\
&= \sum_{\nu=\pm1} \frac{\omega_{\rm d}}{2v_0\left(k_{\rm d}\right)} \left|\mathcal{F}_{\nu k_{\rm d}}\right|^2, \\
&= \frac{\mu_0 \omega_M}{4} \sum_{\nu=\pm1} \frac{\omega_{\rm d}/k_{\rm d}}{v_0\left(k_{\rm d}\right)} \left|\mathcal{M}_{\nu k_{\rm d}}\right|^2 \left|\left(\frac{\sin k_{\rm d} w/2}{k_{\rm d} w/2}\right)\right|^2 \equiv \sum_{\nu=\pm1} r_{\rm r}^{(\nu)},
\end{aligned} \tag{S162}$$

where we defined the radiation resistance per unit length for the MSSW traveling in the $\nu\hat{y}$ direction:

$$r_{\rm r}^{(\nu)} = \frac{\mu_0 \omega_M}{4} \frac{\omega_{\rm d}/k_{\rm d}}{v_0\left(k_{\rm d}\right)} \left|\mathcal{M}_{\nu k_{\rm d}}\right|^2 \left|\left(\frac{\sin k_{\rm d} w/2}{k_{\rm d} w/2}\right)\right|^2. \tag{S163}$$

Here, $I_{\rm RMS} = \sqrt{2} I_0$ is the root-mean-square current [note that we have $I = 2I_0 \cos(\omega_{\rm d} t)$]. The radiation resistance we obtained in (S163) is identical to that presented in (*27*) except that Eq. (S163) is for a specific geometry of the MSL. This confirms the validity of the Hamiltonian approach for the MSSW.

SIII Numerical evaluation of the Longitudinal relaxation rate induced by magnons

For the calculation of the magnon-induced longitudinal relaxation rates of the NV center, we employed the model provided in Ref. (*14*). The total Hamiltonian of our hybrid system is written as $\mathcal{H} = \mathcal{H}_{\text{NV}} + \mathcal{H}_{\text{m}} + \mathcal{H}_{\text{int}}$, where $\mathcal{H}_{\text{NV}}$ is the NV Hamiltonian, $\mathcal{H}_{\text{m}}$ is the magnon Hamiltonian, and $\mathcal{H}_{\text{int}}$ is the interaction Hamiltonian,

$$\mathcal{H}_{\text{NV}} = D_{\text{NV}} \left(\hat{n}_{\text{NV}} \cdot \mathbf{S}_{\text{NV}}\right)^2 + \gamma\mu_0 \mathbf{S}_{\text{NV}} \cdot \mathbf{H}_{\text{ext}}, \tag{S164}$$

$$\begin{aligned}\mathcal{H}_{\text{m}} = &-\mu_0 \int d\mathbf{r} \mathbf{H}_{\text{ext}} \cdot \mathbf{M}(\mathbf{r}) + \frac{\mu_0}{2} \int d\mathbf{r} \alpha_{\text{ex}}(\mathbf{r}) \nabla\mathbf{M} : \nabla\mathbf{M} \\ &+ \frac{\mu_0}{2} \int d\mathbf{r} d\mathbf{r}' (\nabla \cdot \mathbf{M}(\mathbf{r})) G\left(\mathbf{r} - \mathbf{r}'\right) \left(\nabla' \cdot \mathbf{M}\left(\mathbf{r}'\right)\right),\end{aligned} \tag{S165}$$

$$\mathcal{H}_{\text{int}} = \gamma\mu_0 \mathbf{S}_{\text{NV}} \cdot \nabla \int d\mathbf{r}' G\left(\mathbf{r} - \mathbf{r}'\right) \nabla' \cdot \mathbf{M}\left(\mathbf{r}'\right)\Big|_{\mathbf{r}=\mathbf{r}_0}. \tag{S166}$$

Here, $D_{\text{NV}} = 2\pi \times 2.87$ GHz is the zero-field splitting of the NV center, $\hat{n}_{\text{NV}}$ is the unit vector along the NV main symmetry axis, $\mathbf{S}_{\text{NV}}$ is the spin-1 operator of the NV center, $\gamma = 2\pi \times 2.8$ MHz/G, $\mathbf{H}_{\text{ext}}$ is the external magnetic field, $\mathbf{M}(\mathbf{r})$ is the magnetization with the constraint $|\mathbf{M}(\mathbf{r})| = M_{\text{s}}(\mathbf{r}) = M_{\text{s}}\mathcal{F}(\mathbf{r})$, M_{s} is the YIG saturation magnetization, $\mathcal{F}(\mathbf{r}) = 1$ (0) inside (outside) the ferromagnetic structure, $\alpha_{\text{ex}}(\mathbf{r}) = \alpha_{\text{ex}}\mathcal{F}(\mathbf{r})$, $\alpha_{\text{ex}} = \lambda_{\text{ex}}^2 = D_{\text{ex}}/\gamma\mu_0 M_{\text{s}}$ is the exchange-length squared, $D_{\text{ex}} = 5.4 \times 10^{-1}\ \gamma$ G μm^2 is the YIG exchange constant, the double-dot product is defined as $\nabla\mathbf{M} : \nabla\mathbf{M} = \partial_a M_b \partial^a M^b$ (summation over indices appearing twice are suppressed as usual), $\mathbf{r}_0$ is the NV position, and $G(\mathbf{r} - \mathbf{r}') = 1/4\pi|\mathbf{r} - \mathbf{r}'|$ is the Green's function for the (negative of the) Laplacian. We note that the first term in Eq. (S165) is the Zeeman energy, the second term is the exchange energy, and the third term is the magnetic dipole energy. Inclusion of both the second and the third term in Eq. (S165) results in the dipole-exchange magnons in ferromagnets.

After simplification using the ferromagnetic film geometry, the external magnetic field $\mathbf{H}_{\text{ext}} \propto \hat{z}$, NV main-symmetry axis $\hat{n}_{\text{NV}} = \hat{z}$, where $\hat{z}$ is the in-plane direction of the film, and restricting the

NV subspace into lower energy two states $\{|m_s = 0\rangle, |m_s = -1\rangle\}$, we obtain

$$\mathcal{H}_{\mathrm{NV}} = \frac{\omega_{\mathrm{NV}}}{2}\sigma_z \tag{S167}$$

$$\mathcal{H}_{\mathrm{m}} = \sum_{n=0}^{N}\int \frac{d\mathbf{k}}{(2\pi)^2}\omega_{\mathbf{k},n}\beta^{\dagger}_{\mathbf{k},n}\beta_{\mathbf{k},n}, \tag{S168}$$

$$\mathcal{H}_{\mathrm{int}} = \sum_{n=0}^{N}\int \frac{d\mathbf{k}}{(2\pi)^2}g_{\mathbf{k},n}\beta_{\mathbf{k},n}e^{i\mathbf{k}\cdot\boldsymbol{\rho}_0}\sigma_+ + \mathrm{H.c.}, \tag{S169}$$

where $\omega_{\mathrm{NV}} = D_{\mathrm{NV}} - \gamma\mu_0 H_{\mathrm{ext}}$ is the $|m_s = 0\rangle \leftrightarrow |m_s = -1\rangle$ NV transition frequency, $\sigma_z = |e\rangle\langle e| - |g\rangle\langle g|$, $\sigma_+ = |e\rangle\langle g|$, where $|g\rangle = |m_s = 0\rangle$ and $|e\rangle = |m_s = -1\rangle$, $\omega_{\mathbf{k},n}$ is the magnon frequency with wavevector $\mathbf{k} = k_y\hat{y} + k_z\hat{z}$ and thickness-directional mode label n, N is the highest thickness-mode label, $\beta^{\dagger}_{\mathbf{k},n}$ ($\beta_{\mathbf{k},n}$) is the magnon creation (annihilation) operator with label $(\mathbf{k}, n)$, and $g_{\mathbf{k},n}$ is the NV-magnon coupling constant.

Differently from Sec. SII, where we obtained the exact solution of the mode profile by dropping the exchange interaction, in this section we included the exchange interaction. Accordingly, the magnon dispersion relation and the mode profile are obtained by the numerical diagonalization of the matrix representation of the Hamiltonian expanded by a sufficient number of mode profile basis functions (labeled by n, with appropriate surface-spin pinning conditions). After the numerical diagonalization of the Hamiltonian, the thickness-mode label n labels the numerically obtained solution for the magnon modes, i.e. the exact solution for the mode profile is approximately expanded by the original set of basis functions that we started with. The thickness-directional mode label n in this section, therefore, corresponds to the exact mode label μ in Sec. SII when dropping the exchange interaction $D_{\mathrm{ex}} \to 0$ and considering the momentum sector $k_z = 0$.

The T_1 relaxation rate is evaluated by the numerical integration of (see Sec. SIII.4)

$$1/T_1 = \coth(\beta\omega_{\mathrm{NV}}/2)\sum_{n=0}^{N}\int \frac{d\mathbf{k}}{(2\pi)^2}|g_{\mathbf{k},n}|^2 2\pi\delta(\omega_{\mathbf{k},n} - \omega_{\mathrm{NV}}), \tag{S170}$$

where $\beta = k_{\mathrm{B}}T$ is the inverse temperature, k_{B} is the Boltzmann constant, T is temperature, and the delta function is replaced by a Lorentzian function with small linewidth for the numerical evaluation.

SIII.1 Calculation of the Magnon Hamiltonian

For the diagonalization of the magnon Hamiltonian Eq. (S165), we follow the approach provided in Ref. (*14*) except that now we have a film geometry. We apply the Holstein-Primakoff transformation:

$$M_{-}(\mathbf{r}) = \sqrt{2\gamma M_{\mathrm{s}}(\mathbf{r})}a(\mathbf{r})f\left(a^{*}(\mathbf{r})a(\mathbf{r})\right), \tag{S171}$$

$$M_{+}(\mathbf{r}) = \sqrt{2\gamma M_{\mathrm{s}}(\mathbf{r})}a^{*}(\mathbf{r})f\left(a^{*}(\mathbf{r})a(\mathbf{r})\right), \tag{S172}$$

$$M_{z}(\mathbf{r}) = M_{\mathrm{s}}(\mathbf{r}) - \gamma a^{*}(\mathbf{r})a(\mathbf{r}), \tag{S173}$$

where $M_{\pm}(\mathbf{r}) = M_x(\mathbf{r}) \pm iM_y(\mathbf{r})$, $M_z(\mathbf{r}) = \sqrt{[M_{\mathrm{s}}(\mathbf{r})]^2 - ([M_x(\mathbf{r})]^2 + [M_y(\mathbf{r})]^2)}$, and $f(x) = \sqrt{1-\gamma x/(2M_{\mathrm{s}}(\mathbf{r}))}$. Here, $a(\mathbf{r})$ and $a^{*}(\mathbf{r})$ are the complex canonical variables satisfying $\partial_t a(\mathbf{r}) = -i\delta\mathcal{H}/\delta a^{*}(\mathbf{r})$ and $\partial_t a^{*}(\mathbf{r}) = +i\delta\mathcal{H}/\delta a(\mathbf{r})$. We expand the complex canonical variables with the totally-free surface-spin condition following

$$a(\mathbf{r}) = \int \frac{d\mathbf{k}}{(2\pi)^2} e^{i\mathbf{k}\cdot\boldsymbol{\rho}} \sum_{n=0}^{N} f_n(x) a_{\mathbf{k},n}, \tag{S174}$$

$$a^{*}(\mathbf{r}) = \int \frac{d\mathbf{k}}{(2\pi)^2} e^{-i\mathbf{k}\cdot\boldsymbol{\rho}} \sum_{n=0}^{N} f_n(x) a^{*}_{\mathbf{k},n}, \tag{S175}$$

$$f_n(x) = \sqrt{\frac{2\mathcal{F}(x)}{(1+\delta_{n,0})\,d}}\cos\left(\kappa_n x\right), \tag{S176}$$

where $\kappa_n = n\pi/d,\ (n = 0, 1, 2, \cdots)$ and $\mathcal{F}(x) = \Theta(x)\Theta(d-x)$. The surface-spin pinning condition needs to be specified in this section, unlike Sec. SII, as we are working with nonzero exchange constant. However, it is suspected that the surface pinning condition does not matter for the case where the YIG thickness d is large, as we will include many basis functions for the diagonalization of the Hamiltonian and the final mode profile will be the linear combinations of all these modes at the end. After simplification and promoting the complex canonical variables to creation and annihilation operators, the magnon Hamiltonian Eq. (S165) becomes,

$$\mathcal{H}_{\mathrm{m}} = \frac{1}{2}\int \frac{d\mathbf{k}}{(2\pi)^2} \sum_{nn'} \left[a^{\dagger}_{\mathbf{k},n}, a_{-\mathbf{k},n}\right] \widehat{H}_{\mathbf{k},nn'} \begin{bmatrix} a_{\mathbf{k},n'} \\ a^{\dagger}_{-\mathbf{k},n'} \end{bmatrix}, \tag{S177}$$

where the commutation relation for these operators is $[a_{\mathbf{k},n}, a^\dagger_{\mathbf{k}',n'}] = (2\pi)^2\delta(\mathbf{k}-\mathbf{k}')\delta_{n,n'}$, and the Hamiltonian matrix is given by

$$\begin{aligned}\hat{H}_{\mathbf{k},nn'} := &\quad (\omega_H I_{2\times2}\delta_{nn'}) + \left(D_{\rm ex}K_n^2 I_{2\times2}\delta_{nn'}\right) \\ &+\frac{\omega_M}{2}\left(\begin{bmatrix}1 & 1\\ 1 & 1\end{bmatrix}\delta_{nn'} - \begin{bmatrix}1-\sin^2\phi_{\mathbf{k}} & 1+\sin^2\phi_{\mathbf{k}}\\ 1+\sin^2\phi_{\mathbf{k}} & 1-\sin^2\phi_{\mathbf{k}}\end{bmatrix}P_{nn'}(k) - \begin{bmatrix}0 & -4\\ 4 & 0\end{bmatrix}\sin\phi_{\mathbf{k}}Q_{nn'}(k)\right), \\ = &\quad \left(\Omega_{k,n}I_{2\times2} + \frac{\omega_M}{2}\begin{bmatrix}1 & 1\\ 1 & 1\end{bmatrix}\right)\delta_{nn'} \\ &-\frac{\omega_M}{2}\left(\begin{bmatrix}1-\sin^2\phi_{\mathbf{k}} & 1+\sin^2\phi_{\mathbf{k}}\\ 1+\sin^2\phi_{\mathbf{k}} & 1-\sin^2\phi_{\mathbf{k}}\end{bmatrix}P_{nn'}(k) + \begin{bmatrix}0 & -4\\ 4 & 0\end{bmatrix}\sin\phi_{\mathbf{k}}Q_{nn'}(k)\right), \end{aligned} \tag{S178}$$

where $I_{2\times2}$ is the 2×2 identity matrix, $\cos\phi_{\mathbf{k}} = \frac{\mathbf{k}\cdot\mathbf{H}_{\rm ext}}{|\mathbf{k}||\mathbf{H}_{\rm ext}|}$, and

$$\Omega_{k,n} = \omega_H + D_{\rm ex}K_n^2, \tag{S179}$$

$$K_n^2 = k^2 + \kappa_n^2. \tag{S180}$$

with $k = |\mathbf{k}|$. The functions $P_{nn'}(k)$ and $Q_{nn'}(k)$ are symmetric and anti-symmetric matrix with respect to their indices:

$$\begin{aligned}&P_{nn'}(k) = P_{n'n}(k) \\ &= \frac{k^2}{K_n^2}\delta_{nn'} - \frac{1}{\left[(1+\delta_{n,0})(1+\delta_{n',0})\right]^{1/2}}\frac{k^4}{K_n^2K_{n'}^2}F_n(k)\delta_{par(n),par(n')}\end{aligned} \tag{S181}$$

$$\begin{aligned}&Q_{nn'}(k) = -Q_{n'n}(k) \\ &= \frac{k^2}{K_{n'}^2}\left(\frac{\kappa_{n'}^2}{\kappa_{n'}^2-\kappa_n^2}\cdot\frac{2}{kd} - \frac{k^2}{2K_n^2}F_n(k)\right)\frac{1}{\left[(1+\delta_{n,0})(1+\delta_{n',0})\right]^{1/2}}1_{par(n)\neq par(n)},\end{aligned} \tag{S182}$$

where $F_n(k) = 2\frac{1-(-1)^n e^{-kd}}{kd}$, and $1_{par(n)\neq par(n')}$ is one when the parity of n is not equal to that of n' and zero otherwise. In the first lines of Eq. (S178), the first, second, and third terms correspond to the first, second, and third terms in Eq. (S165), respectively.

The above expression Eq. (S178) is almost identical to the one presented in the Ref. (*30*), though there is a minor sign mismatch associated with the different definitions of the sign of the two-dimensional Fourier transform and the coordinate system. We also note that while there may be some prefactor differences in $Q_{nn'}(k)$ with respect to Ref. (*30*), we verified that the above expression is correct and in fact is the same as in Refs. (*31, 32*) from the same authors. We also numerically

verified that the above expression is accurate by checking the limit $D_{\rm ex} \to 0$ and considering the momentum sector with $k_z = 0$. For this limit, the numerical evaluation approximately results in the same mode profile and the dispersion relation provided in Sec. SII, although we need to include many basis functions for the numerical evaluation so that we can sufficiently expand, for example, the exponential mode function $\xi^{(\mu=0)}_{\nu k}(x) \propto e^{\nu k x}$ [in Eq. (S79)] with the basis set of functions Eq. (S176). Additionally, we numerically verified that the same observation of recovering the mode profile and the dispersion relation provided in Sec. SII applies when we expand the mode functions with the totally-pinned surface-spin condition (*30*).

SIII.1.1 alternative case with the external field not parallel to the film surface

When the field is applied not parallel to the film and there is normal component of the external field, we will follow the exact procedure presented in Ref. (*30*). We firstly need to find the equilibrium magnetization orientation, along which we will define the $\hat{z}$ direction. This is obtained by solving

$$\mathbf{H}_0 = \mathbf{H}_{\rm ext} - \hat{n} M_s \cos\theta, \tag{S183}$$

where the second term is the demagnetization field produced by the static magnetization, $\hat{n}$ is the normal direction of the film, and θ is the angle between $\hat{n}$ and the internal field direction $\mathbf{H}_0$. Eq. (S183) can be transformed into two conditions $H_0 \cos\theta = H_{\rm ext}\cos\theta_{\rm ext} - M_s \cos\theta$ and $M_s \sin 2\theta = 2H_{\rm ext}\sin(\theta - \theta_{\rm ext})$ as presented in Ref. (*30*), where $\theta_{\rm ext}$ is the angle between the external field $\mathbf{H}_{\rm ext}$ and the normal direction $\hat{n}$. By defining $\hat{z} = \mathbf{H}_0/|\mathbf{H}_0|$ and $\omega_H = \gamma\mu_0 H_0$ (not $\omega_H = \gamma\mu_0 H_{\rm ext}$), the corresponding magnon Hamiltonian matrix reads

$$\begin{aligned}
&\hat{H}_{\mathbf{k},nn'} \\
&:= \Omega_{k,n}\delta_{nn'} I_{2\times 2} + \frac{\omega_M \sin^2\theta}{2}\begin{bmatrix} 1 & 1 \\ 1 & 1 \end{bmatrix}\delta_{nn'} \\
&+ \frac{\omega_M}{2}\begin{bmatrix} (A+E)P_{nn'}(k) - iBQ_{nn'}(k) & (A-2iC-E)P_{nn'}(k) - (iB+2D)Q_{nn'}(k) \\ (A+2iC-E)P_{nn'}(k) - (iB-2D)Q_{nn'}(k) & (A+E)P_{nn'}(k) - iBQ_{nn'}(k) \end{bmatrix},
\end{aligned} \tag{S184}$$

where $\phi_{\mathbf{k}}$ is the angle between $\mathbf{k}$ and $\mathbf{H}_0^{\parallel} = \mathbf{H}_0 - \hat{n}(\hat{n}\cdot\mathbf{H}_0)$, i.e. the internal field projected onto the film plane. Here, $A, B, C, D,$ and E are geometric factors that depend on the direction of the

equilibrium magnetization and the spin wave propagation:

$$A = \cos^2\phi_{\mathbf{k}} - \sin^2\theta\left(1+\cos^2\phi_{\mathbf{k}}\right) \quad \text{(S185)}$$

$$B = 2\sin 2\theta\cos\phi_{\mathbf{k}} \quad \text{(S186)}$$

$$C = -\cos\theta\cos\phi_{\mathbf{k}}\sin\phi_{\mathbf{k}} \quad \text{(S187)}$$

$$D = -2\sin\theta\sin\phi_{\mathbf{k}} \quad \text{(S188)}$$

$$E = \sin^2\phi_{\mathbf{k}}. \quad \text{(S189)}$$

We notice that Eq. (S178) corresponds to the $\theta = \pi/2$ case of Eq. (S184).

SIII.2 Obtaining magnon spectrum by paraunitary diagonalization

To obtain the magnon-mode frequencies and mode profiles, we need to diagonalize the magnon Hamiltonian Eq. (S177) with the paraunitary matrix approach (*33*). This is done by firstly writing Eq. (S177) into the form

$$\mathcal{H}_{\rm m} = \frac{1}{2}\int \frac{d\mathbf{k}}{(2\pi)^2}\left[\boldsymbol{\alpha}^{\dagger}_{\mathbf{k}}, \boldsymbol{\alpha}_{-\mathbf{k}}\right]\mathbf{H}_{\mathbf{k}}\begin{bmatrix}\boldsymbol{\alpha}_{\mathbf{k}}\\ \boldsymbol{\alpha}^{\dagger}_{-\mathbf{k}}\end{bmatrix}, \quad \text{(S190)}$$

where $\boldsymbol{\alpha}^{\dagger}_{\mathbf{k}} = [a^{\dagger}_{\mathbf{k},0}, a^{\dagger}_{\mathbf{k},1}, \cdots, a^{\dagger}_{\mathbf{k},N}]$ and $\boldsymbol{\alpha}_{\mathbf{k}} = [a_{\mathbf{k},0}, a_{\mathbf{k},1}, \cdots, a_{\mathbf{k},N}]$ are the spin-wave creation and annihilation operators, respectively, for the lowest $N+1$ modes. No confusion is expected for the column or row vectors as conventionally written in Ref. (*33*). The $2(N+1)\times 2(N+1)$ Hermitian matrix $\mathbf{H}_{\mathbf{k}}$ can be decomposed into four blocks of $(N+1)\times(N+1)$ square matrices (*33*),

$$\mathbf{H}_{\mathbf{k}} = \begin{bmatrix}\mathbf{A}_{\mathbf{k}} & \mathbf{B}_{\mathbf{k}}\\ \mathbf{B}^{*}_{-\mathbf{k}} & \mathbf{A}^{*}_{-\mathbf{k}}\end{bmatrix}, \quad \text{(S191)}$$

where the asterisk of matrices indicates the complex conjugation of the elements, i.e., $[\mathbf{D}^*]_{ij} = ([\mathbf{D}]_{ij})^*$ for a matrix $\mathbf{D}$. This should not be confused with the dagger symbol indicating the conjugate transpose $[\mathbf{D}^{\dagger}]_{ij} = ([\mathbf{D}]_{ji})^*$. The matrix $\mathbf{A}_{\mathbf{k}}$ is a Hermitian matrix which is sometimes called the normal parts (particle-hole channel), while the matrix $\mathbf{B}_{\mathbf{k}}$ is a matrix satisfying $\mathbf{B}^{T}_{-\mathbf{k}} = \mathbf{B}_{\mathbf{k}}$ which is sometimes is called the anomalous parts (particle-particle channel) (*34*). For magnons in magnetic

films, comparing Eqs. (S191) and (S178), we obtain

$$[\mathbf{A_k}]_{nn'} = \left(\Omega_{k,n} + \frac{\omega_M}{2}\right)\delta_{nn'} - \frac{\omega_M}{2}\left[(1-\sin^2\phi_{\mathbf{k}})P_{nn'}(k)\right], \tag{S192}$$

$$[\mathbf{B_k}]_{nn'} = \frac{\omega_M}{2}\delta_{nn'} - \frac{\omega_M}{2}\left[(1+\sin^2\phi_{\mathbf{k}})P_{nn'}(k) - 4\sin\phi_{\mathbf{k}}Q_{nn'}(k)\right]. \tag{S193}$$

We introduce a paraunitary matrix $\mathbf{T_k}$, which is a $2(N+1)$ by $2(N+1)$ matrix satisfying

$$\mathbf{T}_\mathbf{k}^\dagger \boldsymbol{\sigma}_z \mathbf{T_k} = \mathbf{T_k}\boldsymbol{\sigma}_z\mathbf{T}_\mathbf{k}^\dagger = \boldsymbol{\sigma}_z, \tag{S194}$$

where $\boldsymbol{\sigma}_z = \mathrm{diag}[+1,+1,\cdots,+1,-1,-1,\cdots,-1]$ is $+1$ for the first $(N+1)$ diagonal components and -1 for the remaining $(N+1)$ diagonal components. With this paraunitary matrix $\mathbf{T_k}$, we hope to diagonalize the $2(N+1)$ by $2(N+1)$ matrix $\mathbf{H_k}$, that is,

$$\mathbf{T}_\mathbf{k}^\dagger\mathbf{H_k}\mathbf{T_k} = \begin{bmatrix} \mathbf{E_k} & \mathbf{0} \\ \mathbf{0} & \mathbf{E_{-k}} \end{bmatrix} = \boldsymbol{\mathcal{E}}_{\mathrm{k}}, \tag{S195}$$

where $\mathbf{E_k} = \mathrm{diag}[\omega_{\mathbf{k},0},\omega_{\mathbf{k},1},\cdots,\omega_{\mathbf{k},N}]$ is the $(N+1)$ by $(N+1)$ diagonal matrix with real and positive entries, which gives the frequencies $\omega_{\mathbf{k},n}$ of the spin-wave eigenmodes with in-plane wave vector $\mathbf{k}$ and thickness-mode label n.

The paraunitary matrix can be found through a method based on the Cholesky decomposition (*33*). Firstly, we decompose $\mathbf{H_k}$ into a product of an upper trianglar matrix and its Hermitian conjugate through the Cholesky decomposition:

$$\mathbf{H_k} = \mathbf{K}_\mathbf{k}^\dagger\mathbf{K_k}. \tag{S196}$$

Next, we define a new Hermitian matrix $\mathbf{W_k} = \mathbf{K_k}\boldsymbol{\sigma}_z\mathbf{K}_\mathbf{k}^\dagger$ and diagonalize it with a unitary matrix $\mathbf{U_k}$. Then we get

$$\mathbf{U}_\mathbf{k}^\dagger\mathbf{W_k}\mathbf{U_k} = \begin{bmatrix} \mathbf{E_k} & \mathbf{0} \\ \mathbf{0} & -\mathbf{E_{-k}} \end{bmatrix}. \tag{S197}$$

Lastly, we define the following matrix $\mathbf{T_k}$, which turns out to be a paraunitary matrix.

$$\mathbf{T_k} = \mathbf{K}_\mathbf{k}^{-1}\mathbf{U_k}\begin{bmatrix} \mathbf{E}_\mathbf{k}^{1/2} & \mathbf{0} \\ \mathbf{0} & -\mathbf{E}_\mathbf{-k}^{1/2} \end{bmatrix}. \tag{S198}$$

We define creation/annihilation operators $\boldsymbol{\beta}_{\mathbf{k}}^\dagger$ and $\boldsymbol{\beta}_{\mathbf{k}}$ via

$$\begin{bmatrix} \boldsymbol{\alpha}_{\mathbf{k}} \\ \boldsymbol{\alpha}_{-\mathbf{k}}^\dagger \end{bmatrix} = \mathbf{T}_{\mathbf{k}} \begin{bmatrix} \boldsymbol{\beta}_{\mathbf{k}} \\ \boldsymbol{\beta}_{-\mathbf{k}}^\dagger \end{bmatrix}. \tag{S199}$$

The paraunitarity of the matrix $\mathbf{T}_{\mathbf{k}}$ ensures that the operators $\beta_{\mathbf{k},n}^\dagger$'s and $\beta_{\mathbf{k},n}$'s have correct commutation relations for creation/annihilation operators $[\beta_{\mathbf{k},n}, \beta_{\mathbf{k}',n'}^\dagger] = (2\pi)^2\delta(\mathbf{k}-\mathbf{k}')\delta_{n,n'}$. Then we get the diagonalized Hamiltonian

$$\mathcal{H}_{\mathrm{m}} = \sum_{\mathbf{k},n} \omega_{\mathbf{k},n}\beta_{\mathbf{k},n}^\dagger\beta_{\mathbf{k},n}, \tag{S200}$$

which successfully diagonalizes the magnon Hamiltonian with correct Bogoliubov transformation coefficients. We note that we can write the paraunitary matrix $\mathbf{T}_{\mathbf{k}}$ in terms of four $(N+1)$ by $(N+1)$ matrices:

$$\mathbf{T}_{\mathbf{k}} = \begin{bmatrix} \mathbf{T}_{\mathbf{k}}^{pp} & \mathbf{T}_{\mathbf{k}}^{pn} \\ \mathbf{T}_{\mathbf{k}}^{np} & \mathbf{T}_{\mathbf{k}}^{nn} \end{bmatrix}. \tag{S201}$$

We notice that there is a symmetry of the Hamiltonian matrix $\mathbf{H}_{-\mathbf{k}} = \boldsymbol{\sigma}_x\mathbf{H}_{\mathbf{k}}^*\boldsymbol{\sigma}_x$ (*34*), where $\boldsymbol{\sigma}_x$ is the $2(N+1)\times 2(N+1)$ matrix analogous to $\boldsymbol{\sigma}_z$ defined by

$$\boldsymbol{\sigma}_x = \begin{bmatrix} \mathbf{O} & \mathbf{I} \\ \mathbf{I} & \mathbf{O} \end{bmatrix}, \tag{S202}$$

where $\mathbf{I}$ and $\mathbf{O}$ are $(N+1)\times(N+1)$ identity and zero matrices, respectively. This allows us to choose $\mathbf{T}_{\mathbf{k}}$ to be written in terms of $\mathbf{T}_{-\mathbf{k}}$:

$$\mathbf{T}_{-\mathbf{k}} = \boldsymbol{\sigma}_x\mathbf{T}_{\mathbf{k}}^*\boldsymbol{\sigma}_x = \begin{bmatrix} (\mathbf{T}_{\mathbf{k}}^{nn})^* & (\mathbf{T}_{\mathbf{k}}^{np})^* \\ (\mathbf{T}_{\mathbf{k}}^{pn})^* & (\mathbf{T}_{\mathbf{k}}^{pp})^* \end{bmatrix}. \tag{S203}$$

This means that we only need to perform paraunitary diagonalization in the half of the entire two-dimensional space of $\mathbf{k}$ when performing the numerical computation. We also note that, as compared to the MSSW formulation provided in Sec. SII, the paraunitary matrices used in this section and the mode functions shown in Eqs. (S71) and (S72) are related by

$$\xi_{\mathbf{k},n}(x) = [\mathbf{f}(x)\cdot\mathbf{T}_{\mathbf{k}}^{pp}]_n = \sum_{m=0}^{N} f_m(x)[\mathbf{T}_{\mathbf{k}}^{pp}]_{mn}, \tag{S204}$$

$$\eta_{\mathbf{k},n}(x) = [\mathbf{f}(x)\cdot\mathbf{T}_{\mathbf{k}}^{np}]_n = \sum_{m=0}^{N} f_m(x)[\mathbf{T}_{\mathbf{k}}^{np}]_{mn}, \tag{S205}$$

where $\mathbf{f}(x) = [f_0(x), f_1(x), \cdots, f_N(x)]$ is the list of basis functions defined in Eq. (S176). This can be verified by setting the exchange stiffness parameter to zero $D_{\mathrm{ex}} = 0$ and check that one of the pairs of magnon mode functions defined by Eqs. (S204) and (S205) [for example, a pair of mode functions $(\xi_{\mathbf{k},n}(x), \eta_{\mathbf{k},n}(x))$ labeled by $n = n_{\mathrm{MSSW}}$] automatically become the functions defined in Eqs. (S79) and (S80) for the surface-localized mode labeled by $\mu = 0$. However, modes with $\mu \neq 0$ are energy degenerate and therefore we do not expect to reproduce the functions defined in Eqs. (S79) and (S79) from Eqs. (S204) and (S205).

SIII.2.1 Proof of the lower boundedness of the magnon energy by ω_H

Here we show that the magnon energy for the Hamiltonian matrix given by Eq. (S178) is lower bounded by ω_H. To this end, we write the three matrices in the first line of Eq. (S178) in the form of Eq. (S190), i.e.

$$\mathbf{H}_{\mathbf{k}} = \mathbf{H}_{\mathbf{k}}^{\mathrm{Z}} + \mathbf{H}_{\mathbf{k}}^{\mathrm{ex}} + \mathbf{H}_{\mathbf{k}}^{\mathrm{dip}}, \tag{S206}$$

where $\mathbf{H}_{\mathbf{k}}^{\mathrm{Z}}$, $\mathbf{H}_{\mathbf{k}}^{\mathrm{ex}}$, and $\mathbf{H}_{\mathbf{k}}^{\mathrm{dip}}$ are the Zeeman, exchange, and dipole interaction Hamiltonian matrices, respectively. As the three matrices on the right-hand side of Eq. (S206) are all positive-semidefinite matrices, it is tempting to argue that the magnon energy is lower bounded by ω_H because of

$$(\text{minimum eigenvalue of } \mathbf{H}_{\mathbf{k}}) \geq \left(\text{minimum eigenvalue of } \mathbf{H}_{\mathbf{k}}^{\mathrm{Z}}\right) = \omega_H. \tag{S207}$$

However, as we are dealing with a bosonic Hamiltonian matrix, this argument is incorrect because the magnon energies are *not* the eigenvalues of $\mathbf{H}_{\mathbf{k}}$ unlike the fermionic Hamiltonian case. Instead, the magnon energies are the eigenvalues of the generalized eigenvalue problem (*33*)

$$\mathbf{H}_{\mathbf{k}}\vec{w} = \lambda \boldsymbol{\sigma}_z \vec{w}, \tag{S208}$$

where λ is the generalized eigenvalue and $\vec{w}$ is the generalized eigenvector. It is obtained from the classical Hamiltonian equations of motion by finding the solution taking the form of $(\boldsymbol{\alpha}_{\mathbf{k}}(t), \boldsymbol{\alpha}^*_{-\mathbf{k}}(t)) = \vec{w}^T e^{-i\lambda t}$. It is notable that the generalized eigenvalue λ assumes both positive and negative values even though $\mathbf{H}_{\mathbf{k}}$ is positive semidefinite. This is not surprising because the Hamiltonian equations of motion give both positive-frequency and negative-frequency solutions. For example, even when we drop

$\mathbf{H}^{\rm ex}_{\mathbf{k}}$ and $\mathbf{H}^{\rm dip}_{\mathbf{k}}$, we have solutions for Eq. (S208) such as

$$\vec{w}^T = (a_{\mathbf{k},0}, a_{\mathbf{k},1}, \cdots, a_{\mathbf{k},N}; a^*_{-\mathbf{k},0}, a^*_{-\mathbf{k},1}, \cdots, a^*_{-\mathbf{k},N}) = (1, 0, \cdots, 0; 0, 0, \cdots, 0), \text{ with } \lambda = \omega_H, \tag{S209}$$

$$\vec{w}^T = (a_{\mathbf{k},0}, a_{\mathbf{k},1}, \cdots, a_{\mathbf{k},N}; a^*_{-\mathbf{k},0}, a^*_{-\mathbf{k},1}, \cdots, a^*_{-\mathbf{k},N}) = (0, 0, \cdots, 0; 1, 0, \cdots, 0), \text{ with } \lambda = -\omega_H, \tag{S210}$$

where the semicolon is used only to separate the first $(N+1)$ elements from the remaining $(N+1)$ ones. Therefore, we would like to show that $|\lambda|$ is lower bounded by ω_H. To make $\mathbf{H}_{\mathbf{k}}$ positive definite (i.e. no zero eigenvalues), we consider the case with $\omega_H > 0$ in the following paragraph.

To show that the absolute value of the generalized eigenvalue λ in Eq. (S208) is lower bounded by ω_H, we first transform Eq. (S208) into a standard eigenvalue problem of a Hermitian matrix $\sqrt{\mathbf{H}_{\mathbf{k}}}\boldsymbol{\sigma}_z\sqrt{\mathbf{H}_{\mathbf{k}}}$, i.e.

$$\sqrt{\mathbf{H}_{\mathbf{k}}}\boldsymbol{\sigma}_z\sqrt{\mathbf{H}_{\mathbf{k}}}\vec{u} = \lambda\vec{u}, \tag{S211}$$

where $\vec{u} = \sqrt{\mathbf{H}_{\mathbf{k}}}\vec{w}$. Here, the square root of the matrix $\mathbf{H}_{\mathbf{k}}$ is well-defined as it is a positive-semidefinite Hermitian matrix. More concretely, as it can be diagonalized using a diagonal matrix with non-negative entries and a unitary matrix, it is known that the square root of the matrix can be defined by taking the square root of the entries of the diagonal matrix and then sandwiching it by the unitary matrix and its adjoint. Therefore, we have shown the magnon energies are the eigenvalues of $\sqrt{\mathbf{H}_{\mathbf{k}}}\boldsymbol{\sigma}_z\sqrt{\mathbf{H}_{\mathbf{k}}}$, instead of the eigenvalues of $\mathbf{H}_{\mathbf{k}}$. Denoting the singular values of a matrix $\mathbf{D}$ as $\sigma_i(\mathbf{D})$, we obtain

$$(\text{minimum magnon energy}) = \min_i \sigma_i\left(\sqrt{\mathbf{H}_{\mathbf{k}}}\boldsymbol{\sigma}_z\sqrt{\mathbf{H}_{\mathbf{k}}}\right). \tag{S212}$$

As a reminder, the singular values of a matrix $\mathbf{D}$ are the square roots of the eigenvalues of the matrix $\mathbf{D}^\dagger\mathbf{D}$. When $\mathbf{D}$ is a positive-definite Hermitian matrix, singular values are eigenvalues. Next, we show that the singular values of $\sqrt{\mathbf{H}_{\mathbf{k}}}\boldsymbol{\sigma}_z\sqrt{\mathbf{H}_{\mathbf{k}}}$ are lower bounded by ω_H. To show it, we transform

$$\begin{aligned}\min_i \sigma_i\left(\sqrt{\mathbf{H}_{\mathbf{k}}}\boldsymbol{\sigma}_z\sqrt{\mathbf{H}_{\mathbf{k}}}\right) &= 1\Big/\max_i \sigma_i\left(\left(\sqrt{\mathbf{H}_{\mathbf{k}}}\boldsymbol{\sigma}_z\sqrt{\mathbf{H}_{\mathbf{k}}}\right)^{-1}\right), \\ &= 1\Big/\max_i \sigma_i\left(\sqrt{\mathbf{H}^{-1}_{\mathbf{k}}}\boldsymbol{\sigma}_z\sqrt{\mathbf{H}^{-1}_{\mathbf{k}}}\right) = 1\Big/\left\|\sqrt{\mathbf{H}^{-1}_{\mathbf{k}}}\boldsymbol{\sigma}_z\sqrt{\mathbf{H}^{-1}_{\mathbf{k}}}\right\|,\end{aligned} \tag{S213}$$

where we used $\boldsymbol{\sigma}_z^{-1} = \boldsymbol{\sigma}_z$ in the second line and $\max_i \sigma(\mathbf{D}) = \|\mathbf{D}\|$ in the last equality with the spectral norm $\|\cdots\|$. As a reminder, the spectral norm is the matrix norm induced by the vector p-norm with $p = 2$. More specifically, the spectral norm of a matrix $\mathbf{D}$ is defined as $\|\mathbf{D}\|\equiv\|\mathbf{D}\|_{p=2}$ for $\|\mathbf{D}\|_p\equiv \sup_{x\neq 0} \frac{\|\mathbf{D}x\|_p}{\|x\|_p}$, where $\|x\|_p = (\sum_i |x_i|^p)^{1/p}$ is the vector p-norm (l^p-norm) and $\|\cdots\|_p$ is the matrix norm induced by the l^p-norm. Using the sub-multiplicativity ($\|\mathbf{AB}\|\leq\|\mathbf{A}\|\|\mathbf{B}\|$ for two matrices $\mathbf{A}$ and $\mathbf{B}$) of the matrix norm, the denominator of the last expression of Eq. (S213) can be evaluated as

$$\left\|\sqrt{\mathbf{H}_{\mathbf{k}}^{-1}}\boldsymbol{\sigma}_z\sqrt{\mathbf{H}_{\mathbf{k}}^{-1}}\right\| \leq \left\|\sqrt{\mathbf{H}_{\mathbf{k}}^{-1}}\right\| \|\boldsymbol{\sigma}_z\| \left\|\sqrt{\mathbf{H}_{\mathbf{k}}^{-1}}\right\| = \|\mathbf{H}_{\mathbf{k}}^{-1}\|, \tag{S214}$$

where we used $\|\boldsymbol{\sigma}_z\| = 1$ and $\|\sqrt{\mathbf{H}_{\mathbf{k}}^{-1}}\|^2 = \|\mathbf{H}_{\mathbf{k}}^{-1}\|$. Note that the sub-multiplicativity can be shown by firstly obtaining the relation $\|\mathbf{AB}x\| \leq \|\mathbf{A}\|\|\mathbf{B}x\| \leq \|\mathbf{A}\|\|\mathbf{B}\|\|x\|$ from the definition of the matrix norm for a vector x. Then we obtain the desired sub-multiplicativity relation by dividing both-hand sides of this inequality with $\|x\| \neq 0$ and taking a supremum. On the righ-hand side of Eq. (S214), as the eigenvalues of $\mathbf{H}_{\mathbf{k}}$ is lower bounded by ω_H [see Eq.(S207)], the eigenvalues of $\mathbf{H}_{\mathbf{k}}^{-1}$ is upper bounded by $1/\omega_H$, i.e.

$$\|\mathbf{H}_{\mathbf{k}}^{-1}\| \leq 1/\omega_H. \tag{S215}$$

Combining Eqs. (S213-(S215), we obtain

$$\min_i \sigma_i\left(\sqrt{\mathbf{H}_{\mathbf{k}}}\boldsymbol{\sigma}_z\sqrt{\mathbf{H}_{\mathbf{k}}}\right) \geq \omega_H. \tag{S216}$$

Finally, using Eqs. (S212) and (S216), we obtain

$$(\text{minimum magnon energy}) \geq \omega_H. \tag{S217}$$

SIII.3 Obtaining NV-magnon coupling using paraunitary matrix

The NV-magnon coupling $g_{\mathbf{k},n}$ can be obtained by computing Eq. (S166) by firstly evaluating the following magnon-induced magnetic field:

$$\mathbf{h}(\mathbf{r})=\int\frac{d\mathbf{k}}{(2\pi)^2}e^{i\mathbf{k}\cdot\boldsymbol{\rho}}\mathbf{h}_{\mathbf{k}}(x) = \nabla\int d\mathbf{r}'G\left(\mathbf{r}-\mathbf{r}'\right)\nabla'\cdot\mathbf{M}\left(\mathbf{r}'\right), \tag{S218}$$

$$= \int d\mathbf{r}'\hat{D}\left(\mathbf{r}-\mathbf{r}'\right)\cdot\mathbf{M}\left(\mathbf{r}'\right),$$

$$= \int\frac{d\mathbf{k}}{(2\pi)^2}e^{i\mathbf{k}\cdot\boldsymbol{\rho}}\int dx'\hat{D}_{\mathbf{k}}\left(x-x'\right)\cdot\mathbf{M}_{\mathbf{k}}\left(x'\right), \tag{S219}$$

where we defined

$$\hat{D}\left(\mathbf{r}-\mathbf{r}'\right)=\int\frac{d\mathbf{k}}{(2\pi)^2}e^{i\mathbf{k}\cdot(\boldsymbol{\rho}-\boldsymbol{\rho}')}D_{\mathbf{k}}(x-x')=-(\nabla\otimes\nabla')G\left(\mathbf{r}-\mathbf{r}'\right), \tag{S220}$$

$$\mathbf{M}(\mathbf{r})=\int\frac{d\mathbf{k}}{(2\pi)^2}e^{i\mathbf{k}\cdot\boldsymbol{\rho}}\mathbf{M}_{\mathbf{k}}\left(x\right), \tag{S221}$$

and $\hat{D}_{\mathbf{k}}(x-x')$ is provided in Eq. (S65). Comparing Eqs. (S171)-(S175) and (S221), we can write $\mathbf{M}_{\mathbf{k}}\left(x\right)$ in terms of the creation/annihilation operators. Up to linear order in the creation/annihilation operators, we obtain

$$\mathbf{M}_{\mathbf{k}}\approx\sqrt{2\gamma M_{\mathrm{s}}(x)}\frac{\hat{e}_{+}a_{\mathbf{k}}(x)+\hat{e}_{-}a^{*}_{\mathbf{k}}(x)}{2}+(2\pi)^2\delta(\mathbf{k})M_{\mathrm{s}}(x)\hat{z}, \tag{S222}$$

where we defined non-normalized circular polarization vectors:

$$\hat{e}_{\pm}=\hat{x}\pm i\hat{y}. \tag{S223}$$

The magnon-induced magnetic field in Fourier space becomes

$$\mathbf{h}_{\mathbf{k}}(x) = \int dx'\hat{D}_{\mathbf{k}}(x-x')\cdot\mathbf{M}_{\mathbf{k}}(x'),$$

$$= \sqrt{2\gamma M_{\mathrm{s}}/d}\left(\hat{\sigma}^{+}_{-\mathbf{k}}\otimes\hat{\sigma}^{+}_{-\mathbf{k}}\right)\bar{\mathbf{f}}_{k}(x)\cdot\frac{\hat{e}_{+}\boldsymbol{\alpha}_{\mathbf{k}}+\hat{e}_{-}\boldsymbol{\alpha}^{\dagger}_{-\mathbf{k}}}{2},$$

$$= \sqrt{2\gamma M_{\mathrm{s}}/d}\,\hat{\sigma}^{+}_{-\mathbf{k}}\mathbf{w}_{\mathbf{k}}\begin{bmatrix}\boldsymbol{\alpha}_{\mathbf{k}}\\ \boldsymbol{\alpha}^{\dagger}_{-\mathbf{k}}\end{bmatrix}, \tag{S224}$$

where we defined (for $x > d$)

$$\bar{\mathbf{f}}_k(x) = [\bar{f}_{k,0}(x), \bar{f}_{k,1}(x), \cdots, \bar{f}_{k,N}(x)], \tag{S225}$$

$$\bar{f}_{k,n}(x) = \int_0^d dx' \frac{k e^{-k(x-x')}\sqrt{d} f_n(x')}{2} = \frac{(-1)^n}{\sqrt{2(1+\delta_{n,0})}} \frac{k^2}{K_n^2} e^{-k(x-d)} \left(1 - (-1)^n e^{-kd}\right), \tag{S226}$$

$$\mathbf{w}_{\mathbf{k}} \equiv (1/2)\left[(1+\sin\phi_{\mathbf{k}})\,\bar{\mathbf{f}}_k(x) \quad (1-\sin\phi_{\mathbf{k}})\,\bar{\mathbf{f}}_k(x)\right], \tag{S227}$$

where $\phi_{\mathbf{k}}$ is the angle between $\mathbf{k}$ and $\hat{z}$ such that $\hat{k} = \sin\phi_{\mathbf{k}}\hat{y} + \cos\phi_{\mathbf{k}}\hat{z}$. In terms of the correct magnon basis $\boldsymbol{\beta}_{\mathbf{k}}$, we can write

$$\begin{aligned}
\mathbf{h}_{\mathbf{k}}(x) &= \sqrt{2\gamma M_{\mathrm{s}}/d}\,\hat{\sigma}^+_{-\mathbf{k}}\mathbf{w}_{\mathbf{k}}\mathbf{T}_{\mathbf{k}}\begin{bmatrix}\boldsymbol{\beta}_{\mathbf{k}}\\ \boldsymbol{\beta}^\dagger_{-\mathbf{k}}\end{bmatrix}, \\
&= \sqrt{2\gamma M_{\mathrm{s}}/d}\,\hat{\sigma}^+_{-\mathbf{k}}\mathbf{w}_{\mathbf{k}}\begin{bmatrix}\mathbf{T}^{pp}_{\mathbf{k}} & \mathbf{T}^{pn}_{\mathbf{k}}\\ \mathbf{T}^{np}_{\mathbf{k}} & \mathbf{T}^{nn}_{\mathbf{k}}\end{bmatrix}\begin{bmatrix}\boldsymbol{\beta}_{\mathbf{k}}\\ \boldsymbol{\beta}^\dagger_{-\mathbf{k}}\end{bmatrix}, \\
&= \sqrt{2\gamma M_{\mathrm{s}}/d}\,\hat{\sigma}^+_{-\mathbf{k}}\bar{\mathbf{f}}_k(x) \\
&\quad\times\left[\frac{(1+\sin\phi_{\mathbf{k}})}{2}\mathbf{T}^{pp}_{\mathbf{k}} + \frac{(1-\sin\phi_{\mathbf{k}})}{2}\mathbf{T}^{np}_{\mathbf{k}} \quad \frac{(1+\sin\phi_{\mathbf{k}})}{2}\mathbf{T}^{pn}_{\mathbf{k}} + \frac{(1-\sin\phi_{\mathbf{k}})}{2}\mathbf{T}^{nn}_{\mathbf{k}}\right]\begin{bmatrix}\boldsymbol{\beta}_{\mathbf{k}}\\ \boldsymbol{\beta}^\dagger_{-\mathbf{k}}\end{bmatrix}, \\
&= \sqrt{2\gamma M_{\mathrm{s}}/d}\,\hat{\sigma}^+_{-\mathbf{k}}\bar{\mathbf{f}}_k(x)\left(\frac{(1+\sin\phi_{\mathbf{k}})}{2}\mathbf{T}^{pp}_{\mathbf{k}} + \frac{(1-\sin\phi_{\mathbf{k}})}{2}\mathbf{T}^{np}_{\mathbf{k}}\right)\boldsymbol{\beta}_{\mathbf{k}} + (\text{H.c. \& } \mathbf{k}\to-\mathbf{k}),
\end{aligned} \tag{S228}$$

where in the last line we used Eq. (S203). This results in

$$\begin{aligned}
\mathbf{h}(\mathbf{r}) &= \sqrt{2\gamma M_{\mathrm{s}}/d}\int\frac{d\mathbf{k}}{(2\pi)^2}e^{i\mathbf{k}\cdot\boldsymbol{\rho}}\,\hat{\sigma}^+_{-\mathbf{k}}\bar{\mathbf{f}}_k(x)\left(\frac{(1+\sin\phi_{\mathbf{k}})}{2}\mathbf{T}^{pp}_{\mathbf{k}} + \frac{(1-\sin\phi_{\mathbf{k}})}{2}\mathbf{T}^{np}_{\mathbf{k}}\right)\boldsymbol{\beta}_{\mathbf{k}} + \text{H.c.}, \\
&= \frac{\sqrt{2\omega_M\omega_d}}{\gamma\mu_0 d}\int\frac{d\mathbf{q}}{(2\pi)^2}e^{i\mathbf{k}\cdot\boldsymbol{\rho}}\,\hat{\sigma}^+_{-\mathbf{k}}\bar{\mathbf{f}}_k(x)\left(\frac{(1+\sin\phi_{\mathbf{k}})}{2}\mathbf{T}^{pp}_{\mathbf{k}} + \frac{(1-\sin\phi_{\mathbf{k}})}{2}\mathbf{T}^{np}_{\mathbf{k}}\right)\boldsymbol{\beta}_{\mathbf{k}} + \text{H.c.},
\end{aligned} \tag{S229}$$

where we defined $\omega_d = \mu_0\gamma^2/d^3$ and $\mathbf{q} = \mathbf{k}d$. Substituting Eqs. (S229) and (S218) into Eq. (S166), and using the rotation-wave approximation, we obtain

$$\begin{aligned}
\mathcal{H}_{\mathrm{int}} &= \gamma\mu_0\mathbf{S}_{\mathrm{NV}}\cdot\mathbf{h}(\mathbf{r})|_{\mathbf{r}=\mathbf{r}_0}, \\
&\approx \frac{\sqrt{\omega_M\omega_d}}{d}\int\frac{d\mathbf{q}}{(2\pi)^2}\,(1+\sin\phi_{\mathbf{k}})\bar{\mathbf{f}}_k(x_0)\left(\frac{(1+\sin\phi_{\mathbf{k}})}{2}\mathbf{T}^{pp}_{\mathbf{k}} + \frac{(1-\sin\phi_{\mathbf{k}})}{2}\mathbf{T}^{np}_{\mathbf{k}}\right)\boldsymbol{\beta}_{\mathbf{k}}e^{i\mathbf{k}\cdot\boldsymbol{\rho}_0}\sigma_+ + \text{H.c.}, \\
&= \sum_{n=0}^{N}\int\frac{d\mathbf{k}}{(2\pi)^2}g_{\mathbf{k},n}\beta_{\mathbf{k},n}e^{i\mathbf{k}\cdot\boldsymbol{\rho}_0}\sigma_+ + \text{H.c.},
\end{aligned} \tag{S230}$$

where in the second line we used $\mathbb{P}\mathbf{S}_{\rm NV}\mathbb{P} = (\hat{e}_-\sigma_- + \hat{e}_+\sigma_+)/\sqrt{2} - \hat{z}|e\rangle\langle e|$ with the projection operator $\mathbb{P}$ onto the NV center's lowest two energy subspace spanned by $|g\rangle = |m_s = 0\rangle$ and $|e\rangle = |m_s = -1\rangle$, and we kept the terms that only act within this subspace of interest. In the third line, we defined the NV-magnon coupling

$$g_{\mathbf{k},n} \;=\; d\sqrt{\omega_M\omega_d}(1+\sin\phi_{\mathbf{k}})\left[\bar{\mathbf{f}}_k(x_0)\left(\frac{(1+\sin\phi_{\mathbf{k}})}{2}\mathbf{T}^{pp}_{\mathbf{k}} + \frac{(1-\sin\phi_{\mathbf{k}})}{2}\mathbf{T}^{np}_{\mathbf{k}}\right)\right]_n, \quad \text{(S231)}$$

where $[\cdots]_n$ indicates the $(n+1)$-th entry of the $1 \times (N+1)$ matrix with $n = 0, 1, \cdots, N$. The above NV-magnon coupling $g_{\mathbf{k},n}$ has the correct unit of $(\text{frequency}) \times (\text{Length})$.

We note that the above identification of $\mathbb{P}S^-_{\rm NV}\mathbb{P} = \sqrt{2}\sigma_+$ is not valid when the external magnetic field is not parallel to the NV axis. In such cases, we firstly calculate the lowest two energy eigenstates by diagonalizing the NV Hamiltonian to obtain $|g\rangle$ and $|e\rangle$. Then, we compute a three-dimensional vector $\vec{a}$ defined by

$$\vec{a} = \sqrt{2}\langle e|\mathbf{S}_{\rm NV}|g\rangle. \quad \text{(S232)}$$

Note that this vector reduces to $\vec{a} = \hat{e}_+$ when the external magnetic field is parallel to the NV axis. In terms of this vector, we obtain $\mathbb{P}\mathbf{S}_{\rm NV}\mathbb{P} = (\vec{a}^*\sigma_- + \vec{a}\sigma_+)/\sqrt{2} + (\text{diagonal terms})$, where (diagonal terms) indicates the terms diagonal in the NV-spin energy eigenstates. Finally, following the above derivation, we find that the NV-magnon coupling $g_{\mathbf{k},n}$ is proportional to the coefficient of $\vec{a}\cdot\mathbf{h}(\mathbf{r} = \mathbf{r}_0)$ accompanying the magnon annihilation operators $\beta_{\mathbf{k},n}$. More concretely, we obtain

$$g_{\mathbf{k},n} = \gamma\mu_0\left[\langle e|\mathbf{S}_{\rm NV}|g\rangle\cdot\mathbf{h}(\mathbf{r} = \mathbf{r}_0), \beta^\dagger_{\mathbf{k},n}\right] = \frac{\gamma\mu_0}{\sqrt{2}}\left[\vec{a}\cdot\mathbf{h}(\mathbf{r} = \mathbf{r}_0), \beta^\dagger_{\mathbf{k},n}\right], \quad \text{(S233)}$$

where $\mathbf{h}(\mathbf{r})$ is provided in Eq. (S229). As we are considering terms up to linear order in the magnon creation and annihilation operators, the commutator with $\beta^\dagger_{\mathbf{k},n}$ simply indicates taking the coefficient of $\mathbf{h}_{\mathbf{k}}(x)$ accompanying $\beta_{\mathbf{k},n}$ due to the commutation relation $[\beta_{\mathbf{k},n}, \beta^\dagger_{\mathbf{k}',n'}] = (2\pi)^2\delta(\mathbf{k} - \mathbf{k}')\delta_{n,n'}$.

SIII.4 Calculation of the T_1 relaxation rate from the coupling

To obtain the longitudinal (T_1) relaxation rate from the NV-magnon coupling strength, we use the results from the derivation of the Lindblad master equation (Sec. SIII.6). Based on Eq. (S297), we

obtain

$$
\begin{aligned}
1/T_1 &= \coth(\beta\omega_s/2)\sum_j |g_j|^2\, 2\pi\delta\left(\omega_s-\omega_j\right), \\
&= \coth(\beta\omega_{\rm NV}/2)\sum_{n=0}^{N}\int \frac{d\mathbf{k}}{(2\pi)^2}|g_{\mathbf{k},n}|^2 2\pi\delta(\omega_{\mathbf{k},n}-\omega_{\rm NV}), \qquad \text{(S234)}
\end{aligned}
$$

where β is the inverse temperature, $\omega_s = \omega_{\rm NV}$ is the NV frequency, $j = (\mathbf{k}, n)$ is the magnon label, $\omega_j = \omega_{\mathbf{k},n}$ is the magnon frequency with label $(\mathbf{k}, n)$, and we replaced $\sum_j \to L^2 \sum_n \int d\mathbf{k}/(2\pi)^2$ and $g_j \to L^{-1} g_{\mathbf{k},n}$. This result is provided in Eq. (S170). For the cutoff thickness mode N in the case $d = 3\ \mu\mathrm{m}$, we used $N + 1 = 55$. This is selected such that $D_{\rm ex}\kappa_N^2 = D_{\rm ex}(N\pi/d)^2 \lesssim 5$ GHz, which is enough to construct magnon modes interacting with NV centers, as the NV frequency is $\omega_{\rm NV} < 3$ GHz. In Fig. S18, we show the resulting T_1 decay rates with respect to the number of branches, $N + 1$, to show the convergence with respect to N. In the inset of Fig. S18, the error is evaluated by $(\mathrm{Error}) = |v_{N+1} - v_{N+1=55}|/|v_{N+1=55}|$, where v_{N+1} is the vector containing the result of T_1 relaxation rates under a given number of branches $(N+1)$ and the size of the vector is evaluated by $|\cdots| = \sqrt{(l^2\mathrm{-norm})} = \sqrt{\sum_i(\cdots)_i^2}$.

SIII.5 Equivalence to the white-noise driven magnons

In Ref. (*11*), another approach for the calculation of the magnon-induced longitudinal (T_1) relaxation is successfully introduced, where the magnons are assumed to be driven by white-noise. This, in turn, is in contrast with our initial approach where the magnons distribution is assumed to follow the statistical mechanics for bosons (thermal occupation of the magnons). Here, we show the two approaches are equivalent in the limit where the Gilbert damping parameter α is small. To show the equivalence, we extend the approach used in Ref. (*11*) to the case where the ferromagnetic film thickness is not small.

To consider the white-noise magnetic field driving magnons, we introduce the noise force Hamiltonian:

$$
\mathcal{H}_{\rm d} = -\mu_0 \int d\mathbf{r}\boldsymbol{\zeta}(\mathbf{r},t)\cdot\mathbf{m}(\mathbf{r}), \qquad \text{(S235)}
$$

where $\mathbf{m}(\mathbf{r}) = \sqrt{2\gamma M_{\rm s}(x)}(\hat{e}_+ a(\mathbf{r}) + \hat{e}_- a^*(\mathbf{r}))/2 \approx M_x(\mathbf{r})\hat{x} + M_y(\mathbf{r})\hat{y}$ is the linearized magnetization oscillation and the white noise magnetic field $\boldsymbol{\zeta}(\mathbf{r}, t)$ satisfies

$$\overline{\zeta_i(\mathbf{r}, t')\zeta_j(\mathbf{r}', t)} = 2D_{\rm th}\delta_{ij}\delta(t - t')\delta(\mathbf{r} - \mathbf{r}'), \tag{S236}$$

where the overline indicates the statistical average, $D_{\rm th} = \alpha k_{\rm B} T/\gamma M_{\rm s}$, and α is the Gilbert damping parameter. Adding the damping term in the Landau-Lifshitz-Gilbert (LLG) equation, the equation of motion for the complex canonical variables becomes

$$i\partial_t \begin{bmatrix} \boldsymbol{\alpha}_{\mathbf{k}}(t) \\ \boldsymbol{\alpha}^\dagger_{-\mathbf{k}}(t) \end{bmatrix} = \begin{bmatrix} 1 & 0 \\ 0 & -1 \end{bmatrix} \left((\mathbf{H}_{\mathbf{k}} + \alpha\partial_t) \cdot \begin{bmatrix} \boldsymbol{\alpha}_{\mathbf{k}}(t) \\ \boldsymbol{\alpha}^\dagger_{-\mathbf{k}}(t) \end{bmatrix} + \begin{bmatrix} \mathbb{Y}^-_{+\mathbf{k}}(t) \\ \mathbb{Y}^+_{+\mathbf{k}}(t) \end{bmatrix} \right), \tag{S237}$$

where $\mathbf{H}_{\mathbf{k}}$ is the same matrix appearing in Eq. (S190) and we defined

$$\begin{bmatrix} \mathbb{Y}^-_{+\mathbf{k}}(t) \\ \mathbb{Y}^+_{+\mathbf{k}}(t) \end{bmatrix} = -\frac{\sqrt{2\gamma M_S}}{2} \begin{bmatrix} \mathbb{X}^-_{+\mathbf{k}}(t) \\ \mathbb{X}^+_{+\mathbf{k}}(t) \end{bmatrix}, \tag{S238}$$

$$\mathbb{X}^-_{+\mathbf{k}}(t) \equiv \left[\zeta^-_{+\mathbf{k},0}(t), \zeta^-_{+\mathbf{k},1}(t), \cdots \zeta^-_{+\mathbf{k},N}(t)\right]^T, \tag{S239}$$

$$\mathbb{X}^+_{+\mathbf{k}}(t) \equiv \left[\zeta^+_{+\mathbf{k},0}(t), \zeta^+_{+\mathbf{k},1}(t), \cdots \zeta^+_{+\mathbf{k},N}(t)\right]^T, \tag{S240}$$

$$\zeta^\pm_{+\mathbf{k},n}(t) = \zeta^x_{+\mathbf{k},n}(t) \pm i\zeta^y_{+\mathbf{k},n}(t), \tag{S241}$$

$$\boldsymbol{\zeta}(\mathbf{r}, t) = \int \frac{d\mathbf{k}}{(2\pi)^2} e^{i\mathbf{k}\cdot\boldsymbol{\rho}} \boldsymbol{\zeta}_{\mathbf{k}}(x, t) = \int \frac{d\mathbf{k}}{(2\pi)^2} e^{i\mathbf{k}\cdot\boldsymbol{\rho}} \sum_{n=0}^{N} f_n(x)\boldsymbol{\zeta}_{\mathbf{k},n}(t). \tag{S242}$$

We further define the Fourier transform in time:

$$\begin{bmatrix} \boldsymbol{\alpha}_{\mathbf{k}}(t) \\ \boldsymbol{\alpha}^\dagger_{-\mathbf{k}}(t) \end{bmatrix} = \int \frac{d\omega}{2\pi} e^{-i\omega t} \begin{bmatrix} \boldsymbol{\alpha}_{\mathbf{k}}[\omega] \\ \boldsymbol{\alpha}^\dagger_{-\mathbf{k}}[\omega] \end{bmatrix}, \tag{S243}$$

$$\begin{bmatrix} \mathbb{Y}^-_{+\mathbf{k}}(t) \\ \mathbb{Y}^+_{+\mathbf{k}}(t) \end{bmatrix} = \int \frac{d\omega}{2\pi} e^{-i\omega t} \begin{bmatrix} \mathbb{Y}^-_{+\mathbf{k}}[\omega] \\ \mathbb{Y}^+_{+\mathbf{k}}[\omega] \end{bmatrix}, \tag{S244}$$

which allows us to write the equation of motion as

$$\begin{bmatrix} \boldsymbol{\alpha}_{\mathbf{k}}[\omega] \\ \boldsymbol{\alpha}^+_{-\mathbf{k}}[\omega] \end{bmatrix} = (\omega\sigma_z - \mathbf{H}_{\mathbf{k}} + i\omega\alpha)^{-1} \begin{bmatrix} \mathbb{Y}^-_{+\mathbf{k}}[\omega] \\ \mathbb{Y}^+_{+\mathbf{k}}[\omega] \end{bmatrix} = \mathbf{S}_{\mathbf{k}}[\omega] \begin{bmatrix} \mathbb{Y}^-_{+\mathbf{k}}[\omega] \\ \mathbb{Y}^+_{+\mathbf{k}}[\omega] \end{bmatrix}, \tag{S245}$$

where we defined the response matrix

$$\mathbf{S}_{\mathbf{k}}[\omega] = (\omega\boldsymbol{\sigma}_z - \mathbf{H}_{\mathbf{k}} + i\omega\alpha)^{-1}. \tag{S246}$$

The white-noise property is reduced to the following averaging reration

$$\overline{\begin{bmatrix} \mathbb{Y}^-_{+\mathbf{k}}[\omega] \\ \mathbb{Y}^+_{+\mathbf{k}}[\omega] \end{bmatrix} \begin{bmatrix} \mathbb{Y}^-_{+\mathbf{k}'}[\omega'] \\ \mathbb{Y}^+_{+\mathbf{k}'}[\omega'] \end{bmatrix}^T} = \boldsymbol{\sigma}_x 2\alpha k_B T \times 2\pi\delta(\omega + \omega')(2\pi)^2\delta(\mathbf{k} + \mathbf{k}'). \tag{S247}$$

The time-domain complex canonical variables driven by the white noise are written as

$$\begin{bmatrix} \boldsymbol{\alpha}_{\mathbf{k}}(t) \\ \boldsymbol{\alpha}^{\dagger}_{-\mathbf{k}}(t) \end{bmatrix} = \int \frac{d\omega}{2\pi} e^{-i\omega t} \mathbf{S}_{\mathbf{k}}[\omega] \begin{bmatrix} \mathbb{Y}^{-}_{+\mathbf{k}}[\omega] \\ \mathbb{Y}^{+}_{+\mathbf{k}}[\omega] \end{bmatrix}. \tag{S248}$$

From Eq. (S224), the circular components of the magnetic field generated by magnons are given by

$$h^{+}_{\mathbf{k}}(x,t) = \sqrt{2\gamma M_s/d}\,(1+\sin\phi_{\mathbf{k}})\,\mathbf{w}_{\mathbf{k}} \cdot \begin{bmatrix} \boldsymbol{\alpha}_{\mathbf{k}}(t) \\ \boldsymbol{\alpha}^{\dagger}_{-\mathbf{k}}(t) \end{bmatrix}, \tag{S249}$$

$$h^{-}_{\mathbf{k}}(x,t) = \sqrt{2\gamma M_s/d}\,(1-\sin\phi_{\mathbf{k}})\,\mathbf{w}_{\mathbf{k}} \cdot \begin{bmatrix} \boldsymbol{\alpha}_{\mathbf{k}}(t) \\ \boldsymbol{\alpha}^{\dagger}_{-\mathbf{k}}(t) \end{bmatrix}. \tag{S250}$$

Therefore, the noise correlation is

$$\begin{aligned} \overline{h^{+}(\mathbf{r},t)h^{-}(\mathbf{r},t')} &= \int \frac{d\mathbf{k}}{(2\pi)^2} \int \frac{d\mathbf{k}'}{(2\pi)^2} e^{i(\mathbf{k}+\mathbf{k}')\cdot\rho} \overline{h^{+}_{\mathbf{k}}(x,t) h^{-}_{\mathbf{k}'}(x,t')}, \\ &= (2\gamma M_s/d) \int \frac{d\omega}{2\pi} e^{-i\omega(t-t')} \int \frac{d\mathbf{k}}{(2\pi)^2} (1+\sin\phi_{\mathbf{k}})^2\, \mathbf{w}_{\mathbf{k}} \cdot \left(2\alpha k_B T \mathbf{S}_{\mathbf{k}}[\omega]\,(\boldsymbol{\sigma}_x)\,\mathbf{S}^{T}_{-\mathbf{k}}[-\omega]\right) \cdot \mathbf{w}^{T}_{-\mathbf{k}}, \\ &= (2\gamma M_s/d) \int \frac{d\omega}{2\pi} e^{-i\omega(t-t')} \int \frac{d\mathbf{k}}{(2\pi)^2} (1+\sin\phi_{\mathbf{k}})^2\, \mathbf{w}_{\mathbf{k}} \cdot \mathbf{S}_{\mathbf{k}}[\omega]\,(2\alpha k_B T)\,\mathbf{S}^{\dagger}_{\mathbf{k}}[\omega] \cdot \mathbf{w}^{T}_{\mathbf{k}}, \end{aligned} \tag{S251}$$

where we used $\mathbf{w}_{-\mathbf{k}} = \mathbf{w}_{\mathbf{k}}\boldsymbol{\sigma}_x$ and $\mathbf{S}^{T}_{-\mathbf{k}}[-\omega] = \boldsymbol{\sigma}_x \mathbf{S}^{\dagger}_{\mathbf{k}}[\omega]\boldsymbol{\sigma}_x$. Longitudinal relaxation rate is $1/T_1 = 2\Gamma(\omega_{\mathrm{NV}})$ with

$$\begin{aligned} \Gamma(\omega) &= \frac{(\gamma\mu_0)^2}{2} \int dt e^{i\omega t} \overline{h^{+}(\mathbf{r},t)h^{-}(\mathbf{r},0)}, \\ &= \omega_M \omega_d \int \frac{d\mathbf{q}}{(2\pi)^2} (1+\sin\phi_{\mathbf{k}})^2\, \mathbf{w}_{\mathbf{k}} \cdot \mathbf{S}_{\mathbf{k}}[\omega]\,(2\alpha k_B T)\,\mathbf{S}^{\dagger}_{\mathbf{k}}[\omega] \cdot \mathbf{w}^{T}_{\mathbf{k}}, \\ &= \omega_M \omega_d \int \frac{d\mathbf{q}}{(2\pi)^2} (1+\sin\phi_{\mathbf{k}})^2\, \mathbf{w}_{\mathbf{k}} \cdot [\omega\boldsymbol{\sigma}_z - \mathbf{H}_{\mathbf{k}} + i\alpha\omega]^{-1}\,(2\alpha k_B T)\left([\omega\boldsymbol{\sigma}_z - \mathbf{H}_{\mathbf{k}} + i\alpha\omega]^{-1}\right)^{\dagger} \cdot \mathbf{w}^{T}_{\mathbf{k}}. \end{aligned} \tag{S252}$$

We note the factor of two difference between $1/T_1$ and $\Gamma(\omega_{\mathrm{NV}})$. This is because Γ is one-way decay rate for $|g\rangle \to |e\rangle$ or $|e\rangle \to |g\rangle$, while in this work $1/T_1$ is chosen to be the decay rate of form $\langle\sigma_z(t)\rangle/\langle\sigma_z(0)\rangle = e^{-t/T_1}$, or more in general $(\langle\sigma_z(t)\rangle - \langle\sigma_z(\infty)\rangle)/(\langle\sigma_z(0) - \langle\sigma_z(\infty)\rangle) = e^{-t/T_1}$ where at high temperature $\langle\sigma_z(t=\infty)\rangle = 0$. Extra attention is needed when comparing our results and values reported in other publications.

In Fig. S19, we numerically compute the T_1 relaxation rate with Eqs. (S170) and (S252). For $\alpha \ll 1$, two approaches appears equivalent. In the following, we show that these two are approximately the

same in the limit $\alpha \ll 1$ and high temperature. To this end, we consider a part of Eq. (S252) defined as $\mathbf{\Lambda}_{\mathbf{k}}(\omega)$:

$$\begin{aligned}
&\mathbf{\Lambda}_{\mathbf{k}}(\omega) = \left[\omega\boldsymbol{\sigma}_z - \mathbf{H}_{\mathbf{k}} + i\alpha\omega\right]^{-1}(2\alpha k_B T)\left(\left[\omega\boldsymbol{\sigma}_z - \mathbf{H}_{\mathbf{k}} + i\alpha\omega\right]^{-1}\right)^{\dagger}, \\
&= (k_B T)\,\mathbf{T}_{\mathbf{k}}\left[\omega\boldsymbol{\sigma}_z - \boldsymbol{\mathcal{E}}_{\mathbf{k}} + i\alpha\omega\mathbf{T}_{\mathbf{k}}^{\dagger}\mathbf{T}_{\mathbf{k}}\right]^{-1}\left(2\alpha\mathbf{T}_{\mathbf{k}}^{\dagger}\mathbf{T}_{\mathbf{k}}\right)\left(\left[\omega\boldsymbol{\sigma}_z - \boldsymbol{\mathcal{E}}_{\mathbf{k}} + i\alpha\omega\mathbf{T}_{\mathbf{k}}^{\dagger}\mathbf{T}_{\mathbf{k}}\right]^{-1}\right)^{\dagger}\mathbf{T}_{\mathbf{k}}^{\dagger},
\end{aligned} \tag{S253}$$

where $\mathbf{T}_{\mathbf{k}}$ is the paraunitary matrix. When $\alpha \to 0$, eigenvalues of the matrix $\left(\omega\boldsymbol{\sigma}_z - \boldsymbol{\mathcal{E}}_{\mathbf{k}} + i\alpha\omega\mathbf{T}_{\mathbf{k}}^{\dagger}\mathbf{T}_{\mathbf{k}}\right)$ are approximately those of $\left(\omega\boldsymbol{\sigma}_z - \boldsymbol{\mathcal{E}}_{\mathbf{k}} + i\alpha\omega[\mathbf{T}_{\mathbf{k}}^{\dagger}\mathbf{T}_{\mathbf{k}}]_{\mathrm{diag}}\right)$, where $[\cdots]_{\mathrm{diag}}$ represents the diagonal part of the matrix. Therefore, we approximate

$$\begin{aligned}
\left[\omega\boldsymbol{\sigma}_z - \boldsymbol{\mathcal{E}}_{\mathbf{k}} + i\alpha\omega\mathbf{T}_{\mathbf{k}}^{\dagger}\mathbf{T}_{\mathbf{k}}\right]^{-1} &\approx \left[\omega\boldsymbol{\sigma}_z - \boldsymbol{\mathcal{E}}_{\mathbf{k}} + i\alpha\omega\left[\mathbf{T}_{\mathbf{k}}^{\dagger}\mathbf{T}_{\mathbf{k}}\right]_{\mathrm{diag}}\right]^{-1}, \\
&\approx \left[\omega\boldsymbol{\sigma}_z - \boldsymbol{\mathcal{E}}_{\mathbf{k}} + i\alpha\boldsymbol{\sigma}_z\boldsymbol{\mathcal{E}}_{\mathbf{k}}\left[\mathbf{T}_{\mathbf{k}}^{\dagger}\mathbf{T}_{\mathbf{k}}\right]_{\mathrm{diag}}\right]^{-1}, \\
&= \boldsymbol{\sigma}_z\left[\omega - \boldsymbol{\sigma}_z\boldsymbol{\mathcal{E}}_{\mathbf{k}} + i\alpha\boldsymbol{\mathcal{E}}_{\mathbf{k}}\left[\mathbf{T}_{\mathbf{k}}^{\dagger}\mathbf{T}_{\mathbf{k}}\right]_{\mathrm{diag}}\right]^{-1},
\end{aligned} \tag{S254}$$

where in the second line, we approximated $i\alpha\omega \approx i\alpha\boldsymbol{\sigma}_z\boldsymbol{\mathcal{E}}_{\mathbf{k}}$ as the poles are at $\omega \approx \boldsymbol{\sigma}_z\boldsymbol{\mathcal{E}}_{\mathbf{k}}$. Furthermore, assuming the entries of the diagonal matrix $\boldsymbol{\sigma}_z\boldsymbol{\mathcal{E}}_{\mathbf{k}}$ are not degenerate, we obtain

$$\begin{aligned}
&\boldsymbol{\sigma}_z\left[\omega - \boldsymbol{\sigma}_z\boldsymbol{\mathcal{E}}_{\mathbf{k}} + i\alpha\boldsymbol{\mathcal{E}}_{\mathbf{k}}\left[\mathbf{T}_{\mathbf{k}}^{\dagger}\mathbf{T}_{\mathbf{k}}\right]_{\mathrm{diag}}\right]^{-1}(2\alpha\mathbf{T}_{\mathbf{k}}^{\dagger}\mathbf{T}_{\mathbf{k}})\left(\left[\omega - \boldsymbol{\sigma}_z\boldsymbol{\mathcal{E}}_{\mathbf{k}} + i\alpha\boldsymbol{\mathcal{E}}_{\mathbf{k}}\left[\mathbf{T}_{\mathbf{k}}^{\dagger}\mathbf{T}_{\mathbf{k}}\right]_{\mathrm{diag}}\right]^{-1}\right)^{\dagger}\boldsymbol{\sigma}_z \\
&\approx 2\pi\delta\left(\omega - \boldsymbol{\sigma}_z\boldsymbol{\mathcal{E}}_{\mathbf{k}}\right)\boldsymbol{\mathcal{E}}_{\mathbf{k}}^{-1},
\end{aligned} \tag{S255}$$

where the delta-function with matrix argument is defined as

$$\delta(\omega - \boldsymbol{\sigma}_z\boldsymbol{\mathcal{E}}_{\mathbf{k}}) = \mathrm{diag}[\delta(\omega - \omega_{\mathbf{k},0}), \cdots, \delta(\omega - \omega_{\mathbf{k},N}), \delta(\omega + \omega_{-\mathbf{k},0}), \cdots, \delta(\omega + \omega_{-\mathbf{k},N})]. \tag{S256}$$

With these approximations for $\alpha \to 0$, we obtain

$$\mathbf{\Lambda}_{\mathbf{k}}(\omega) \approx (k_B T)\,\mathbf{T}_{\mathbf{k}}\left[2\pi\delta\left(\omega - \boldsymbol{\sigma}_z\varepsilon_{\mathbf{k}}\right)\boldsymbol{\mathcal{E}}_{\mathbf{k}}^{-1}\right]\mathbf{T}_{\mathbf{k}}^{\dagger} = \frac{(k_B T)}{\omega}\mathbf{T}_{\mathbf{k}}\left[2\pi\delta\left(\omega - \boldsymbol{\sigma}_z\boldsymbol{\mathcal{E}}_{\mathbf{k}}\right)\right]\boldsymbol{\sigma}_z\mathbf{T}_{\mathbf{k}}^{\dagger}. \tag{S257}$$

Therefore, the one-way longitudinal relaxation $\Gamma(\omega)$ becomes

$$\begin{aligned}
\Gamma(\omega) &= \omega_M\omega_d\int\frac{d\mathbf{q}}{(2\pi)^2}\left(1 + \sin\phi_{\mathbf{k}}\right)^2\mathbf{w}_{\mathbf{k}}\cdot\mathbf{\Lambda}_{\mathbf{k}}(\omega)\cdot\mathbf{w}_{\mathbf{k}}^T, \\
&\approx \frac{k_B T}{\omega}\cdot\omega_M\omega_d\int\frac{d\mathbf{q}}{(2\pi)^2}\left(1 + \sin\phi_{\mathbf{k}}\right)^2\mathbf{w}_{\mathbf{k}}\mathbf{T}_{\mathbf{k}}\left[2\pi\delta\left(\omega - \boldsymbol{\sigma}_z\mathcal{E}_{\mathbf{k}}\right)\right]\boldsymbol{\sigma}_z\left(\mathbf{w}_{\mathbf{k}}\mathbf{T}_{\mathbf{k}}\right)^{\dagger}.
\end{aligned} \tag{S258}$$

The full longitudinal relaxation rate $1/T_1$ is obtained by substituting $\omega = \omega_{\rm NV} > 0$ into $\Gamma(\omega)$ and multiplied by two

$$
\begin{aligned}
&1/T_1 = 2\Gamma(\omega_{\rm NV}),\\
&\approx \frac{2k_BT}{\omega_{\rm NV}}\cdot\omega_M\omega_d\int\frac{d\mathbf{q}}{(2\pi)^2}\left(1+\sin\phi_{\mathbf{k}}\right)^2\mathbf{w}_{\mathbf{k}}\mathbf{T}_{\mathbf{k}}\begin{bmatrix}2\pi\delta\left(\omega_{\rm NV}-\mathbf{E}_{\mathbf{k}}\right) & \mathbf{O}\\ \mathbf{O} & 2\pi\delta\left(\omega_{\rm NV}+\mathbf{E}_{-\mathbf{k}}\right)\end{bmatrix}\left(\mathbf{w}_{\mathbf{k}}\mathbf{T}_{\mathbf{k}}\right)^{\dagger},
\end{aligned}
\tag{S259}
$$

where the right-bottom sector of the matrix in Eq. (S259) is always zero because the arguments of the entries of the matrix delta-function are all positive. Taking advantage of the explicit form:

$$
\begin{aligned}
&\mathbf{w}_{\mathbf{k}}\mathbf{T}_{\mathbf{k}} = (1/2)\left[(1+\sin\phi_{\mathbf{k}})\,\overline{\mathbf{f}}_k(x)\quad(1-\sin\phi_{\mathbf{k}})\,\overline{\mathbf{f}}_k(x)\right]\begin{bmatrix}\mathbf{T}^{pp}_{\mathbf{k}} & \mathbf{T}^{pn}_{\mathbf{k}}\\ \mathbf{T}^{np}_{\mathbf{k}} & \mathbf{T}^{nn}_{\mathbf{k}}\end{bmatrix},\\
&= \left[\overline{\mathbf{f}}_k(x)\left((1+\sin\phi_{\mathbf{k}})\,\mathbf{T}^{pp}_{\mathbf{k}}+(1-\sin\phi_{\mathbf{k}})\,\mathbf{T}^{np}_{\mathbf{k}}\right)\quad\overline{\mathbf{f}}_k(x)\left((1+\sin\phi_{\mathbf{k}})\,\overline{\mathbf{f}}_k(x)\mathbf{T}^{pn}_{\mathbf{k}}+(1-\sin\phi_{\mathbf{k}})\,\mathbf{T}^{nn}_{\mathbf{k}}\right)\right]\\
&\quad\times(1/2),
\end{aligned}
\tag{S260}
$$

we obtain from Eqs. (S259) and (S231):

$$
1/T_1 \;=\; \frac{2k_BT}{\omega_{\rm NV}}\sum_{n=0}^{N}\int\frac{d\mathbf{k}}{(2\pi)^2}\left|g_{\mathbf{k},n}\right|^2 2\pi\delta\left(\omega_{\rm NV}-\omega_{\mathbf{k},n}\right),
\tag{S261}
$$

which shows that the two approaches are equivalent after noticing $2k_{\rm B}T/\omega_{\rm NV}\approx\coth(\beta\omega_{\rm NV}/2)$.

SIII.5.1 Relationship between the paraunitary matrix and the linewidth

From Eq. (S254), we observe that the linewidth of the magnon modes depends on the paraunitary matrix $\mathbf{T}_{\mathbf{k}}$. This is because the poles are at

$$
\omega \approx \left[\boldsymbol{\sigma}_z\boldsymbol{\mathcal{E}}_{\mathbf{k}} - i\alpha\boldsymbol{\mathcal{E}}[\mathbf{T}^{\dagger}_{\mathbf{k}}\mathbf{T}_{\mathbf{k}}]_{\rm diag}\right]_n.
\tag{S262}
$$

On the other hand, from Eq. (S245), the damping term in the equation of motion can be regarded as the change in external field $\mathbf{H}_{\mathbf{k}} - i\omega\alpha = \mathbf{H}_{\mathbf{k}}|_{\omega_H\to\omega_H-i\omega\alpha}$, as is found in Ref. (*35*), leading to an alternative expression of the poles:

$$
\omega = \pm\left(\omega_{\mathbf{k},n} - i\alpha\omega\frac{\partial\omega_{\mathbf{k},n}}{\partial\omega_H}\right) \approx \pm\omega_{\mathbf{k},n} - i\alpha\omega_{\mathbf{k},n}\frac{\partial\omega_{\mathbf{k},n}}{\partial\omega_H}.
\tag{S263}
$$

Eqs. (S262) and (S263) are equivalent because one can show

$$\left[\mathbf{T}_{\mathbf{k}}^{\dagger}\mathbf{T}_{\mathbf{k}}\right]_{\mathrm{diag}} = \frac{\partial \boldsymbol{\mathcal{E}}_{\mathbf{k}}}{\partial \omega_H}. \tag{S264}$$

This is because

$$\frac{\partial \boldsymbol{\mathcal{E}}_{\mathbf{k}}}{\partial \omega_H} = \left[\frac{\partial \boldsymbol{\mathcal{E}}_{\mathbf{k}}}{\partial \omega_H}\right]_{\mathrm{diag}} = \frac{\partial \left[\mathbf{T}_{\mathbf{k}}^{\dagger}\mathbf{H}_{\mathbf{k}}\mathbf{T}_{\mathbf{k}}\right]_{\mathrm{diag}}}{\partial \omega_H} = \left[\mathbf{T}_{\mathbf{k}}^{\dagger}\frac{\partial \mathbf{H}_{\mathbf{k}}}{\partial \omega_H}\mathbf{T}_{\mathbf{k}}\right]_{\mathrm{diag}} + \left[\mathbf{T}_{\mathbf{k}}^{\dagger}\mathbf{H}_{\mathbf{k}}\frac{\partial \mathbf{T}_{\mathbf{k}}}{\partial \omega_H} + \frac{\partial \mathbf{T}_{\mathbf{k}}^{\dagger}}{\partial \omega_H}\mathbf{H}_{\mathbf{k}}\mathbf{T}_{\mathbf{k}}\right]_{\mathrm{diag}}, \tag{S265}$$

and the second term in the last line of Eq. (S265) vanishes:

$$\begin{aligned}\left[\mathbf{T}_{\mathbf{k}}^{\dagger}\mathbf{H}_{\mathbf{k}}\frac{\partial \mathbf{T}_{\mathbf{k}}}{\partial \omega_H} + \frac{\partial \mathbf{T}_{\mathbf{k}}^{\dagger}}{\partial \omega_H}\mathbf{H}_{\mathbf{k}}\mathbf{T}_{\mathbf{k}}\right]_{\mathrm{diag}} &= \left[\mathbf{T}_{\mathbf{k}}^{\dagger}\mathbf{H}_{\mathbf{k}}\frac{\partial \mathbf{T}_{\mathbf{k}}}{\partial \omega_H} + \mathrm{H.c.}\right]_{\mathrm{diag}}, \\ &= \left[\boldsymbol{\mathcal{E}}_{\mathbf{k}}\mathbf{T}_{\mathbf{k}}^{-1}\frac{\partial \mathbf{T}_{\mathbf{k}}}{\partial \omega_H} + \mathrm{H.c.}\right]_{\mathrm{diag}} = \boldsymbol{\mathcal{E}}_{\mathbf{k}}\left[\mathbf{T}_{\mathbf{k}}^{-1}\frac{\partial \mathbf{T}_{\mathbf{k}}}{\partial \omega_H} + \mathrm{H.c.}\right]_{\mathrm{diag}} = 0,\end{aligned} \tag{S266}$$

which is because $\left[\mathbf{T}_{\mathbf{k}}^{-1}\frac{\partial \mathbf{T}_{\mathbf{k}}}{\partial \omega_H} + \mathrm{H.c.}\right]_{\mathrm{diag}} = 0$. This can be shown by

$$\begin{aligned}-\left[\mathbf{T}_{\mathbf{k}}^{-1}\frac{\partial \mathbf{T}_{\mathbf{k}}}{\partial \omega_H} + \mathrm{H.c.}\right]_{\mathrm{diag}} &= \left[\frac{\partial \mathbf{T}_{\mathbf{k}}^{-1}}{\partial \omega_H}\mathbf{T}_{\mathbf{k}} + \mathrm{H.c.}\right]_{\mathrm{diag}} = \left[\frac{\partial \mathbf{T}_{\mathbf{k}}^{-1}}{\partial \omega_H}\mathbf{T}_{\mathbf{k}} + \mathrm{H.c.}\right]_{\mathrm{diag}}^{\dagger}, \\ &= \left[\mathbf{T}_{\mathbf{k}}^{\dagger}\frac{\partial (\mathbf{T}_{\mathbf{k}}^{-1})^{\dagger}}{\partial \omega_H} + \mathrm{H.c.}\right]_{\mathrm{diag}} = \left[\mathbf{T}_{\mathbf{k}}^{\dagger}\frac{\partial (\boldsymbol{\sigma}_z\mathbf{T}_{\mathbf{k}}^{\dagger}\boldsymbol{\sigma}_z)^{\dagger}}{\partial \omega_H} + \mathrm{H.c.}\right]_{\mathrm{diag}} = \left[\mathbf{T}_{\mathbf{k}}^{\dagger}\boldsymbol{\sigma}_z\frac{\partial \mathbf{T}_{\mathbf{k}}}{\partial \omega_H}\boldsymbol{\sigma}_z + \mathrm{H.c.}\right]_{\mathrm{diag}}, \\ &= \left[\boldsymbol{\sigma}_z\mathbf{T}_{\mathbf{k}}^{-1}\frac{\partial \mathbf{T}_{\mathbf{k}}}{\partial \omega_H}\boldsymbol{\sigma}_z + \mathrm{H.c.}\right]_{\mathrm{diag}} = \left[\mathbf{T}_{\mathbf{k}}^{-1}\frac{\partial \mathbf{T}_{\mathbf{k}}}{\partial \omega_H} + \mathrm{H.c.}\right]_{\mathrm{diag}}.\end{aligned} \tag{S267}$$

Combining the first line and the last line, we successfully find $\left[\mathbf{T}_{\mathbf{k}}^{-1}\frac{\partial \mathbf{T}_{\mathbf{k}}}{\partial \omega_H} + \mathrm{H.c.}\right]_{\mathrm{diag}} = 0$.

SIII.6 Derivation of the longitudinal relaxation rate by Lindblad master equation formalism

We derive the master equation of a qubit (NV center) coupled to bosons (magnons) to obtain the self-energy shift and longitudinal relaxation terms of NV centers under the Born-Markov approximation (*36*). Note that we use the term self-energy for both $\Sigma[\omega]$ and $\Sigma[\omega = \omega_{\mathrm{s}}]$ when there is no confusion, where $\Sigma[\omega]$ is the self-energy function or simply the self-energy. More concretely,

for a qubit with frequency ω_s interacting with magnons, the self-energy $\Sigma[\omega]$ is typically defined as $\Sigma[\omega] = G_0^{-1}[\omega] - G^{-1}[\omega]$, where $G_0[\omega] = 1/(\omega - \omega_\mathrm{s} + i0^+)$ and $G[\omega]$ are the bare and dressed Green's function of the qubit, respectively. In our paper, $\Sigma[\omega]$ and $\Sigma[\omega = \omega_\mathrm{s}]$ are both called the self-energy without further note on the function argument, while the real part of $\Sigma[\omega = \omega_\mathrm{s}]$ is called the self-energy shift to distinguish it from the self-energy (which is complex) as it shifts the qubit frequency following $\omega_\mathrm{s} \to \omega_\mathrm{s} + \mathrm{Re}\Sigma[\omega = \omega_\mathrm{s}]$. We also call $\chi[\omega] = -\Sigma[\omega]$ and $\chi[\omega_\mathrm{s}] = -\Sigma[\omega_\mathrm{s}]$ as the self-energy when the sign convention is not the focus. For this case, the qubit frequency shifts $\omega_\mathrm{s} \to \omega_\mathrm{s} - \mathrm{Re}\chi[\omega_\mathrm{s}]$, where $\chi[\omega]$ is more accurately called the susceptibility of the qubit. Our starting point is the equation of motion of the qubit reduced density operator ρ for the system initially described by the total Hamiltonian $\mathcal{H} = \mathcal{H}_0 + \mathcal{H}_B + V$, where $\mathcal{H}_0$ is the qubit Hamiltonian, $\mathcal{H}_B$ is the boson bath Hamiltonian, and V is the perturbative qubit-bath interaction Hamiltonian. Under the Born-Markov approximation with a stationary bath density operator R_0, i.e. $[\mathcal{H}_B, R_0] = 0$, and assuming that the mean effect of the bath on the qubit is zero $\langle V \rangle = 0$, where $\langle \cdots \rangle = \mathrm{Tr}_B[\cdots R_0]$ is the bath expectation and $\mathrm{Tr}_B[\cdots]$ represents the partial trace of the bath states, we obtain (*37*):

$$\frac{d}{dt}\rho(t) = -i\left[\mathcal{H}_0, \rho\right] - \int_{-\infty}^{0} dt' \, \mathrm{Tr}_B\left[V(0), \left[V\left(t'\right), \rho(t)R_0\right]\right], \tag{S268}$$

where the time argument of operators, such as $V(t)$, in this section indicates that the operator is in the interaction picture, except for the density operator $\rho = \rho(t)$. Note that the double commutator part can be decomposed into the Hermitian-like and the non-Hermitian-like terms using the identity for Hermitian operators A, B, and C:

$$[A, [B, C]] = i\left[\frac{i}{2}[B, A], C\right] - \left(ACB + BCA - \frac{1}{2}\{AB, C\} - \frac{1}{2}\{BA, C\}\right), \tag{S269}$$

where $A = V(0)$, $B = V(t')$, and $C = \rho(t)R_0$ in our setting. The first term is Hermitian-like and the second term is non-Hermitian-like. Using the above identity, we obtain

$$\frac{d}{dt}\rho(t) = -i\left[\mathcal{H}_0 + \mathcal{H}_\mathrm{eff}, \rho\right] + \mathcal{N}[\rho(t)], \tag{S270}$$

$$\mathcal{H}_\mathrm{eff} = \frac{i}{2}\int_{-\infty}^{0} dt' \left\langle \left[V\left(t'\right), V(0)\right]\right\rangle, \tag{S271}$$

$$\mathcal{N}[\rho(t)] = \int_{-\infty}^{0} dt' \, \mathrm{Tr}_B\left[V(0)\rho R_0 V\left(t'\right) - \frac{1}{2}\left\{V\left(t'\right)V(0), \rho R_0\right\}\right] + \mathrm{H.c}, \tag{S272}$$

where $\mathcal{H}_{\text{eff}}$ is the effective Hamiltonian and $\mathcal{N}[\rho(t)]$ is the non-Hermitian term. We find that the effective Hamiltonian $\mathcal{H}_{\text{eff}}$ provided in Eq. (S271) is the same as the result of the Schrieffer-Wolff transformation (*38*) except for the bath expectation $\langle \cdots \rangle$, and can be written in terms of the advanced Green's function (*39*)

$$iG^A_{BA}(t) = -\Theta(-t)\left\langle [B(t), A(0)] \right\rangle, \tag{S273}$$

$$G^A_{BA}[\omega] = \int_{-\infty}^{\infty} dt G^A_{BA}(t) e^{i\omega t}, \tag{S274}$$

where $\Theta(\cdots)$ is the Heaviside step function. We can relate this to the system's response property, or the retarded Green's function (*39*):

$$iG^R_{BA}(t) = \Theta(t)\left\langle [B(t), A(0)] \right\rangle, \tag{S275}$$

$$G^R_{BA}[\omega] = \int_{-\infty}^{\infty} dt G^R_{BA}(t) e^{i\omega t}. \tag{S276}$$

If $\langle [B(t), A(0)] \rangle = \langle [B(0), A(-t)] \rangle$ is satisfied, which is true for the stationary bath states, we have the following relationship between the retarded and the advanced Green's function

$$\left(G^A_{BA}[\omega]\right)^* = G^R_{A^\dagger B^\dagger}[\omega], \tag{S277}$$

$$\rightarrow \left(G^A_{BA}[\omega]\right)^* = G^R_{BA}[\omega]; \quad \text{if } B = A^\dagger. \tag{S278}$$

In this work, we define the susceptibility with the following sign convention:

$$\chi_{BA}(t) = -G^R_{BA}(t); \quad \chi_{BA}[\omega] = -G^R_{BA}[\omega], \tag{S279}$$

which is chosen such that the linear response due to an external force $f(t)$ acting on the system via the interaction Hamiltonian $H_{\text{int}} = -f(t)A$ results in the response of the observable B following $\langle \delta B(t) \rangle = \int_{-\infty}^{\infty} d\tau \chi_{BA}(t-\tau) f(\tau)$, where $\langle \delta B(t) \rangle = \langle B(t) \rangle - \langle B \rangle_{\text{eq}}$ is the deviation from the value without the external force (equilibrium value).

Now, we consider one qubit (NV center) coupled to multi-mode bosons (magnons):

$$\mathcal{H} = \mathcal{H}_0 + \mathcal{H}_B + V, \tag{S280}$$

$$\mathcal{H}_0 = \frac{\omega_s}{2}\sigma_z, \tag{S281}$$

$$\mathcal{H}_B = \sum_j \omega_j b_j^\dagger b_j, \tag{S282}$$

$$V = B_-\sigma_+ + B_+\sigma_-;\ \ B_- = \sum_j g_j b_j;\ \ B_+ = (B_-)^\dagger, \tag{S283}$$

where ω_s is the qubit frequency, ω_j is the boson frequency with label j, $b_j^\dagger$ (b_j) is the boson creation (annihilation) operator, g_j is the qubit-boson coupling constant, $\sigma_z = |e\rangle\langle e| - |g\rangle\langle g|$, $\sigma_- = |g\rangle\langle e|$, $\sigma_+ = (\sigma_-)^\dagger$, $|g\rangle$ ($|e\rangle$) is the qubit ground (excited) state, and $B_\pm$ is the operator to represent the boson noise field. Taking the boson density operator R_0 to be in their thermal state $R_0 = Z^{-1}\exp[-\beta\mathcal{H}_B]$, where $\beta = 1/k_\mathrm{B}T$ is the inverse temperature and $Z = \mathrm{Tr}_B[\exp(-\beta\mathcal{H}_B)]$ is the partition function, the effective Hamiltonian becomes

$$\mathcal{H}_\text{eff} = \Sigma_\text{Lamb}\sigma_+\sigma_- + \Sigma_\text{Stark}\sigma_z, \tag{S284}$$

$$\Sigma_\text{Lamb} = -\,\mathrm{Re}\left[\chi_{B_-B_+}[\omega_s]\right] = \sum_j \mathcal{P}\frac{|g_j|^2}{\omega_s - \omega_j}, \tag{S285}$$

$$\Sigma_\text{Stark} = \int_{-\infty}^{\infty}\frac{d\omega}{2\pi}\mathcal{P}\frac{iG^<_{B_-B_+}[\omega]}{\omega_s - \omega} = \sum_j \mathcal{P}\frac{|g_j|^2 n_B(\omega_j)}{\omega_s - \omega_j}, \tag{S286}$$

where $\sigma_+\sigma_- = |e\rangle\langle e|$, σ_Lamb is the Lamb shift, Σ_Stark is the Stark shift, $\chi_{B_-B_+}[\omega]$ is the susceptibility defined in Eq. (S279), $iG^<_{B_-B_+}[\omega]$ is the lesser Green's function that is defined in Eq. (S291), $\mathcal{P}$ represent the Cauchy's principal-value integral, and $n_B(\omega) = [\exp(\beta\omega) - 1]^{-1}$ is the Bose-Einstein distribution. Here, it is worthwhile to write down the susceptibility explicitly

$$\chi_{B_-B_+}[\omega] = \sum_j \frac{|g_j|^2}{\omega_j - \omega - i0^+}. \tag{S287}$$

We note that differently from the Schrieffer-Wolff transformation approach (*38*), such as Ref. (*14*), when there is on-resonant continuum of boson, the Lamb-shift term uses the Cauchy's principal-value integral $\mathcal{P}$. The consideration of the presence of on-resonant boson continuum is important here as we would also like to derive the decay dissipation due to on-resonant bosons, which are the main source of

the decay dissipation. Using the stationary condition of the boson bath and assuming $\langle B_-(t)B_-(0)\rangle = \langle B_+(t)B_+(0)\rangle = 0$ which is valid in our case (or applying the secular approximation), the non-Hermitian term turns into the following form:

$$\mathcal{N}[\rho(t)] = iG^{>}_{B_-B_+}[\omega_s]\,\mathcal{L}[\sigma_-]\,\rho + iG^{<}_{B_-B_+}[\omega_s]\,\mathcal{L}[\sigma_+]\,\rho, \tag{S288}$$

$$\mathcal{L}[\mathcal{O}]\rho \equiv \mathcal{O}\rho\mathcal{O}^\dagger - \frac{1}{2}\left\{\mathcal{O}^\dagger\mathcal{O}, \rho\right\}, \tag{S289}$$

where $\mathcal{L}[\cdots]$ is the Lindblad operator and we define the greater and the lesser Green's functions (*39*):

$$iG^{>}_{BA}(t) = \langle B(t)A(0)\rangle; \quad G^{>}_{BA}[\omega] = \int_{-\infty}^{\infty} dt G^{>}_{BA}(t)e^{i\omega t}, \tag{S290}$$

$$iG^{<}_{BA}(t) = \langle A(0)B(t)\rangle; \quad G^{<}_{BA}[\omega] = \int_{-\infty}^{\infty} dt G^{<}_{BA}(t)e^{i\omega t}. \tag{S291}$$

We note that $iG^{>}_{B_-B_+}[\omega_s]$ and $iG^{<}_{B_-B_+}[\omega_s]$ are real as we have $iG^{<}_{BA}[\omega], iG^{>}_{BA}[\omega] \in \mathbb{R}$ for $B = A^\dagger$. More explicitly, they are written as

$$iG^{>}_{B_-B_+}[\omega_s] = (n_B(\omega_s) + 1)\sum_j |g_j|^2\, 2\pi\delta(\omega_s - \omega_j), \tag{S292}$$

$$iG^{<}_{B_-B_+}[\omega_s] = n_B(\omega_s)\sum_j |g_j|^2\, 2\pi\delta(\omega_s - \omega_j). \tag{S293}$$

The T_1 decay rate [i.e. the decay rate of the population in the form $\langle\sigma_z(t)\rangle/\langle\sigma_z(0)\rangle = \exp(-t/T_1)$] reads

$$1/T_1 = iG^{>}_{B_-B_+}[\omega_s] + iG^{<}_{B_-B_+}[\omega_s] = iG^{K}_{B_-B_+}[\omega_s], \tag{S294}$$

where we define the Keldysh Green's function (*39*):

$$iG^{K}_{BA}(t) = iG^{>}_{BA}(t) + iG^{<}_{BA}(t) = \langle\{B(t), A(0)\}\rangle, \tag{S295}$$

$$G^{K}_{BA}[\omega] = \int_{-\infty}^{\infty} dt G^{K}_{BA}(t)e^{i\omega t}. \tag{S296}$$

More explicitly, using Eqs. (S292) and (S293), we obtain

$$\begin{aligned} 1/T_1 &= (2n_B(\omega_s) + 1)\sum_j |g_j|^2\, 2\pi\delta(\omega_s - \omega_j), \\ &= \coth(\beta\omega_s/2)\sum_j |g_j|^2\, 2\pi\delta(\omega_s - \omega_j), \end{aligned} \tag{S297}$$

which is used in Eq. (S170) by replacing the sum into integral.

SIII.6.1 Relationship between longitudinal decay rate and the Lamb shift

Importantly, the T_1 decay rate and the Lamb shift (self-energy shift) at thermal equilibrium are related by the fluctuation-dissipation relation and the Kramers-Kronig relation. From the fluctuation-dissipation relation (Sec. SIII.6.2), at thermal equilibrium of the boson magnon bath, we obtain

$$iG^K_{BA}[\omega] = A_{BA}[\omega]\coth[\beta\omega/2], \tag{S298}$$

where $A_{BA}[\omega]$ is the spectral function defined by (*39*):

$$A_{BA}(t) = iG^>_{BA}(t) - iG^<_{BA}(t) = iG^R_{BA}(t) - iG^A_{BA}(t) = \langle[B(t), A(0)]\rangle, \tag{S299}$$

$$A_{BA}[\omega] = \int_{-\infty}^{\infty} dt A_{BA}(t)e^{i\omega t}. \tag{S300}$$

Using the relation Eq. (S278), the spectral function is shown to be proportional to the imaginary part of the retarded Green's function when $A = B^\dagger$:

$$A_{BA}[\omega] = -2\,\mathrm{Im}\left[G^R_{BA}[\omega]\right],\ \text{for } B = A^\dagger. \tag{S301}$$

Combining Eqs. (S301), (S298), (S294), and (S279), we obtain the relation between the T_1 decay rate and the imaginary part of the susceptibility $\chi_{B_-B_+}[\omega_s]$:

$$1/T_1 = iG^K_{B_-B_+}[\omega_s] = A_{B_-B_+}[\omega_S]\coth[\beta\omega_s/2] = 2\,\mathrm{Im}\left[\chi_{B_-B_+}[\omega_s]\right]\coth[\beta\omega_s/2]. \tag{S302}$$

This should be seen in contrast to the the Lamb shift (self-energy shift) term derived in Eq. (S285), which is the real part of the susceptibility. The real and imaginary part of the susceptibility are in turn related by the Kramers-Kronig relation (Sec. SIII.6.3):

$$\mathrm{Re}\left[\chi_{B_-B_+}[\omega]\right] = -\mathcal{P}\int_{-\infty}^{\infty}\frac{d\omega'}{\pi}\frac{\mathrm{Im}\left[\chi_{B_-B_+}[\omega']\right]}{\omega-\omega'}, \tag{S303}$$

$$\mathrm{Im}\left[\chi_{B_-B_+}[\omega]\right] = +\mathcal{P}\int_{-\infty}^{\infty}\frac{d\omega'}{\pi}\frac{\mathrm{Re}\left[\chi_{B_-B_+}[\omega']\right]}{\omega-\omega'}. \tag{S304}$$

Therefore, if we have access to the frequency dependence of the longitudinal decay rate T_1, i.e. $T_1 = T_1[\omega]$, we obtain using Eqs. (S285), (S303), and (S302):

$$\mathrm{Re}\left[\chi_{B_-B_+}[\omega_s]\right] = -\Sigma_{\mathrm{Lamb}} = -\mathcal{P}\int_{-\infty}^{\infty}\frac{d\omega'}{\pi}\frac{1}{\omega_s-\omega'}\cdot\frac{1/T_1[\omega']}{2\coth[\beta\omega'/2]}. \tag{S305}$$

This equation clarifies the relation between the Lamb shift (self-energy shift) (Σ_{Lamb}) and the decay dissipation (T_1).

SIII.6.2 Fluctuation-dissipation relation

In this section, we show the fluctuation-dissipation relation. Although it is a well-known relation (*39*), as we are considering the case with Green's functions $G^{>}_{BA}$ and $G^{<}_{BA}$ containing operators A and B that are not Hermitian, it is instructive to show and verify the relation here. We assume $[\mathcal{H}, \mu N] = 0$ [Note: we drop the subscript of $\mathcal{H}_B$ and write it as $\mathcal{H}$ because we only consider the boson Hilbert space] and $Ae^{\beta\mu N} = e^{\beta\mu(N-1)}A$ (satisfied when $[N, A] = A$), where N is the number operator (in our case, $N = \sum_j b_j^\dagger b_j$), μ is the chemical potential (note that μ can be zero and in that case $[\mathcal{H}, \mu N] = 0$ and $Ae^{\beta\mu N} = e^{\beta\mu(N-1)}A$ always hold), and A is the operator that appears in the Green's functions $G^{>}_{BA}$ and $G^{<}_{BA}$. Then, in the time domain, the greater and the lesser Green's function are related by:

$$\begin{aligned} iG^{>}_{BA}(t) &= Z^{-1}\,\mathrm{Tr}\left[e^{-\beta(\mathcal{H}-\mu N)}B(t)A\right] = Z^{-1}\,\mathrm{Tr}\left[e^{-\beta(\mathcal{H}-\mu N)}e^{+i\mathcal{H}t}Be^{-i\mathcal{H}t}A\right], \\ &= Z^{-1}\,\mathrm{Tr}\left[e^{\beta\mu N}e^{+i\mathcal{H}(t+i\beta)}Be^{-i\mathcal{H}(t+i\beta)}e^{-\beta\mathcal{H}}A\right] = Z^{-1}\,\mathrm{Tr}\left[e^{\beta\mu N}B(t+i\beta)e^{-\beta\mathcal{H}}A\right], \\ &= Z^{-1}\,\mathrm{Tr}\left[e^{-\beta\mathcal{H}}Ae^{\beta\mu N}B(t+i\beta)\right] = Z^{-1}\,\mathrm{Tr}\left[e^{-\beta\mathcal{H}}e^{\beta\mu(N-1)}AB(t+i\beta)\right], \\ &= e^{-\beta\mu}Z^{-1}\,\mathrm{Tr}\left[e^{-\beta(\mathcal{H}-\mu N)}AB(t+i\beta)\right] = e^{-\beta\mu}iG^{<}_{BA}(t+i\beta), \end{aligned} \tag{S306}$$

where $Z = \mathrm{Tr}[e^{\beta(\mathcal{H}-\mu N)}]$ is the partition function. In the frequency domain, this becomes

$$\begin{aligned} G^{>}_{BA}[\omega] &= e^{-\beta\mu}\int_{-\infty}^{\infty} dte^{i\omega t}G^{<}_{BA}(t+i\beta) = e^{-\beta\mu}\int_{-\infty+i\beta}^{\infty+i\beta} dt'e^{i\omega(t'-i\beta)}G^{<}_{BA}(t'), \\ &= e^{\beta(\omega-\mu)}\int_{-\infty+i\beta}^{\infty+i\beta} dt'e^{i\omega t'}G^{<}_{BA}(t') = e^{\beta(\omega-\mu)}\int_{-\infty}^{\infty} dt'e^{i\omega t'}G^{<}_{BA}(t'), \\ &= e^{\beta(\omega-\mu)}G^{<}_{BA}[\omega]. \end{aligned} \tag{S307}$$

This is called the detailed balance or the detailed balancing condition (*39*). Using this condition, we obtain the fluctuation-dissipation relation:

$$\begin{aligned} iG^{K}_{BA}[\omega] &= i\left(G^{>}_{BA}[\omega] + G^{<}_{BA}[\omega]\right) = \frac{i\left(G^{>}_{BA}[\omega] + G^{<}_{BA}[\omega]\right)}{i\left(G^{>}_{BA}[\omega] - G^{<}_{BA}[\omega]\right)}A_{BA}[\omega], \\ &= A_{BA}[\omega]\coth[\beta(\omega-\mu)/2]. \end{aligned} \tag{S308}$$

When we use this relation in Eq. (S298), we assume that the chemical potential is zero $\mu = 0$. While there are interest in the case with non-zero magnon chemical potential $\mu \neq 0$ obtained by gently exciting the magnons via a MSL drive and through the injection of spin currents (*40*), here we do not actively drive the magnons and hence $\mu = 0$ is justified.

SIII.6.3 Kramers-Kronig relation

In this section, we show the Kramers-Kronig relation. Although it is a well-known relation, as we are considering the case where operators A and B appearing in the susceptibility χ_{BA} are not Hermitian, it is instructive to show and verify the relation here. From the definition of $\chi_{BA}(t)$, we can write

$$\chi_{BA}(t) = i\Theta(t)A_{BA}(t), \tag{S309}$$

which is a manifestation of the causality. Its Fourier transform is given by, with the use of the convolution theorem,

$$\chi_{BA}[\omega] = i\int_{-\infty}^{\infty}\frac{d\omega'}{2\pi}\Theta\left[\omega-\omega'\right]A_{BA}\left[\omega'\right] = -\int_{-\infty}^{\infty}\frac{d\omega'}{2\pi}\frac{A_{BA}\left[\omega'\right]}{\omega-\omega'+i0^{+}}, \tag{S310}$$

where $0^{+}\to+0$ and $\Theta[\cdots]$ is the Fourier transform of the Heaviside step function given by

$$\Theta[\omega] = \frac{i}{\omega+i0^{+}}. \tag{S311}$$

Using the Plemelj formula $1/(\omega+i0^{+}) = \mathcal{P}/\omega - i\pi\delta(\omega)$, we obtain

$$\chi_{BA}[\omega] = \chi'_{BA}[\omega] + i\chi''_{BA}[\omega], \tag{S312}$$

where we define the first part ($\chi'_{BA}[\omega]$) and the second part ($\chi''_{BA}[\omega]$) as

$$\chi'_{BA}[\omega] \equiv -\frac{G^{R}_{BA}[\omega]+G^{A}_{BA}[\omega]}{2} = -\mathcal{P}\int_{-\infty}^{\infty}\frac{d\omega'}{2\pi}\frac{A_{BA}\left[\omega'\right]}{\omega-\omega'}, \tag{S313}$$

$$\chi''_{BA}[\omega] \equiv -\frac{G^{R}_{BA}[\omega]-G^{A}_{BA}[\omega]}{2i} = \frac{A_{BA}[\omega]}{2}. \tag{S314}$$

We note that the first and the second part are not necessarily the real and the imaginary part, respectively, although we show that they become the real and the imaginary part under a certain condition. For these susceptibilities, we have the following Kramers-Kronig relation:

$$\chi'_{BA}[\omega] = -\mathcal{P}\int_{-\infty}^{\infty}\frac{d\omega'}{\pi}\frac{\chi''_{BA}\left[\omega'\right]}{\omega-\omega'}, \tag{S315}$$

$$\chi''_{BA}[\omega] = +\mathcal{P}\int_{-\infty}^{\infty}\frac{d\omega'}{\pi}\frac{\chi'_{BA}\left[\omega'\right]}{\omega-\omega'}. \tag{S316}$$

Furthermore, the first and the second part become the real and the imaginary part when $B = A^{\dagger}$, i.e.

$$\left.\begin{array}{l}\chi'_{BA}[\omega] = \mathrm{Re}\left[\chi_{BA}[\omega]\right]\\ \chi''_{BA}[\omega] = \mathrm{Im}\left[\chi_{BA}[\omega]\right]\end{array}\right\}\mathrm{For} B = A^{\dagger}. \tag{S317}$$

In Eqs. (S303) and (S304), we use the fact that $B = B_- = (B_+)^\dagger = A^\dagger$ is satisfied in the Green's functions, and hence the Kramers-Kronig relation simply connects the real part and the imaginary part of the susceptibility $\chi_{B_-B_+}[\omega]$.

SIII.6.4 Identification of the susceptibility and the self-energy

In the main text, we call the susceptibility χ as the self-energy. We argued that the qubit frequency ω_s is modified to $\omega_\mathrm{s} \to \omega_\mathrm{s} - \chi$ in the presence of the qubit-magnon coupling, where $\chi = \chi_{B_-B_+}[\omega_\mathrm{s}]$ is the self-energy (more accurately, the susceptibility) of the qubit. We further discussed that the time evolution of the excited-state qubit wave function [i.e. $\psi_e(t) = \langle e|\psi(t)\rangle$] is modified to

$$\psi_e(t > 0) = e^{-i\omega_\mathrm{s} t}\psi_e(0) \to e^{-i(\omega_\mathrm{s}-\chi)t}\psi_e(0) = e^{-\chi'' t}e^{-i(\omega_\mathrm{s}-\chi')t}\psi_e(0), \tag{S318}$$

indicating that χ' and χ'' are responsible for the energy-shift and the decay properties of the qubit, respectively. In the following paragraphs, we validate this argument by (1) considering the Lindblad master equation in the low-temperature limit $\beta \to \infty$ with the non-Hermitian Hamiltonian formalism, and (2) using the Dyson equation at absolute zero temperature.

We firstly consider the low-temperature limit of the Lindblad master equation. In this limit, the Lindblad master equation Eq. (S270) becomes

$$\frac{d}{dt}\rho(t) = -i[\mathcal{H}_0 - \chi'\sigma_+\sigma_-, \rho] + 2\chi''\mathcal{L}[\sigma_-]\rho, \tag{S319}$$

where we used the fact that at low temperature, the effective Hamiltonian $\mathcal{H}_\mathrm{eff}$ in Eq. (S284) becomes $\mathcal{H}_\mathrm{eff} \to -\chi'\sigma_+\sigma_-$, and the greater and the lesser Green's functions in the non-Hermitian term $\mathcal{N}[\rho(t)]$ in Eq. (S288) become $iG^>_{B_-B_+}[\omega_\mathrm{s}] \to 2\chi''$ and $iG^<_{B_-B_+}[\omega_\mathrm{s}] \to 0$, respectively. For the convenience of the argument, we redefine the bare qubit Hamiltonian by shifting it by a constant amount $\omega_\mathrm{s}/2$, i.e.,

$$\mathcal{H}_0 = \frac{\omega_\mathrm{s}}{2}\sigma_z + \omega_\mathrm{s}/2 = \omega_\mathrm{s}\sigma_+\sigma_-, \tag{S320}$$

such that the ground state energy is zero, not $-\omega_\mathrm{s}/2$. Writing the Lindblad operator $\mathcal{L}[\cdots]$ explicitly using the definition Eq. (S289), Eq. (S319) becomes

$$\begin{aligned} \frac{d}{dt}\rho(t) &= -i[\mathcal{H}_0 - \chi'\sigma_+\sigma_-, \rho] + 2\chi''\left(\sigma_-\rho\sigma_+ - \frac{1}{2}\{\sigma_+\sigma_-, \rho\}\right), \\ &= -i(\mathcal{H}_\mathrm{nh}\rho - \rho\mathcal{H}_\mathrm{nh}^\dagger) + 2\chi''\sigma_-\rho\sigma_+, \end{aligned} \tag{S321}$$

where we defined the non-Hermitian Hamiltonian $\mathcal{H}_{\rm nh}$ as

$$\mathcal{H}_{\rm nh} = \mathcal{H}_0 - (\chi' + i\chi'')\sigma_+\sigma_- = \mathcal{H}_0 - \chi\sigma_+\sigma_- = (\omega_{\rm s} - \chi)\,\sigma_+\sigma_-. \tag{S322}$$

The non-Hermitian Hamiltonian dynamics is obtained by dropping the last term in Eq. (S321) (dropping the quantum jumps (*41*)), leading to

$$\frac{d}{dt}\rho(t) = -i(\mathcal{H}_{\rm nh}\rho - \rho\mathcal{H}_{\rm nh}^\dagger). \tag{S323}$$

This indicates that the Schrödinger equation under the non-Hermitian Hamiltonian is given by $i\partial_t|\psi\rangle = \mathcal{H}_{\rm nh}|\psi\rangle$ and $-i\partial_t\langle\psi| = \langle\psi|\mathcal{H}_{\rm nh}^\dagger$. It is clear from Eq. (S323) that the qubit frequency is modified to $\omega_{\rm s} \to \omega_{\rm s} - \chi$. Furthermore, by expanding the wave function as $|\psi(t)\rangle = \psi_g(t)|g\rangle + \psi_e(t)|e\rangle$, the time evolution of the excited-state wave function becomes $i\psi_e(t) = (\omega_{\rm s} - \chi)\psi_e(t)$, resulting in $\psi_e(t) = e^{-i(\omega_{\rm s}-\chi)t}\psi_e(0)$ as shown in Eq. (S318). Note that if we follow the same procedure at a finite temperature, we obtain

$$\mathcal{H}_{\rm nh} = \mathcal{H}_0 + \left(\int_{-\infty}^{\infty}\frac{d\omega}{2\pi}\frac{iG^{>}_{B_-B_+}[\omega]}{\omega_s - \omega + i0^+}\right)\sigma_+\sigma_- - \left(\int_{-\infty}^{\infty}\frac{d\omega}{2\pi}\frac{iG^{<}_{B_-B_+}[\omega]}{\omega_s - \omega - i0^+}\right)\sigma_-\sigma_+, \tag{S324}$$

which is more directly obtained from other derivation procedures of the Lindblad master equation such as Refs. (*36*) (chapter 3) and (*42*). We can recover Eq. (S322) by using $iG^{>}_{BA} = iG^{<}_{BA} + A_{BA}$ [Eq. (S299)], $\chi_{BA}[\omega] = -\int_{-\infty}^{\infty}(d\omega'/2\pi)A_{BA}[\omega']/(\omega - \omega' + i0^+)$ [Eq. (S310)], and $iG^{>}_{B_-B_+}[\omega_{\rm s}] \to 0$ at low temperature $\beta \to \infty$.

We now use the Dyson equation at zero temperature to obtain the qubit-frequency modification $\omega_{\rm s} \to \omega_{\rm s} - \chi$. We again redefine the bare Hamiltonian $\mathcal{H}_0$ via Eq. (S320) such that the qubit ground-state energy is zero. Then, we define the excited-state propagator $\mathcal{F}$ via

$$\begin{aligned}\mathcal{F}(t) \equiv \langle e|U(t)|e\rangle\Theta(t) &= \langle{\rm vac}|c_e U(t) c_e^\dagger|{\rm vac}\rangle\Theta(t) = \langle{\rm vac}|U(t)c_{e,{\rm H}}(t)c_{e,{\rm H}}^\dagger(0)|{\rm vac}\rangle\Theta(t), \\ &= \langle{\rm vac}|c_{e,{\rm H}}(t)c_{e,{\rm H}}^\dagger(0)|{\rm vac}\rangle\Theta(t) = \langle{\rm vac}|\mathcal{T}c_{e,{\rm H}}(t)c_{e,{\rm H}}^\dagger(0)|{\rm vac}\rangle, \end{aligned} \tag{S325}$$

where $U(t) \equiv \exp(-i\mathcal{H}t)$ is the time-evolution operator for the total Hamiltonian $\mathcal{H}$ given in Eq. (S280), $c_\alpha^\dagger$ is the qubit creation operator for the state $|\alpha\rangle = c_\alpha^\dagger|{\rm vac}\rangle$ with the vacuum state $|{\rm vac}\rangle$, and the operators with a subscript H indicate the Heisenberg representation $\mathcal{O}_{\rm H} = U^\dagger(t)\mathcal{O}U(t)$. We note that

$c_\alpha^\dagger$ can be a fermion or a boson operator. As the particle number $c_g^\dagger c_g + c_e^\dagger c_e$ is conserved and we only focus on its eigenspace with eigenvalue zero or one, whether $c_\alpha^\dagger$ is a fermion or a boson operator does not matter. In the second line above we used the conjugate of $U^\dagger(t)|\mathrm{vac}\rangle = |\mathrm{vac}\rangle$ because $|\mathrm{vac}\rangle$ is the zero-energy eigenstate of both $\mathcal{H}$ and $\mathcal{H}_0$ (recall $\sigma_- = c_g^\dagger c_e$ in our notation), and in the last equality we introduced the time-ordering operator $\mathcal{T}$ by noticing $c_\alpha(t)|\mathrm{vac}\rangle = U^\dagger(t)c_\alpha U(t)|\mathrm{vac}\rangle = U^\dagger(t)c_\alpha|\mathrm{vac}\rangle = 0$. Using the linked-cluster theorem (*10*) in the last expression of Eq. (S325) and recalling that the vacuum state $|\mathrm{vac}\rangle$ is the zero-energy eigenstate of both $\mathcal{H}$ and $\mathcal{H}_0$, we obtain the Dyson equation

$$\mathcal{F}[\omega] = \mathcal{F}_0[\omega] + \mathcal{F}_0[\omega]\Sigma[\omega]\mathcal{F}[\omega], \tag{S326}$$

where $\mathcal{F}_0[\omega] = 1/(\omega - \omega_\mathrm{s} + i0^+)$ is the Fourier-transform of the bare qubit propagator $\mathcal{F}_0(t) \equiv \langle\mathrm{vac}|\mathcal{T}c_{e,\mathrm{I}}(t)c_{e,\mathrm{I}}^\dagger(0)|\mathrm{vac}\rangle$, with subscript I indicating the interaction picture $\mathcal{O}_\mathrm{I} = e^{i(\mathcal{H}_0+\mathcal{H}_B)t}\mathcal{O}e^{-i(\mathcal{H}_0+\mathcal{H}_B)t}$, and self-energy

$$\Sigma[\omega] = i\int_{-\infty}^{\infty}\frac{d\omega_1}{2\pi}\sum_j |g_j|^2\mathcal{D}_{0,j}[\omega-\omega_1]\mathcal{G}_0[\omega_1]. \tag{S327}$$

Here, the bare propagators of the boson $\mathcal{D}_{0,j}[\omega] = 1/(\omega - \omega_j + i0^+)$ and the qubit ground state $\mathcal{G}_0[\omega] = 1/(\omega + i0^+)$ are the Fourier transforms of $\mathcal{D}_{0,j}(t) \equiv \langle\mathrm{vac}|\mathcal{T}b_j(t)b_j^\dagger(0)|\mathrm{vac}\rangle$ and $\mathcal{G}_0(t) \equiv \langle\mathrm{vac}|\mathcal{T}c_g(t)c_g^\dagger(0)|\mathrm{vac}\rangle$, respectively. Direct computation results in

$$\Sigma[\omega] = \sum_j \frac{|g_j|^2}{\omega - \omega_j + i0^+}. \tag{S328}$$

Comparing Eqs. (S287) and (S328), we confirm that the susceptibility derived in Sec. SIII.6 is indeed the same as the self-energy, which is broadly studied in the quantum field theory (despite a minor difference in the sign convention), i.e.,

$$\chi_{B_-B_+}[\omega] = -\Sigma[\omega]. \tag{S329}$$

When the coupling strength $|g_j|$ is sufficiently small, the pole of the excited-state dressed propagator can be regarded as being near the original pole $\omega = \omega_\mathrm{s}$, allowing us to approximate

$$\mathcal{F}[\omega] = \frac{1}{\omega - (\omega_\mathrm{s} - \chi_{B_-B_+}[\omega]) + i0^+} \approx \frac{1}{\omega - (\omega_\mathrm{s} - \chi_{B_-B_+}[\omega_\mathrm{s}]) + i0^+} = \frac{1}{\omega - (\omega_\mathrm{s} - \chi) + i0^+}, \tag{S330}$$

where we approximated $\chi_{B_-B_+}[\omega] \approx \chi_{B_-B_+}[\omega_\mathrm{s}] = \chi$ in the denominator. This confirms that the qubit frequency is modified to $\omega_\mathrm{s} \to \omega_\mathrm{s} - \chi$. Furthermore, noticing that the ground state $|g\rangle$ (with no boson occupation) satisfies $\mathcal{H}|g\rangle = 0$ and hence $U(t)|g\rangle = |g\rangle$, we confirm Eq. (S318) via $\psi_e(t) = \langle e|\psi(t)\rangle = \langle e|U(t)|\psi(0)\rangle = \langle e|U(t)(\psi_g(0)|g\rangle + \psi_e(0)|e\rangle) = \langle e|U(t)|e\rangle\psi_e(0) = \mathcal{F}(t)\psi_e(0) \approx e^{-i(\omega-\chi)t}\psi_e(0)$ for $t > 0$.

SIV Foundation of the analysis using fluctuation-dissipation and Kramers-Kronig relations to obtain self-energy shift

We showed in Eq. (S305) that the real part of the susceptibility (self-energy shift or the Lamb shift) can be obtained by the combination of the fluctuation-dissipation relation (FDR) and the Kramers-Kronig relation (KKR) using the longitudinal (T_1) relaxation time as a function of the qubit frequency ω, i.e. $T_1 = T_1[\omega]$. However, it is unclear how to probe experimentaly the frequency dependence of $T_1[\omega]$. In practice, probing the frequency ω dependence of T_1 for NV centers is performed by changing the external magnetic field H controlling the NV-center transition frequency. In such condition, we obtain the magnetic field dependence of the longitudinal relaxation time $T_1 = T_1[\omega_{\rm NV}(H)] \equiv T_1(H)$, where $\omega_{\rm NV}(H)$ is the magnetic field dependence of the NV-center transition frequency. For example, when the external magnetic field is along the NV-center's main symmetry axis, $\omega_{\rm NV}(H) = D_{\rm NV} - \gamma\mu_0 H$. We note here that the square bracket $[\cdots]$ for the function argument is used for the frequency ω argument consistently in this article. Now, probing frequency dependence of $T_1[\omega]$ may appear straightforward. In the magnon-mediated susceptibility case, however, changing the external magnetic field will also change the magnon dispersion relation and the NV-magnon coupling. This makes the magnon-mediated susceptibility explicitly dependent on the external magnetic field, adding an extra complexity to the problem. Fortunately, the problem can be simplified because the external magnetic field only changes substantially the frequency detuning between the NV-center frequency and the magnon-mode frequency due to the Zeeman energy.

For the case of the NV center interacting with magnons, using the FDR Eq. (S302), we obtain the relationship between the longitudinal relaxation time $T_1(H)$ and the magnon-induced susceptibility $\chi_{B_-B_+(\omega_H)}[\omega]$ as

$$\chi''_{B_-B_+(\omega_H)}[\omega_{\rm NV}(H)] = \frac{1/T_1(H)}{2\coth(\beta\omega_{\rm NV}(H)/2)}, \tag{S331}$$

where the subscript (ω_H) in the magnon-mediated susceptibility $\chi_{B_-B_+(\omega_H)}[\omega]$ indicates that the susceptibility explicitly depends on the external magnetic field parametrized by $\omega_H = \gamma\mu_0 H$, as we discussed in the previous paragraph. This field dependence can be due to the change in both the dis-

persion relation of magnons as well as their coupling to the NV center [see Eq. (S287)]. Here, the dispersion relation for magnons depends on the external magnetic field due to the Zeeman interaction [see Eq. (S165)]. In contrast, the NV-magnon coupling strength depends on the external magnetic field via the following two factors. Firstly, it can change due to the change in the magnon-originated magnetic field generated at the NV position, as the external magnetic field can modify the magnon-mode profile and the resulting fringe magnetic field. Secondly, when the external magnetic field is not parallel to the NV-center's main symmetry axis, its ground state $|g\rangle$ and the first excited state $|e\rangle$ also change as a function of the external magnetic field, which will result in the change of the NV-magnon coupling strength [as the NV operators σ_+ and σ_- in the interaction Hamiltonian Eq. (S169) are re-defined]. Now, as a magnon-induced susceptibility probed by the NV center, we define the following susceptibility without the subscript $B_-B_+(\omega_H)$ and with the magnetic field argument

$$\chi(H) \equiv \chi_{B_-B_+(\omega_H)}[\omega_{\rm NV}(H)]. \tag{S332}$$

We obtain the imaginary part $\chi''(H)$ from the $T_1(H)$ measurement via Eq. (S331). Our goal in this section is to obtain the real part of the susceptibility $\chi'(H)$ using the KKR. This is, however, nontrivial because $\chi_{B_-B_+(\omega_H)}[\omega_{\rm NV}(H)]$ on the right-hand side of Eq. (S332) depends on the external magnetic field H both via the frequency argument $[\omega_{\rm NV}(H)]$ and the subscript (ω_H). However, to use the KKR [see Eq. (S315)], i.e.

$$\chi'_{B_-B_+(\omega_H)}[\omega] = -\mathcal{P}\int_{-\infty}^{\infty} \frac{d\omega'}{\pi} \frac{\chi''_{B_-B_+(\omega_H)}[\omega']}{\omega - \omega'}, \tag{S333}$$

we need to vary the frequency argument of $\chi''_{B_-B_+(\omega_H)}[\omega]$ without changing the subscript (ω_H).

We need some assumptions to eliminate the field dependence in the subscript (ω_H) on the right-hand side of Eq. (S332) to apply the KKR for $\chi(H)$. To this end, we assume that the effect of the magnetic-field dependence on the magnon-induced susceptibility $\chi_{B_-B_+(\omega_H)}[\omega]$ is dominantly caused by the parallel shift of the magnon dispersion relation in the frequency direction by the Zeeman interaction [see Eq. (S165)]. Recalling that the susceptibility $\chi_{B_-B_+}[\omega]$ in Eq. (S287) is invariant under the simultaneous changes $\omega_j \to \omega_j - \omega_H$ and $\omega \to \omega - \omega_H$, this assumption leads to

$$\chi_{B_-B_+(\omega_H)}[\omega] \approx \chi_{B_-B_+(0)}[\omega - \omega_H]. \tag{S334}$$

Detailed discussions on Eq. (S334) can be found in Sec. SIV.1. Here, we note that the choice of the base field [i.e. $(\omega_H) \to (0)$ in the subscript of the susceptibility] does not need to be zero, and we chose it to be the zero field only for the simplicity of the argument. In fact, we could have approximated $\chi_{B_-B_+(\omega_{H_c}+\Delta\omega_H)}[\omega] \approx \chi_{B_-B_+(\omega_{H_c})}[\omega - \Delta\omega_H]$ for any base field H_c, and field deviation $\Delta\omega_H = \omega_H - \omega_{H_c}$. Therefore, the subtlety regarding the behavior near the zero field, such as the magnetic coercivity, does not matter here.

Now, applying Eq. (S334) into Eq. (S332), we obtain

$$\chi(H) \approx \chi_{B_-B_+(0)}[\Omega(H)], \tag{S335}$$

where we defined the NV-magnon-detuning function $\Omega(H) \equiv \omega_{\rm NV}(H) - \omega_H$. We call it as the NV-magnon detuning because it parametrizes the NV frequency relative to the magnon spectrum's lower bound ω_H. Then, we apply the KKR Eq. (S333) for the $\omega_H = 0$ case (in the subscript) to the real part of the right-hand side of Eq. (S335), and obtain

$$\begin{aligned} \chi'_{B_-B_+(0)}[\Omega(H)] &= -\mathcal{P}\int_{-\infty}^{\infty} \frac{d\Omega'}{\pi} \frac{\chi''_{B_-B_+(0)}[\Omega']}{\Omega(H) - \Omega'}, \\ &= -\mathcal{P}\int_{-\infty}^{\infty} \frac{dH'}{\pi} \left|\frac{d\Omega(H')}{dH'}\right| \frac{\chi''_{B_-B_+(0)}[\Omega(H')]}{\Omega(H) - \Omega(H')}, \end{aligned} \tag{S336}$$

where in the second line we changed the integration variable from $\Omega' = \Omega(H')$ into H'. To perform the change of variable, the function $\Omega(H)$ is extended to the $H < 0$ range monotonically such that $\Omega(H \to -\infty) = \infty$ [for example, when the NV-center's main symmetry axis is parallel to the external magnetic field, we use $\Omega(H) = D_{\rm NV} - 2\gamma\mu_0 H$], and we assume that $\Omega(H > 0)$ is monotonically decreasing with $\Omega(H \to \infty) = -\infty$ (which is true when the NV-center's main symmetry axis is parallel to the external magnetic field) to obtain the integration range in Eq. (S336).

Substituting the real and the imaginary part of Eq. (S335) into both-hand sides of Eq. (S336), we obtain the KKR for the field-dependent susceptibility $\chi(H)$ as

$$\chi'(H) \approx -\mathcal{P}\int_{-\infty}^{\infty} \frac{dH'}{\pi} \left|\frac{d\Omega(H')}{dH'}\right| \frac{\chi''(H)}{\Omega(H) - \Omega(H')}, \tag{S337}$$

where the imaginary part $\chi''(H)$ on the right-hand side can be obtained from the $T_1(H)$ measurement using the FDR Eq. (S331) with the definition of $\chi(H)$ in Eq. (S332). To simplify the expression

further, we consider the special simple case where the NV-center's main symmetry axis is parallel to the external magnetic field [note that in this case the assumption/approximation Eq. (S334) becomes more justifiable as discussed above]. Then, using the explicit form $\Omega(H) = D_{\rm NV} - 2\gamma\mu_0 H$, we obtain

$$\chi'(H) \approx \mathcal{P}\int_{-\infty}^{\infty} \frac{dH'}{\pi}\frac{\chi''(H')}{H-H'}, \tag{S338}$$

$$\chi''(H) = \frac{1/T_1(H)}{2\coth(\beta\omega_{\rm NV}(H)/2)}, \tag{S339}$$

where for completeness we show the FDR for $\chi''(H)$ in Eq. (S339) by combining Eqs. (S331) and (S332).

To compare the the self-energy shift χ' obtained experimentally [by Eqs. (S338) and (S339)] and the calculated effective NV-NV coupling $g_{\rm eff}$ obtained from the derivation of the Markovian Lindblad master equation, we numerically evaluated (see Sec. SIV.2)

$$g_{\rm eff} = \sum_{n=0}^{N}\int \frac{d\mathbf{k}}{(2\pi)^2}\mathcal{P}\frac{|g_{\mathbf{k},n}|^2\exp[i\mathbf{k}\cdot(\mathbf{r}_{{\rm NV}_1} - \mathbf{r}_{{\rm NV}_2})]}{\omega_{\mathbf{k},n} - \omega_{\rm NV}}, \tag{S340}$$

where we replaced $\mathcal{P}(\omega_{\mathbf{k},n} - \omega_{\rm NV})^{-1} \to (\omega_{\mathbf{k},n} - \omega_{\rm NV})/((\omega_{\mathbf{k},n} - \omega_{\rm NV})^2 + \delta^2)$ with small δ to avoid the singularity in the numerical evaluation.

SIV.1 Assumption on the magnon-induced susceptibility

To obtain Eq. (S334), we assumed that the effect of the magnetic-field dependence on the magnon-induced susceptibility $\chi_{B_-B_+(\omega_H)}[\omega]$ is dominantly caused by a parallel shift of the magnon dispersion relation. While in principle, the magnetic field dependence would also change the NV-magnon coupling strength, this effect is small compared to the effect arrising from the dispersion shift. More explicitly, this assumption indicates that the magnon modes that dominantly contribute to the susceptibility with large enough NV-magnon couplings follow

$$\omega_{\mathbf{k},n}^{(\omega_H)} \approx \omega_{\mathbf{k},n}^{(0)} + \omega_H; \quad g_{\mathbf{k},n}^{(\omega_H)} \approx g_{\mathbf{k},n}^{(0)}, \tag{S341}$$

where $\omega_{\mathbf{k},n}^{(\omega_H)}$ is the magnon-mode frequency and $g_{\mathbf{k},n}^{(\omega_H)}$ is the NV-magnon coupling, with superscript (ω_H) showing the explicit magnetic-field dependencies. Now, we rewrite the susceptibility $\chi_{B_-B_+(\omega_H)}$

in terms of the parameters used in Eq. (S287), as

$$\chi_{B_-B_+(\omega_H)}[\omega] \equiv \chi_{B_-B_+}^{\left\{\omega_{\mathbf{k},n}^{(\omega_H)};g_{\mathbf{k},n}^{(\omega_H)}\right\}}[\omega], \quad \text{(S342)}$$

$$\chi_{B_-B_+}^{\left\{\omega_{\mathbf{k},n};g_{\mathbf{k},n}\right\}}[\omega] \equiv \sum_n \int \frac{d\mathbf{k}}{(2\pi)^2} \frac{|g_{\mathbf{k},n}|^2}{\omega_{\mathbf{k},n} - \omega - i0^+}. \quad \text{(S343)}$$

Then, using Eq. (S341), we obtain

$$\begin{aligned} \chi_{B_-B_+(\omega_H)}[\omega] = \chi_{B_-B_+}^{\left\{\omega_{\mathbf{k},n}^{(\omega_H)};g_{\mathbf{k},n}^{(\omega_H)}\right\}}[\omega] &\approx \chi_{B_-B_+}^{\left\{\omega_{\mathbf{k},n}^{(0)}+\omega_H;g_{\mathbf{k},n}^{(0)}\right\}}[\omega], \\ &= \chi_{B_-B_+}^{\left\{\omega_{\mathbf{k},n}^{(0)};g_{\mathbf{k},n}^{(0)}\right\}}[\omega-\omega_H] = \chi_{B_-B_+(0)}[\omega-\omega_H], \quad \text{(S344)} \end{aligned}$$

where in the second line we used the fact that the susceptibility $\chi_{B_-B_+}^{\left\{\omega_{\mathbf{k},n};g_{\mathbf{k},n}\right\}}[\omega]$ in Eq. (S343) is invariant under the simultaneous changes $\omega_{\mathbf{k},n} \to \omega_{\mathbf{k},n} - \omega_H$ and $\omega \to \omega - \omega_H$. We note that the base-magnetic-field independent expression of the first part of Eq. (S341) is

$$\omega_{\mathbf{k},n}^{(\omega_H+\omega_{\Delta H})} \approx \omega_{\mathbf{k},n}^{(\omega_H)} + \omega_{\Delta H} \quad \to \quad 1 \approx \partial\omega_{\mathbf{k},n}^{(\omega_H)}/\partial\omega_H = [\mathbf{T}_{\mathbf{k}}^{\dagger}\mathbf{T}_{\mathbf{k}}]_{nn}, \quad \text{(S345)}$$

where ΔH is the change of the magnetic field, and Eq. (S264) was used in the second equality of the second part. Here, we note that when there are only particle-hole channel interactions between different magnon modes [non-diagonal matrix $\mathbf{A}_{\mathbf{k}}$ in Eq. (S191)] and no particle-particle channel interactions [non-diagonal matrix $\mathbf{B}_{\mathbf{k}}$ in Eq. (S191)], the paraunitary matrix $\mathbf{T}_{\mathbf{k}}$ is block diagonal

$$\mathbf{T}_{\mathbf{k}} = \begin{bmatrix} \mathbf{P}_{\mathbf{k}} & \mathbf{O} \\ \mathbf{O} & (\mathbf{P}_{-\mathbf{k}})^* \end{bmatrix}, \quad \text{(S346)}$$

where $\mathbf{P}_{\mathbf{k}}$ is a unitary matrix. For this case, the first part of Eq. (S345) is satisfied as $[\mathbf{T}_{\mathbf{k}}^{\dagger}\mathbf{T}_{\mathbf{k}}]_{nn} = 1$. We also note that the base-field-independent expression of the second part of Eq. (S341) [i.e. $g_{\mathbf{k},n}^{(\omega_H+\omega_{\Delta H})} \approx g_{\mathbf{k},n}^{(\omega_H)}$] is satisfied as a zeroth-order approximation for any conditions. Therefore, Eq. (S341) is justified when there are no particle-particle channel interactions for magnon modes probed by the NV centers (i.e. interacting with large enough couplings) is not significantly large. This holds for a wide variety of mangon modes in general.

For example, the approximation Eq. (S341) is partially justified or more reliable under any of the following three conditions. Firstly, Eq. (S341) is justified for the contribution arising from the plateau region of the MSSW mode ($k \gg 1/d$ and $D_{\text{ex}} \to 0$),as $\omega_0(\mathbf{k}) \approx \omega_H + \omega_M/2$ and the coupling strength

$g_k^{(\mathrm{MSSW})}$ is field independent [see Eqs. (S145) and (S144)]. Secondly, the first part of Eq. (S341) is satisfied for the exchange spin waves (i.e. the spin waves in the limit $D_{\mathrm{ex}}k^2 \gg \omega_H, \omega_M$), for which the dispersion relation is given by (*27*) $\omega(\mathbf{k}) = \left[(\omega_H + D_{\mathrm{ex}}k^2)(\omega_H + D_{\mathrm{ex}}k^2 + \omega_M \mathrm{sin}^2\theta)\right]^{1/2} \approx \omega_H + D_{\mathrm{ex}}k^2 + (\omega_M/2)\mathrm{sin}^2\theta$. Lastly, when the NV-center's main symmetry axis is parallel to the external magnetic field, the second part of Eq. (S341) is more reliable because the change in the NV-magnon coupling strength due to the change of the NV eigenstates does not happen.

Importantly, Eq. (S334) is supported by Fig. 2C in the main text without resorting to Eq. (S341). For the convenience, we consider the following base-magnetic-field independent expression of Eq. (S334)

$$\chi_{B_-B_+(\omega_H+\omega_{\Delta H})}[\omega] \approx \chi_{B_-B_+(\omega_H)}[\omega - \omega_{\Delta H}]. \tag{S347}$$

In Fig. 2C we show the noise spectrum $\mathcal{S}(\omega) \equiv \mathcal{S}_{B_-B_+(\omega_H)}[\omega]$ under two field conditions, from where we see that the effect of the magnetic field appears mostly to shift the noise spectrum. Here, the noise spectrum is defined by

$$\mathcal{S}_{B_-B_+(\omega_H)}[\omega] \equiv iG^K_{B_-B_+(\omega_H)}[\omega]/2 = \chi''_{B_-B_+(\omega_H)}[\omega]\mathrm{coth}(\beta\omega/2), \tag{S348}$$

where we used the FDR Eq. (S302) in the second equality. Based on the observation of Fig. 2C, it appears

$$\mathcal{S}_{B_-B_+(\omega_H+\omega_{\Delta H})}[\omega] \approx \mathcal{S}_{B_-B_+(\omega_H)}[\omega - \omega_{\Delta H}]. \tag{S349}$$

Using Eq. (S348), the above observation can be transformed into

$$\chi''_{B_-B_+(\omega_H+\omega_{\Delta H})}[\omega] \approx \chi''_{B_-B_+(\omega_H)}[\omega - \omega_{\Delta H}], \tag{S350}$$

where the dependency associated with the factor $\mathrm{coth}(\beta\omega/2)$ in Eq. (S348) can be ignored in the frequency range and the amount of the field change ΔH shown in Fig. 2C. This shows that the imaginary part of Eq. (S347) is supported by Fig. 2C in the main text.

While Eq. (S350) validates only the imaginary part of Eq. (S347), with the use of the KKR

Eq. (S333), the same relation holds for the real part. More concretely, we obtain

$$\begin{aligned}\chi'_{B_-B_+(\omega_H+\omega_{\Delta H})}[\omega] &= -\mathcal{P}\int_{-\infty}^{\infty}\frac{d\omega'}{\pi}\frac{\chi''_{B_-B_+(\omega_H+\omega_{\Delta H})}[\omega']}{\omega-\omega'},\\ &\approx -\mathcal{P}\int_{-\infty}^{\infty}\frac{d\omega'}{\pi}\frac{\chi''_{B_-B_+(\omega_H)}[\omega'-\omega_{\Delta H}]}{\omega-\omega'},\\ &= -\mathcal{P}\int_{-\infty}^{\infty}\frac{d\omega'}{\pi}\frac{\chi''_{B_-B_+(\omega_H)}[\omega']}{\omega-\omega_{\Delta H}-\omega'} = \chi'_{B_-B_+(\omega_H)}[\omega-\omega_{\Delta H}], \end{aligned} \tag{S351}$$

where in the first line we used the KKR, in the second line we used Eq. (S350), in the third line we changed the integration variable, and in the last equality we again used the KKR. This indicates that imposing only the imaginary part of Eq. (S347) is equivalent to imposing both the real and the imaginary part of Eq. (S347) thanks to the causality of the susceptibility.

SIV.2 Derivation of the Lindblad master equation for multi-qubit case

In this section, we show the two-qubit case of the derivation of the effective time evolution of the qubit density matrix ρ in the Lindblad master equation formalism, and obtain the effective NV-NV coupling strength. We take the initial total Hamiltonian to be that of the two qubits coupled to multi-mode boson (magnon) bath:

$$\mathcal{H} = \mathcal{H}_0 + \mathcal{H}_B + V, \tag{S352}$$

$$\mathcal{H}_0 = \sum_{i=1,2}\frac{\omega_s}{2}\sigma_z^{(i)}, \tag{S353}$$

$$\mathcal{H}_B = \sum_j \omega_j b_j^\dagger b_j, \tag{S354}$$

$$V = \sum_{i=1,2} B_-^{(i)}\sigma_+^{(i)} + B_+^{(i)}\sigma_-^{(i)};\quad B_-^{(i)} \equiv \sum_j g_j^{(i)} b_j;\quad B_+^{(i)} = \left(B_-^{(i)}\right)^\dagger. \tag{S355}$$

In the same way as in Sec. SIII.6, under the Born-Markov approximation and for the thermal boson bath which is stationary, and assuming $\langle B_-^{(i_1)}(t)B_-^{(i_2)}(0)\rangle = \langle B_+^{(i_1)}(t)B_+^{(i_2)}(0)\rangle = 0$ which is valid in our case (or applying the secular approximation), we obtain the equation of motion for the reduced

qubit density operator ρ as

$$\frac{d}{dt}\rho(t) = -i\left[\mathcal{H}_0 + \mathcal{H}_{\text{eff}}, \rho\right] + \mathcal{N}[\rho(t)], \tag{S356}$$

$$\mathcal{H}_{\text{eff}} = -\left(g_{\text{eff}}\sigma_+^{(1)}\sigma_-^{(2)} + g_{\text{eff}}^*\sigma_+^{(2)}\sigma_-^{(1)}\right) + \sum_{i=1,2}\left(\Sigma_{\text{Lamb}}^{(i)}\sigma_+^{(i)}\sigma_-^{(i)} + \Sigma_{\text{Stark}}^{(i)}\sigma_z^{(i)}\right), \tag{S357}$$

$$\mathcal{N}[\rho(t)] = \sum_{i_1,i_2} iG^<_{B_-^{(i_1)}B_+^{(i_2)}}\left[\omega_s\right]\mathcal{L}\left[\sigma_+^{(i_1)}, \sigma_+^{(i_2)}\right]\rho + \sum_{i_1,i_2} iG^>_{B_-^{(i_1)}B_+^{(i_2)}}\left[\omega_s\right]\mathcal{L}\left[\sigma_-^{(i_2)}, \sigma_-^{(i_1)}\right]\rho, \tag{S358}$$

where the Lamb shift $\Sigma_{\text{Lamb}}^{(i)}$, the Stark shift $\Sigma_{\text{Stark}}^{(i)}$, and the effective coupling g_{eff} are defined by

$$\Sigma_{\text{Lamb}}^{(i)} = -\chi'_{B_-^{(i)}B_+^{(i)}}\left[\omega_s\right] = \sum_j \mathcal{P}\frac{\left|g_j^{(i)}\right|^2}{\omega_s - \omega_j}, \tag{S359}$$

$$\Sigma_{\text{Stark}}^{(i)} = \int_{-\infty}^{\infty}\frac{d\omega}{2\pi}\mathcal{P}\frac{iG^<_{B_-B_+}[\omega]}{\omega_s - \omega} = \sum_j \mathcal{P}\frac{\left|g_j^{(i)}\right|^2 n_B(\omega_j)}{\omega_s - \omega_j}, \tag{S360}$$

$$g_{\text{eff}} = \chi'_{B_-^{(1)}B_+^{(2)}}\left[\omega_s\right] = -\sum_j \mathcal{P}\frac{g_j^{(2)*}g_j^{(1)}}{\omega_s - \omega_j}. \tag{S361}$$

We note that both in Eqs. (S359) and (S360), the prime of the susceptibility indicates the first part we defined in Eqs. (S313) and (S314), while in Eq. (S359) it is also the same as the real part, which is consistent with Eq. (S285). We also defined the two-argument Lindblad operator

$$\mathcal{L}[X, Y]\rho \equiv X\rho Y^\dagger - \frac{1}{2}\left\{Y^\dagger X, \rho\right\}, \tag{S362}$$

such that when the two arguments are the same, it reduces to the standard Lindblad operator $\mathcal{L}[X, X] = \mathcal{L}[X]$. The greater and the lesser Green's function in Eq. (S358) can be explicitly written as

$$iG^>_{B_-^{(i_1)}B_+^{(i_2)}}\left[\omega_s\right] = \left(n_B\left(\omega_s\right) + 1\right)\sum_j g_j^{(i_2)*}g_j^{(i_1)}2\pi\delta\left(\omega_s - \omega_j\right), \tag{S363}$$

$$iG^<_{B_-^{(i_1)}B_+^{(i_2)}}\left[\omega_s\right] = n_B\left(\omega_s\right)\sum_j g_j^{(i_2)*}g_j^{(i_1)}2\pi\delta\left(\omega_s - \omega_j\right). \tag{S364}$$

We note in Eq. (S358) that the terms in the summation with $i_1 = i_2$ are essentially the same as what we obtained in the single qubit case [Eq. (S288)]:

$$\mathcal{N}[\rho(t)]\big|_{i_1=i_2} = \sum_i\left(iG^<_{B_-^{(i)}B_+^{(i)}}\left[\omega_s\right]\mathcal{L}\left[\sigma_+^{(i)}\right]\rho + iG^>_{B_-^{(i)}B_+^{(i)}}\left[\omega_s\right]\mathcal{L}\left[\sigma_-^{(i)}\right]\rho\right). \tag{S365}$$

From Eqs. (S360) and (S361), we find that when two qubits (NVs) are close and when we can take $B_\pm^{(1)} = B_\pm^{(2)} \equiv B_\pm$, the effective coupling equals to the real part of the susceptibility (self-energy shift or Lamb shift):

$$g_{\text{eff}} \to \chi'_{B_+B_+}[\omega_s] = -\Sigma_{\text{Lamb}}. \tag{S366}$$

This is not a coincident, and in fact under this condition the effective Hamiltonian Eq. (S357) becomes

$$\mathcal{H}_{\text{eff}} \to -\chi'_{B_-B_+}[\omega_s]\left(\sigma_+^{(1)} + \sigma_+^{(2)}\right)\left(\sigma_-^{(1)} + \sigma_-^{(2)}\right) + \Sigma_{\text{Stark}}\left(\sigma_z^{(1)} + \sigma_z^{(2)}\right), \tag{S367}$$

which can be derived from Eq. (S284) by replacing $\sigma_\pm \to \sigma_\pm^{(1)} + \sigma_\pm^{(2)}$ and $\sigma_z \to \sigma_z^{(1)} + \sigma_z^{(2)}$, i.e.

$$\begin{aligned}\mathcal{H}_{\text{eff}} &= \Sigma_{\text{Lamb}}\sigma_+\sigma_- + \Sigma_{\text{Stark}}\sigma_z, \\ &\to \Sigma_{\text{Lamb}}\left(\sigma_+^{(1)} + \sigma_+^{(2)}\right)\left(\sigma_-^{(1)} + \sigma_-^{(2)}\right) + \Sigma_{\text{Stark}}\left(\sigma_z^{(1)} + \sigma_z^{(2)}\right).\end{aligned} \tag{S368}$$

In fact, the derivation of the Lindblad master equation with a single qubit case shown in Sec. SIII.6 can be repeated by replacing $\sigma_\pm \to \sigma_\pm^{(1)} + \sigma_\pm^{(2)}$ and $\sigma_z \to \sigma_z^{(1)} + \sigma_z^{(2)}$ to obtain the above result Eq. (S368). As the effective coupling typically decays as a function of distance (such as Refs. (*14*) , (*43*), and other boson-mediated interactions), Eq. (S366) indicates that the real part of the susceptibility $\chi'_{B_-B_+}$ provides an upper bound estimate of the effective coupling mediated by magnons.

SIV.2.1 correlated decay in the Lindblad operator

There are also additional terms in Eq. (S358), such as the terms with $\mathcal{L}[\sigma_-^{(1)}, \sigma_-^{(2)}]$. We call these as correlated decay dissipation terms, as they are related to operators such as $\mathcal{L}[\sigma_-^{(1)} + \sigma_-^{(2)}]$. For example,

$$\mathcal{L}\left[\sigma_-^{(1)} + \sigma_-^{(2)}\right] = \mathcal{L}\left[\sigma_-^{(1)}\right] + \mathcal{L}\left[\sigma_-^{(2)}\right] + \mathcal{L}\left[\sigma_-^{(1)}, \sigma_-^{(2)}\right] + \mathcal{L}\left[\sigma_-^{(2)}, \sigma_-^{(1)}\right]. \tag{S369}$$

Noticing that $\mathcal{L}[\sigma_-^{(1)} + \sigma_-^{(2)}]$ describes the decay process of $|e_1e_2\rangle \to (|g_1e_1\rangle + |e_1g_2\rangle)/\sqrt{2}$, it makes sense to call its effect as the correlated decay dissipation.

Interestingly, the time scale of the correlated decay process are related to the qubit-qubit interaction via the fluctuation-dissipation and the Kramers-Kronig relation, which can be shown through a

similar argument as that provided in Sec. SIII.6.1 where the T1 decay rate is related to the Lamb shift. We define the rate of the correlated decay process as

$$1/T_1^{(\mathrm{corr})} = iG^{>}_{B_-^{(1)}B_+^{(2)}}[\omega_s] + iG^{<}_{B_-^{(1)}B_+^{(2)}}[\omega_s] = iG^{K}_{B_-^{(1)}B_+^{(2)}}[\omega_s], \tag{S370}$$

where $G^{K}_{B_-^{(1)}B_+^{(2)}}[\omega_s]$ is the Keldysh Green's function defined in Eq. (S295). Using the fluctuation-dissipation relation [Eq. (S308) with zero chemical potential $\mu = 0$] and the definition of the second part of the susceptibility [Eq. (S314)], we obtain in analogy to Eq. (S302) the following:

$$\begin{aligned} 1/T_1^{(\mathrm{corr})} &= iG^{K}_{B_-^{(1)}B_+^{(2)}}[\omega_s] = A_{B_-^{(1)}B_+^{(2)}}[\omega_s]\coth[\beta\omega_s/2], \\ &= 2\chi''_{B_-^{(1)}B_+^{(2)}}[\omega_s]\coth[\beta\omega_s/2]. \end{aligned} \tag{S371}$$

Therefore, the correlated decay rate $1/T_1^{(\mathrm{corr})}$ is proportional to the second part (not imaginary part) of the susceptibility, which should be seen in contrast to the effective qubit-qubit coupling g_{eff} provided in Eq. (S361), which is the first part of the susceptibility. Using the Kramers-Kronig relation shown in Eq. (S315), we obtain the relation between them with the assumption that we have access to the frequency dependence $T_1^{(\mathrm{corr})} = T_1^{(\mathrm{corr})}[\omega]$:

$$g_{\mathrm{eff}} = \chi'_{B_-^{(1)}B_+^{(2)}}[\omega_s] = -\mathcal{P}\int_{-\infty}^{\infty}\frac{d\omega'}{\pi}\frac{1}{\omega_s-\omega'}\cdot\frac{1/T_1^{(\mathrm{corr})}[\omega']}{2\coth[\beta\omega'/2]}. \tag{S372}$$

This equation clearly shows the connection of the correlated decay rates and the effective qubit-qubit interaction in analogy to Eq. (S305).

SV Analytical evaluation of the effective coupling

In this section, we calculate the r dependence of $g_{\mathrm{eff}}(r)$ analytically, which is given by Eq. (S340). The exact evaluation of Eq. (S340), however, turns out to be difficult because it includes the summation over all the thickness-directional modes n (such as the MSSW-like modes and volume-like modes), in addition to the integral over the in-plane wave vector $\mathbf{k}$. In Fig. 4B in the main text, we therefore evaluated Eq. (S340) numerically. To obtain the corresponding analytical expression, we use the fact that the major contribution arises from the MSSW-like mode, with approximated expression

of their coupling and dispersion relation provided by Eqs. (S25) and (S26), respectively. Defining $\varphi_{\mathbf{k}} = \phi_{\mathbf{k}} - \pi/2$, they become

$$\begin{aligned} g_{\mathbf{k}} &= \frac{\gamma\mu_0}{\sqrt{2}}\sqrt{k\gamma M_{\rm s}}e^{-kh_{\rm NV}}\cos^2\varphi_{\mathbf{k}}\mathbb{I}_{|\varphi_{\mathbf{k}}|\leq\pi/2}, \\ &= \sqrt{k\mu_0\gamma^2\omega_M/2}e^{-kh_{\rm NV}}\cos^2\varphi_{\mathbf{k}}\mathbb{I}_{|\varphi_{\mathbf{k}}|\leq\pi/2}, \end{aligned} \tag{S373}$$

$$\begin{aligned} \omega_{\mathbf{k}} &= \omega_H + (\omega_M/2)\cos^2\varphi_{\mathbf{k}} + D_{\rm ex}k^2, \\ &= \omega_{\rm p} - (\omega_M/2)\sin^2\varphi_{\mathbf{k}} + D_{\rm ex}k^2, \end{aligned} \tag{S374}$$

where we use $\omega_M = \gamma\mu_0 M_{\rm s}$ and $\omega_{\rm p} = \omega_H + (\omega_M/2)$. Under this single thickness-mode assumption, the effective coupling Eq. (S340) becomes

$$\begin{aligned} g_{\rm eff}(r) &= \mathcal{P}\int\frac{d\mathbf{k}}{(2\pi)^2}|g_{\mathbf{k}}|^2\frac{e^{i(\mathbf{k}\cdot\hat{z})r}}{\omega_{\mathbf{k}} - \omega_{\rm NV}}, \\ &= \frac{1}{4\pi^2}\mathcal{P}\int_0^\infty dkk\int_{-\pi}^{\pi} d\varphi_{\mathbf{k}}|g_{\mathbf{k}}|^2\frac{e^{ikr\sin\varphi_{\mathbf{k}}}}{\omega_{\mathbf{k}} - \omega_{\rm NV}}, \end{aligned} \tag{S375}$$

where we write $\mathbf{r}_{{\rm NV}_1} - \mathbf{r}_{{\rm NV}_2} = r\hat{z}$. Note that we are displacing two NV centers along the $\hat{z} \propto \mathbf{H}$ direction in Fig. 4B, i.e., perpendicular to the MSSW propagation direction. Eq. (S375) is basically the inverse Fourier transform of $|g_{\mathbf{k}}|^2/(\omega_{\mathbf{k}} - \omega_{\rm NV})$. In the following paragraphs, we evaluate Eq. (S375) under two different cases.

Firstly, we consider the case where the wave-vector $\mathbf{k}$ dependence of $|g_{\mathbf{k}}|^2/(\omega_{\mathbf{k}} - \omega_{\rm NV})$ is dominated by the numerator $|g_{\mathbf{k}}|^2$, and we can ignore the $\mathbf{k}$-dependence in the denominator $1/(\omega_{\mathbf{k}} - \omega_{\rm NV})$. In this situation, we can approximate $\omega_{\mathbf{k}} \approx \omega_{\rm p}$. Then, Eq. (S375) becomes

$$\begin{aligned} g_{\rm eff}(r) &\approx \frac{1}{4\pi^2(\omega_{\rm p} - \omega_{\rm NV})}\int_0^\infty dkk\int_{-\pi/2}^{\pi/2} d\varphi_{\mathbf{k}}\left(\frac{k\mu_0\gamma^2\omega_M\cos^4\varphi_{\mathbf{k}}}{2}e^{-2kh_{\rm NV}}\right)e^{ikr\sin\varphi_{\mathbf{k}}}, \\ &= -\frac{\mu_0\gamma^2}{8\pi^2\Delta_{\rm NV}}\int_0^\infty dkk^2e^{-2kh_{\rm NV}}\int_{-\pi/2}^{\pi/2} d\varphi_{\mathbf{k}}\cos^4\varphi_{\mathbf{k}}e^{ikr\sin\varphi_{\mathbf{k}}}, \\ &= -\frac{3\mu_0\gamma^2}{8\pi r^2\Delta_{\rm NV}}\int_0^\infty dke^{-2kh_{\rm NV}}J_2(kr), \\ &= -\frac{\mu_0\gamma^2}{4\pi r^3}\cdot\frac{3}{2\Delta_{\rm NV}}\int_0^\infty dxe^{-bx}J_2(x), \\ &= -\frac{\mu_0\gamma^2}{4\pi r^3}\cdot\frac{3}{2\Delta_{\rm NV}}\left(\frac{1}{\sqrt{b^2+1}} - 2b + \frac{2b^2}{\sqrt{b^2+1}}\right), \end{aligned} \tag{S376}$$

where we define the dimensionless detuning $\Delta_{\rm NV} = (\omega_{\rm NV} - \omega_{\rm p})/\omega_M$ in the second line, we use $\int_{-\pi/2}^{\pi/2} d\varphi\cos^4\varphi e^{ix\sin\varphi} = 3\pi J_2(x)/x^2$ with $x = kr$ and the Bessel function of the first kind $J_\nu(\cdots)$

in the third line, $b = 2h_{\rm NV}/r$ in the forth line, and $\int_0^\infty dx e^{-bx} J_2(x) = \frac{1}{\sqrt{b^2+1}} - 2b + \frac{2b^2}{\sqrt{b^2+1}}$ in the last line. For a small r regime, we have $\frac{1}{\sqrt{b^2+1}} - 2b + \frac{2b^2}{\sqrt{b^2+1}} \approx \frac{1}{4b^3}$ for $b = 2h_{\rm NV}/r \gg 1$ (i.e., $r \ll h_{\rm NV}$), which leads to

$$g_{\rm eff}(r) \approx -\frac{\mu_0\gamma^2}{4\pi h_{\rm NV}^3}\frac{3}{64\Delta_{\rm NV}} = g_{\rm dip}\left(r = h_{\rm NV}\right)\frac{3}{64\Delta_{\rm NV}}, \tag{S377}$$

where $g_{\rm dip}(r) = -\mu_0\gamma^2/4\pi r^3$ is the dipole coupling. In contrast, when r is large, we have $\frac{1}{\sqrt{b^2+1}} - 2b + \frac{2b^2}{\sqrt{b^2+1}} \approx 1$ for $b = 2h_{\rm NV}/r \ll 1$ (i.e., $r \gg h_{\rm HV}$), which leads to

$$g_{\rm eff}(r) \approx -\frac{\mu_0\gamma^2}{4\pi r^3}\frac{3}{2\Delta_{\rm NV}} = g_{\rm dip}(r)\frac{3}{2\Delta_{\rm NV}}. \tag{S378}$$

This indicates that, for a large r, the scaling of $g_{\rm eff}(r)$ is the same as that of $g_{\rm dip}(r)$, but with a larger prefactor $3/2\Delta_{\rm NV}$. For example, near the experimental range shown in Fig. 4B, we have $(\omega_{\rm NV} - \omega_{\rm p})/\gamma \approx 5$ G and $\omega_M/\gamma = 1716$ G, which results in $\Delta_{\rm NV} = (\omega_{\rm NV} - \omega_{\rm p})/\omega_M \approx 1/350$ and therefore $3/2\Delta_{\rm NV} \approx 500 \gg 1$.

Next, we consider a different case where the wave-vector $\mathbf{k}$ dependence of $|g_{\mathbf{k}}|^2/(\omega_{\mathbf{k}} - \omega_{\rm NV})$ is dominated by the denominator. We assume $g_{\mathbf{k}}$ can be treated as a constant $g_{\mathbf{k}} \approx g_0$. Furthermore, for the simplicity of the computation, we consider the case with $\omega_{\rm NV} < \omega_{\rm p}$, and assume that the dependence of $\omega_{\mathbf{k}}$ on $\mathbf{k}$ arises from the exchange energy $D_{\rm ex}k^2$. In such cases, Eq. (S375) becomes

$$\begin{aligned} g_{\rm eff}(r) &\approx \frac{|g_0|^2}{4\pi^2}\int_0^\infty dkk\int_{-\pi}^{\pi} d\varphi_{\mathbf{k}}\frac{e^{ikr\sin\varphi_{\mathbf{k}}}}{D_{\rm ex}k^2 + (\omega_{\rm p} - \omega_{\rm NV})}, \\ &= \frac{|g_0|^2}{4\pi^2}\int_0^\infty dk\frac{k}{D_{\rm ex}k^2 + \omega_M\left|\Delta_{\rm NV}\right|}\int_{-\pi}^{\pi} d\varphi_{\mathbf{k}} e^{ikr\sin\varphi_{\mathbf{k}}}, \\ &= \frac{|g_0|^2}{4\pi^2}\int_0^\infty dk\frac{k}{D_{\rm ex}k^2 + \omega_M\left|\Delta_{\rm NV}\right|}\cdot 2\pi J_0(kr), \\ &= \frac{|g_0|^2}{2\pi D_{\rm ex}}\int_0^\infty dx\frac{x}{x^2+1}\cdot J_0(bx), \\ &= \frac{|g_0|^2}{2\pi D_{\rm ex}}K_0(\kappa r), \end{aligned} \tag{S379}$$

where we use $\int_{-\pi}^{\pi} d\varphi_{\mathbf{k}} e^{ix\sin\varphi_{\mathbf{k}}} = 2\pi J_0(x)$ in the third line, $x = k/\kappa$, $\kappa = \sqrt{|\Delta_{\rm NV}|}/a_{\rm ex}$, and $b = \kappa r$ in the forth line, and $\int_0^\infty dx J_0(bx)\cdot\frac{x}{x^2+1} = K_0(b)$ with the modified Bessel function of the second kind $K_\nu(\cdots)$ in the last line. For a small r regime, we can approximate $K_0(b) \approx -\ln(b)$ for $b = \kappa r \ll 1$,

which leads to

$$g_{\text{eff}}(r) \approx -\frac{|g_0|^2}{2\pi D_{\text{ex}}} \ln(\kappa r). \tag{S380}$$

In the opposite limit $b = \kappa r \gg 1$, we can approximate $K_0(b) \approx (\pi/2b)^{-1/2} e^{-b}$, which leads to

$$g_{\text{eff}}(r) \approx \frac{|g_0|^2}{2\pi D_{\text{ex}}} \sqrt{\frac{\pi}{2\kappa r}} e^{-\kappa r}. \tag{S381}$$

We note that $K_0(\kappa r)$ is basically the Green's function for a massive Laplace's equation (or a screened Poisson's equation) $(-\nabla^2 + \kappa^2)G(\mathbf{r}) = \delta(\mathbf{r})$ in $d = 2$ dimension. In $d = 1$ and $d = 3$ dimensions, we obtain $G(r) \propto e^{-\kappa r}$ and $G(r) \propto e^{-\kappa r}/r$, respectively, as discussed in Ref. (*43*). For the coupling g_0, as a reference, we may use

$$g_0 = \max_{\mathbf{k}} g_{\mathbf{k}} = g_{\mathbf{k}}|_{k=1/2h_{\text{NV}}, \varphi_{\mathbf{k}}=0} = \sqrt{\mu_0 \gamma^2 \omega_M / 4e h_{\text{NV}}}, \tag{S382}$$

where $e = \exp(1)$ is the Napier number and should not be confused with the electron charge. In this case, Eq. (S379) becomes

$$g_{\text{eff}}(r) = \frac{\mu_0 \gamma^2}{4\pi h_{\text{NV}} a_{\text{ex}}^2} \cdot \frac{1}{2e} K_0(\kappa r) = \left| g_{\text{dip}} \left(r = \left(h_{\text{NV}} a_{\text{ex}}^2 \right)^{1/3} \right) \right| \cdot \frac{1}{2e} K_0(\kappa r). \tag{S383}$$

Here, we note that the prefactor is the dipole interaction at a distance of $r = (h_{\text{NV}} a_{\text{ex}}^2)^{1/3}$. As for the decay constant κ, for the experimental condition $\Delta_{\text{NV}} = 1/350$ discussed in the previous paragraph, we obtain $\kappa = \sqrt{|\Delta_{\text{NV}}|}/a_{\text{ex}} \approx 1/(300\ \text{nm})$. Using Eq. (S383), the condition $g_{\text{eff}}(r) \gtrsim |g_{\text{dip}}(r)|$ is equivalent to $(\kappa r)^3 K_0(\kappa r) \gtrsim 2e(h_{\text{NV}}/a_{\text{ex}})|\Delta_{\text{NV}}|^{3/2} \approx 0.02$, which is satisfied for $0.2 \lesssim \kappa r \lesssim 10$ or $150\ \text{nm} \lesssim r \lesssim 3\ \mu\text{m}$.

As shown in the analysis above, the effective coupling $g_{\text{eff}}(r)$ scales as $g_{\text{eff}}(r) \propto 1/r^3$ with a large prefactor or as $g_{\text{eff}}(r) \propto e^{-\kappa r}/\sqrt{r}$ with a decay constant κ, depending on the approximations used. In both cases, the coupling g_{eff} is larger than the dipole coupling g_{dip} within the range of r shown in Fig. 4B.

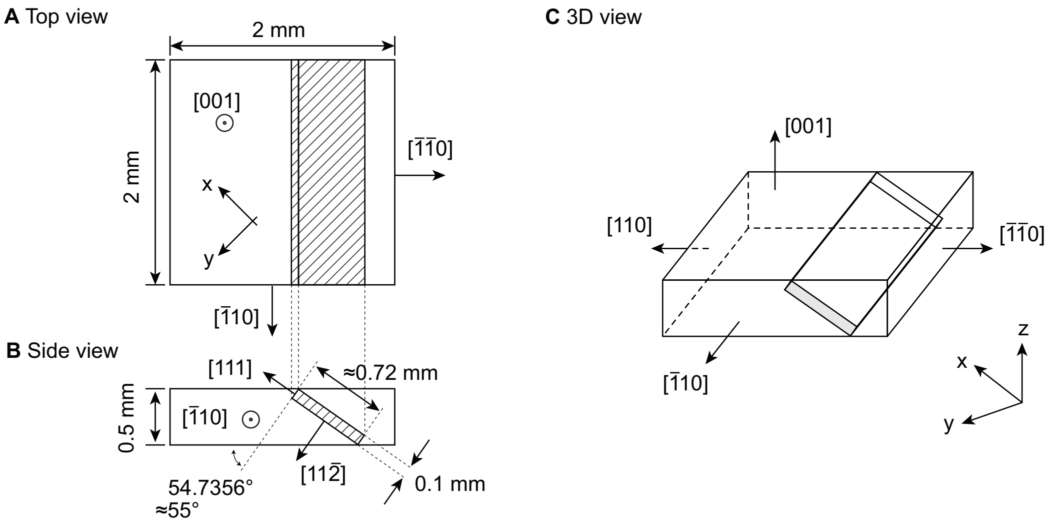

Figure S1: **Diamond slab processing and the crystal orientation.** (**A**) Top view of a 2 mm $\times$ 2 mm $\times$ 0.5 mm diamond crystal. The shaded area represents the part that will be cut into a diamond slab. (**B**) Side view of the diamond crystal shown in (A), where the shaded area represents the part thaw will be cut into the diamond slab. (**C**) Three-dimensional view of the diamond crystal shown in (A) with the visualization of the part that will be laser cut into the slab.

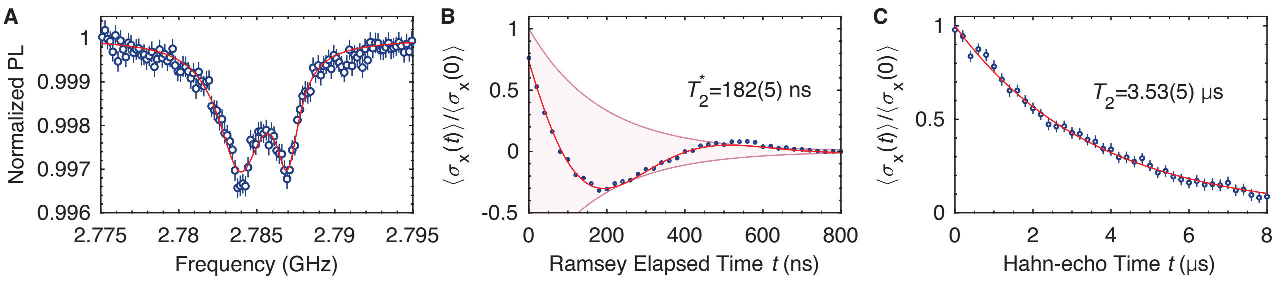

Figure S2: **Coherence of an ensemble of NV centers used in the study.** (**A**) Pulsed ODMR spectrum of an ensemble of NV centers for the transition $|m_s = 0\rangle \leftrightarrow |m_s = -1\rangle$ with no YIG coverage (see Fig. S3A). An external magnetic field (30 G) is applied parallel to the NV axis. The two dips correspond to the $I = 1/2$ nuclear spin of the implanted nitrogen [^{15}N]. The red curve represents the fit using two Lorentzian functions. (**B**) Ramsey spectroscopy of the ensemble NV centers with no YIG coverage, showing the dephasing time $T_2^* = 182(5)$ ns. The curve is fit by an exponential function multiplied by a cosine function with a phase offset to account for a finite pulse time and imperfect pulse shapes [i.e. $\propto \cos(\omega t + \phi)\exp(-t/T_2^*)$]. (**C**) Hahn-echo spectroscopy of the ensemble NV centers with no YIG coverage, showing decoherence time $T_2 = 3.53(5)$ μs. The curve is fit by an exponential function [$\propto \exp(-t/T_2)$].

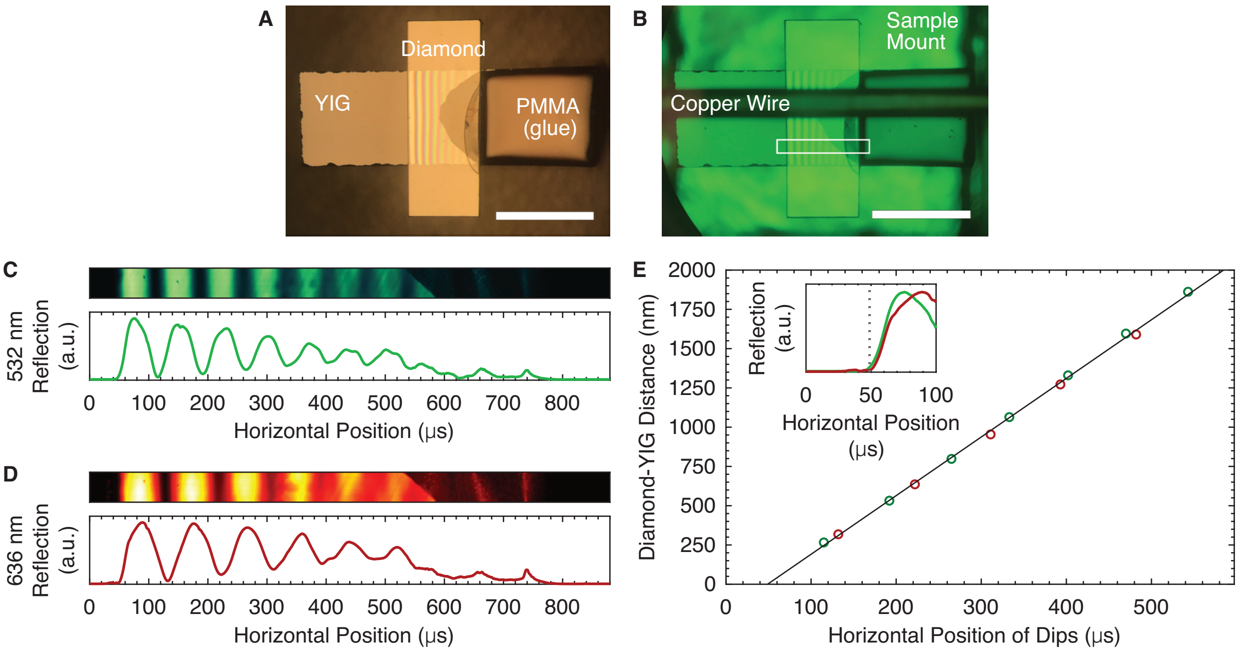

Figure S3: **Calibration of the diamond-YIG distance.** (**A**) Microscope image of the diamond slab (0.7 mm $\times$ 2 mm $\times$ 0.1 mm) placed on top of a YIG film (3 mm $\times$ 1 mm $\times$ 3 μm) [on a GGG substrate (3 mm $\times$ 1 mm $\times$ 0.5 mm)] under a white light. Vertical optical interference fringes are prominent especially near the left side, showing that the left side has smaller diamond-YIG distance. The dark colored area on the right is the PMMA used as a glue. Scale bar: 1 mm. (**B**) Microscope image of the YIG-diamond hybrid structure after placing a copper wire over the sample for applying microwaves and mounting the sample onto an aluminium sample mount, under a green monochromatic light. Clear optical interference fringes are visible as we use the monochromatic light. Optical reflectivity measurements are performed near the white squared position. Scale bar: 1 mm. (**C**) Optical interference measured by a reflectivity under a 532-nm (green) laser illumination (top) and the horizontal position dependence of the reclection (bottom). (**D**) Optical interference measured by a reflectivity under a 636-nm (red) laser illumination (top) and the horizontal position dependence of the reflection (bottom). (**E**) Calculated diamond-YIG distance versus the horizontal position of the destructive optical interference fringes. The green (red) marker represents the destructive interference of 532-nm (636-nm) laser reflection. Inset shows the reflection of green and red lasers near the left edge of the diamond slab [(Horizontal Position) $\approx$ 50 μm, see the vertical dotted line] providing expected zero diamond-YIG distance.

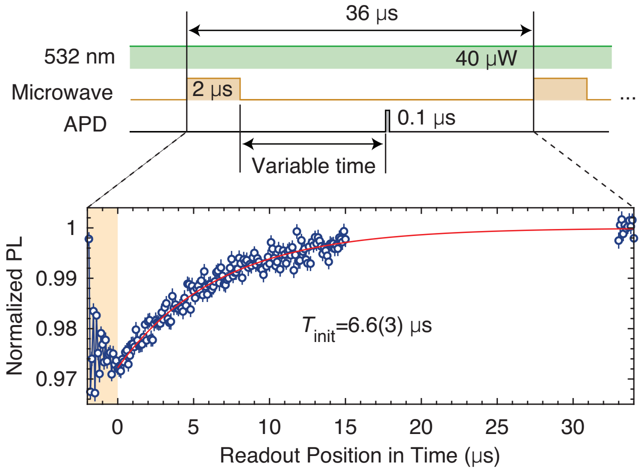

Figure S4: **Simplified initialization-time measurement of the NV centers.** The measurement is taken at $P_{\mathrm{L}} = 40\ \mu\mathrm{W}$ green ($532\ \mathrm{nm}$) laser illumination power. The upper drawing shows the pulse sequences used in the measurement, where the pulses are repeated with a cycle of $36\ \mu$s. The microwave frequency is set to the NV transition frequency of $|m_s = 0\rangle \leftrightarrow |m_s = -1\rangle$. The APD readout position in time is varied as shown in the upper drawing (variable time), where the time origin in the lower figure is set to the end of the applied microwave pulse. The orange shaded area in the lower figure represents the time where the microwave is applied, indicating that the reduction of the normalized PL is the signature of the ODMR. The recovery of the normalized PL signal after the end of the microwave pulse shows the initialization of the NV spin states to $|m_s = 0\rangle$. The exponential fit is shown with the red curve and we obtain the initialization time $T_{\mathrm{init}} = 6.6 \pm 0.3\ \mu$s from the fit.

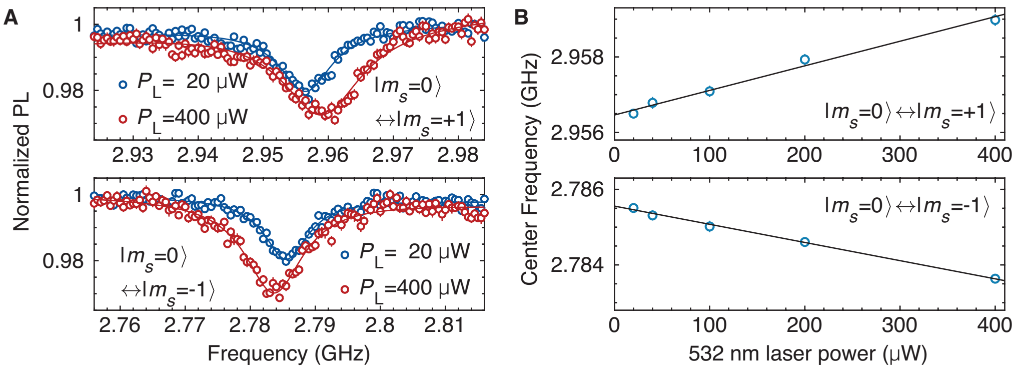

Figure S5: **Laser heating originated frequency shift of NV centers on YIG.** (**A**) Continuous-wave ODMR of the NV-center transitions $|m_s = 0\rangle \leftrightarrow |m_s = +1\rangle$ (top) and $|m_s = 0\rangle \leftrightarrow |m_s = -1\rangle$ (bottom) at two laser-power conditions $P_{\mathrm{L}} = 20\ \mu\mathrm{W}$ and $P_{\mathrm{L}} = 400\ \mu\mathrm{W}$ of the green (532 nm) laser for the NV initialization and readout shown with blue and red markers, respectively. The measurement is performed at the NV centers with the YIG coverage with $h_{\mathrm{NV}} = 400$ nm. The spot size of the focused laser is $\approx 1\ \mu\mathrm{m}$ in diameter. The solid curves are fits with corresponding colors. The PL on the vertical axis is normalized with the PL without applying the microwaves. The measurement is performed at the external magnetic field $\mu_0 H_{\parallel} = 30$ G. (**B**) 532-nm laser power P_{L} dependence of the ODMR center frequency obtained from the fits as shown in (A), for the transitions $|m_s = 0\rangle \leftrightarrow |m_s = +1\rangle$ (top) and $|m_s = 0\rangle \leftrightarrow |m_s = -1\rangle$ (bottom). The solid lines are the linear fits, where we obtain the slopes $df/dP_{\mathrm{L}} = 6.5(4)$ MHz/mW and $df/dP_{\mathrm{L}} = -4.8(2)$ MHz/mW from the upper the lower figures, respectively. The opposite slopes of the fit line indicates the shift in resonance frequency is caused by the additional external magnetic field due to the laser heating of the YIG sample instead of the change in the zero-field splitting D_{NV} due to temperature change.

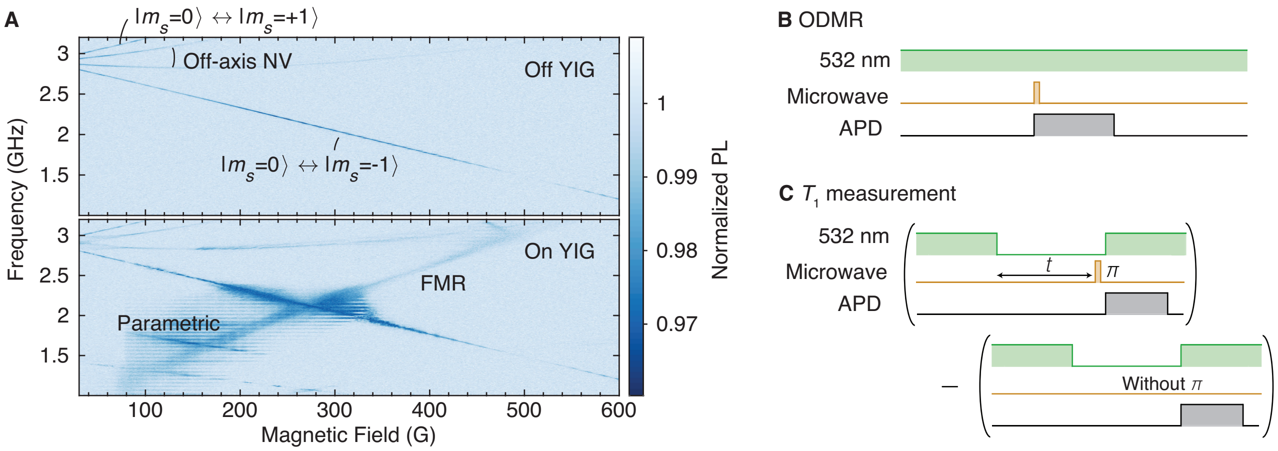

Figure S6: **ODMR comparison with and without YIG and the measurement pulse sequence.** (**A**) Heat map of the ODMR of an ensemble of NV center off (upper figure) and on (lower figure) the YIG as a function of the external magnetic field and the applied microwave frequency. The measurement off the YIG is taken on the part with no YIG coverage as shown in Fig. S3A. Multiple detailed features of the PL reduction are labeled, such as the signal coming from off-axis NV transitions, FMR, and parametric excitation of magnons in YIG. (**B**) Pulse sequences (532-nm laser, pulsed microwave tones, and the APD readout timings) used for the continuous-wave ODMR measurement. The APD readout time is $5\ \mu\mathrm{s}$ and the microwave is applied with the maximum available power 5 W for 150 ns duration. The repetition period of the measurement is $21\ \mu\mathrm{s}$. The readout pulse overlaps with the microwave pulse (also see Fig. S4). (**C**) Pulse sequences used for the T_1 measurement. The difference between the signal of the measurement with and without applying the π-pulse is fit by an exponential function $[\propto \exp(-t/T_1)]$. The π-pulses are calibrated at each external magnetic field for both power and duration. If the duration of the pulse is longer that 220 ns at the maximum microwave power 5 W, we used an approximate π-pulse, which is at the maximum microwave power 5 W and 220 ns to reduce the artifact arising from the parametrically excited magnons (*5, 8, 9*). The readout pulse does not overlap with the microwave pulse.

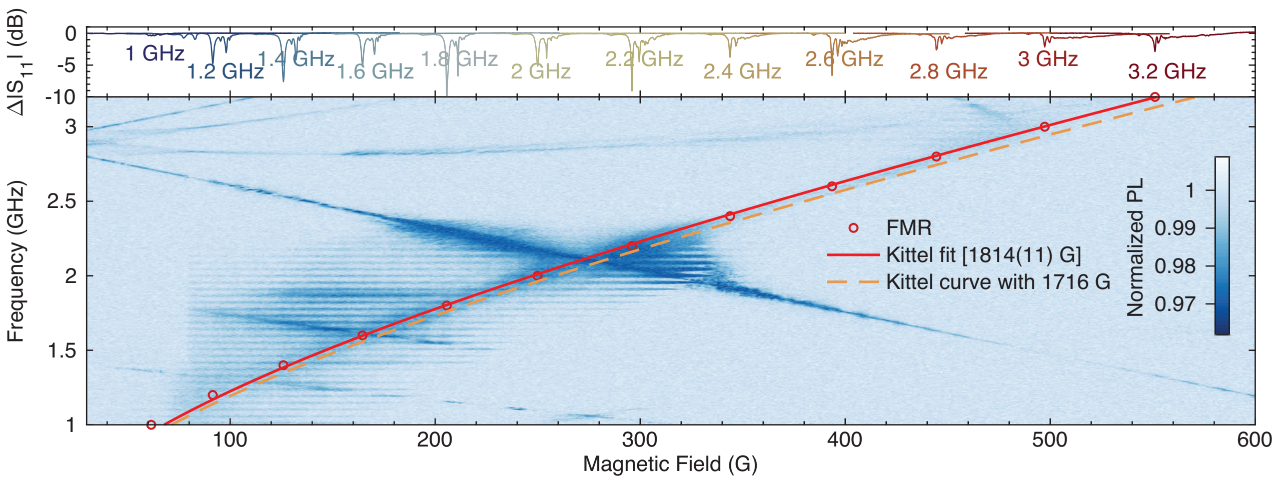

Figure S7: **Microwave refection measurement of FMR.** The top and the bottom figures represent $|S_{11}|$ and the Kittel fit, respectively. The $|S_{11}|$ measurement is performed using the same microwave transmission line (copper wire) as shown in Fig. S3B. The difference $\Delta|S_{11}|$ in signal from the flat part of the spectrum is shown to highlight the change in the reflection and the sharp dip originating from the FMR. The dip conditions (field and frequency) are shown with red markers in the lower figure on top of the heat map of the ODMR shown in Fig. S6A. The power of the microwaves used in the S_{11} measurement is minimized to $10\ \mathrm{nW}$ in order to suppress possible nonlinear phenomena (*5, 8, 9*). Kittel fit in the lower figure is shown with a solid red curve, where we obtain $M_\mathrm{s} = 1814 \pm 11\ \mathrm{G}/\mu_0$. The dashed orange curve shows the Kittel equation with the saturation magnetization parameter $M_s = 1716\ \mathrm{G}/\mu_0$ obtained by the fit of $\Delta(1/T_1)$ shown in Fig. 2B (see Sec. SI.7).

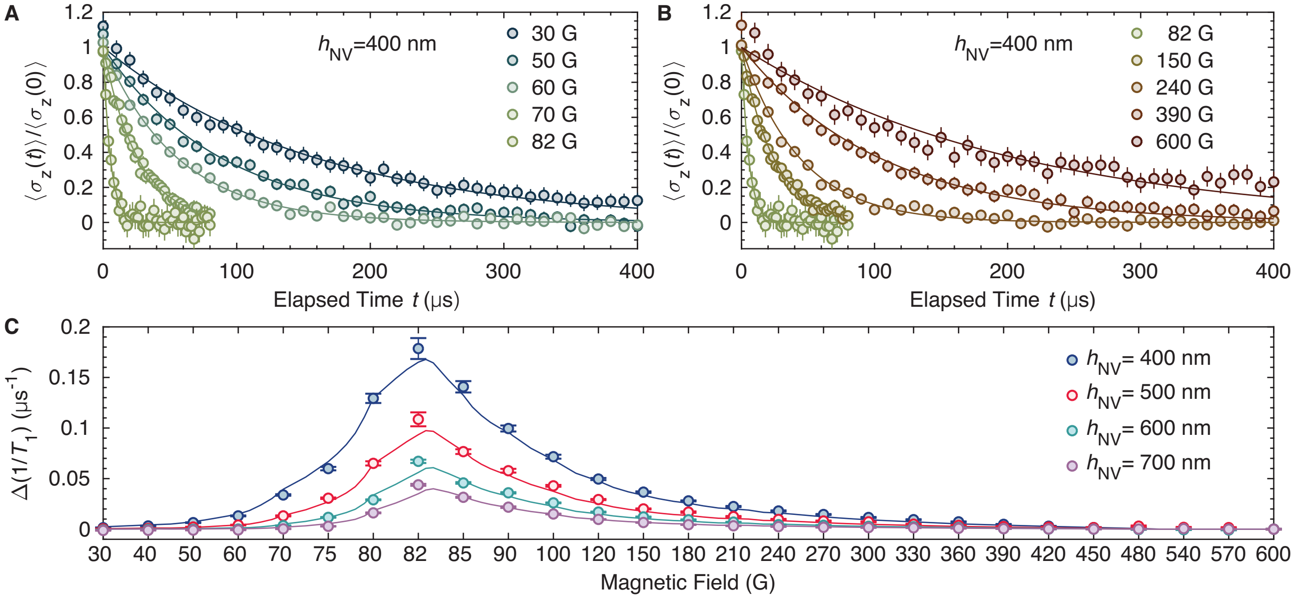

Figure S8: **Time dependence of the differential PL signal.** The differential PL signal which is proportional to $\sigma_z(t)$ at multiple external magnetic fields with (**A**) $\mu_0 H_\parallel \leq 82$ G and (**B**) $\mu_0 H_\parallel \geq 82$ G are shown with different colored markers. The exponential fit is shown with corresponding colors. The vertical axis is normalized such that the prefactor of the exponential fit curve is one. (**C**) Different visualization of Fig. 3A as a reference to (A) and (B), where the horizontal axis is not uniform. The markers are experimental measurements and the solid curves are theoretical calculations with corresponding colors for different h_{NV}. The subtracted offset values are $1/T_1(\mu_0 H_\parallel = 600\ \mathrm{G}) = 0.00485(25)\ \mu\mathrm{s}^{-1}, 0.00422(25)\ \mu\mathrm{s}^{-1}, 0.00414(22)\ \mu\mathrm{s}^{-1}$ and, $0.00421(19)\ \mu\mathrm{s}^{-1}$ for $h_{\mathrm{NV}} = 400\ \mathrm{nm}, 500\ \mathrm{nm}, 600\ \mathrm{nm}$, and $700\ \mathrm{nm}$, respectively.

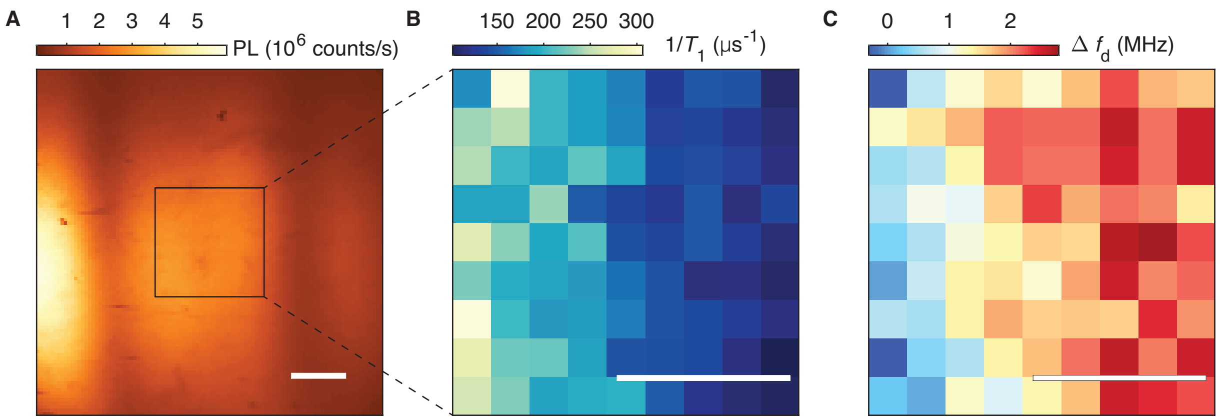

Figure S9: **Spatial image of $1/T_1$ around the area studied in the main text.** (**A**) FSM scan of the PL of an ensemble of the NV centers around the spot of NV centers measured in Fig. 2. The vertical destructive optical interference fringes on the left and right correspond to $h_{\mathrm{diamond-YIG}} = (532/2)$ nm and $h_{\mathrm{diamond-YIG}} = 532$ nm, respectively based on the analysis in Sec. SI.2. Scale bar: 20 μm. (**B**) Spatial scan of the longitudinal relaxation rate $1/T_1$ near $h_{\mathrm{NV}} = 400$ nm in the squared area in (A). The measurement is performed by taking the differential PL signal at two elapsed times $t = 0$ and $t = t_{\mathrm{meas}}$ to speed up the measurement and converted ΔPL into $1/T_1$ following Eq. (S16). The error of the $1/T_1$ measurements is ± 0.019 μs^{-1} on average across the pixels. Scale bar: 20 μm. (**C**) Spatial dependence of the NV-center resonance dip frequency of the ODMR, where $\Delta f_{\mathrm{d}} = f_{\mathrm{dip}} - (D_{\mathrm{NV}} - \gamma\mu_0 H)/2\pi$ is the resonance frequency deviation from the calculated frequency, where f_{dip} is the NV-center frequency determined from the continuous-wave ODMR and D_{NV} is the zero-field splitting of the NV center. Scale bar: 20 μm.

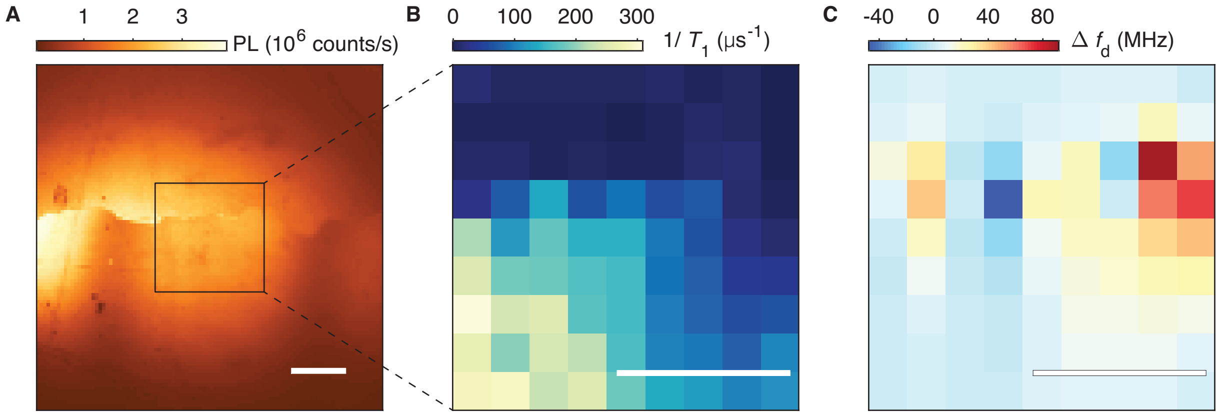

Figure S10: **Spatial image of $1/T_1$ near the YIG edge.** (**A**) FSM scan of the PL of an ensemble of the NV centers near the upper edge of the YIG as shown in Fig. S3A. The vertical destructive optical interference fringes on the left and right within the lower half of the image correspond to $h_{\mathrm{diamond-YIG}} = (532/2)$ nm and $h_{\mathrm{diamond-YIG}} = 532$ nm, respectively, based on the analysis in Sec. SI.2. The horizontal wiggly curve in the middle shows the boundary of the YIG. Scale bar: 20 μm. (**B**) Spatial scan of $1/T_1$ near $h_{\mathrm{NV}} = 400$ nm in the squared area in (A) measured in the same way as in Fig. S9B. The error of $1/T_1$ measurements is ± 0.012 μs^{-1} on average across the pixels. Scale bar: 20 μm. (**C**) Spatial dependence of the resonance dip of the ODM analogously to Fig. S9C. Scale bar: 20 μm.

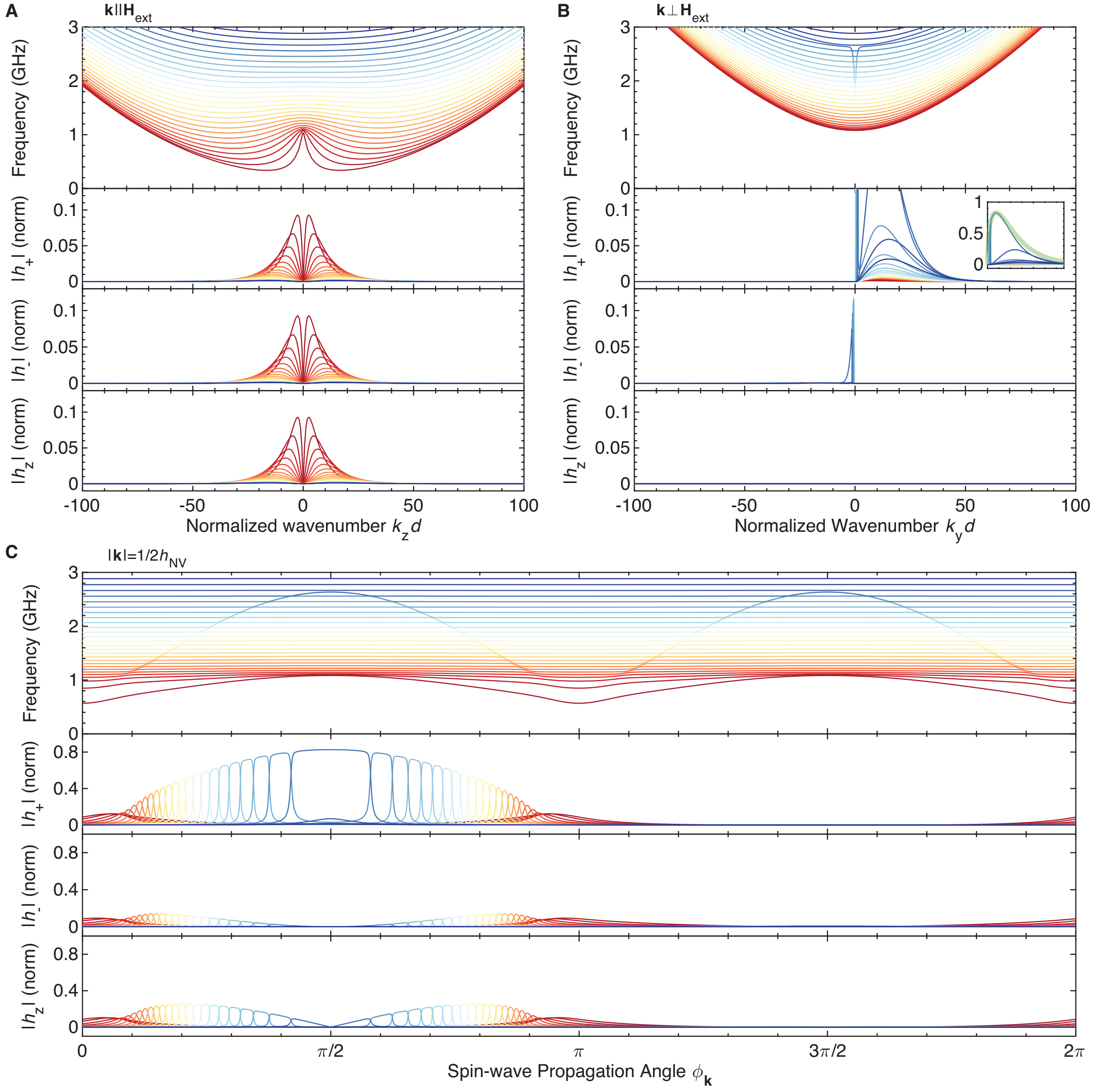

Figure S11: **Dispersion relations of magnons and magnon-originated magnetic fields**. For magnons with the wave vector (**A**) parallel ($\mathbf{k} \parallel \mathbf{H}_{\text{ext}}$) and (**B**) perpendicular ($\mathbf{k} \perp \mathbf{H}_{\text{ext}}$) to the external magnetic field, we show in the top figure the dispersion relation of magnons. The lower three figures are h_+, h_-, and h_z with positive-frequency oscillations [i.e. $\mathbf{h}(t) = \mathbf{h}e^{-i\omega t}$ with the frequency ω shown in the dispersion relation] under a constant spin-wave amplitude [i.e. $\langle \beta_{\mathbf{k},n} \rangle = (\text{constant})$ in Sec. SIII] at the NV center position with a distance $h_{\text{NV}} = 400$ nm from the YIG top surface. Different colors represent different magnon-mode labels n. YIG thickness is $d = 3\ \mu$m and the external magnetic field $H_{\parallel} = 82\ \text{G}/\mu_0$ is applied. Inset of the magnetic field h_+ in (B) is a zoomed-in figure, with the thick green curve representing the dependency obtained from Eq. (S144). (**C**) Magnon propagation angle $\phi_{\mathbf{k}}$ dependence of both the magnon frequency (the top figure) and the magnetic field generated at the NV center position (h_-, h_+, and h_z in the lower figures). Different colors represent different magnon-mode labels n as in (A) and (B). The amplitude of the wave vector is fixed at $|\mathbf{k}| = 1/2h_{\text{NV}}$, which gives the maximum NV-MSSW coupling based on Eq. (S144).

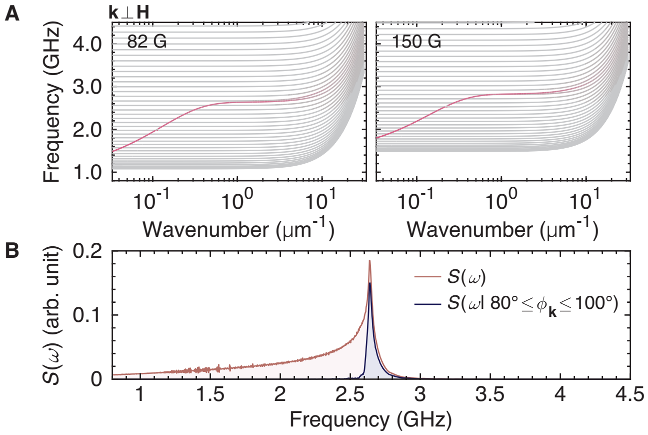

Figure S12: **Dispersion relation of magnons and noise spectrum at the NV center position.** (**A**) More accurate visualization of the magnon dispersion relations with $\mathbf{k} \perp \mathbf{H}$ than in Fig. 2C. Multiple branches represent different thickness-direction magnon-mode labels n. The pink color of the curve shows the surface localization as defined in Eq. (S22). The left and right figures are at 82 G and 150 G, respectively. (**B**) Noise spectrum $\mathcal{S}(\omega)$ (red) and the angle-restricted noise spectrum $\mathcal{S}(\omega|80^\circ \leq \phi_\mathbf{k} \leq 100^\circ)$ (blue) generated by magnons at the NV position as defined in Eqs. (S23) and (S24), respectively. Approximately 81% of the peak height of the full noise spectrum $\mathcal{S}(\omega)$ comes from the contribution of the magnons propagating in an restricted angle range $80^\circ \leq \phi_\mathbf{k} \leq 100^\circ$, which is the reason why we show of the dispersion relation with $\mathbf{k} \perp \mathbf{H}$ in Fig. 2C.

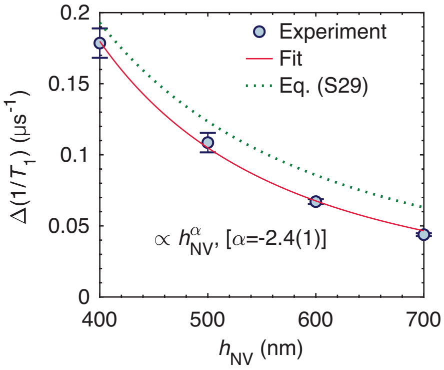

Figure S13: **Power-law fit and analytical expression of the inset of Fig. 3A.** NV-YIG distance h_{NV} dependence of $\Delta(1/T_1)$ at 82 G (the same as the inset of Fig. 3A) with a power fit $\Delta(1/T_1) \propto h_{\mathrm{NV}}^{\alpha}$. The best fit is obtained by $\alpha = -2.4 \pm 0.1$. The dotted green curve shows Eq. (S29).

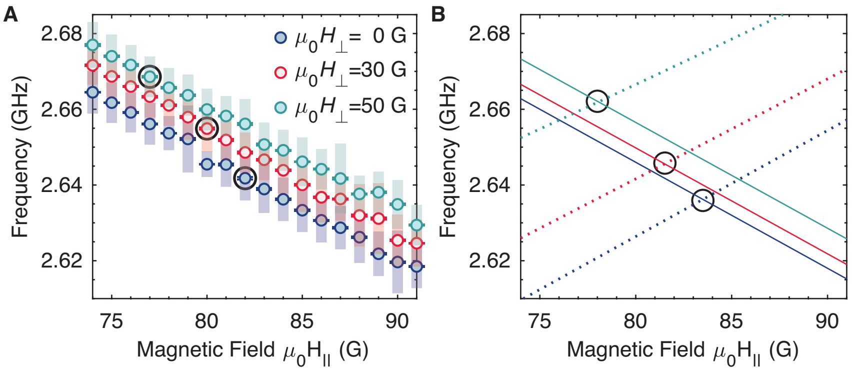

Figure S14: **NV frequency shift as a response to the perpendicular magnetic field.** (**A**) Resonance frequency of NV centers obtained by the continuous-wave ODMR as a function of the external fields $H_{||}$ at multiple orthogonal fields $H_\perp$. The error bars are the fit errors, while the vertical semi-transparent bars are the linewidth of the Lorentzian function of the continuous-wave ODMR, which is shown as another reference to the accuracy of the resonance frequency detection. The field $H_{||}$ at which $\Delta(1/T_1)$ takes maximum is marked with a black circle for each orthogonal field $H_\perp$. (**B**) Calculated NV-center resonance frequency (solid lines) and the MSSW plateau frequency (dotted lines) as a function of the external fields $H_{||}$ at multiple orthogonal fields $H_\perp$ corresponding to (A). The blue, red, and green lines represent orthogonal field of $\mu_0 H_\perp = 0$ G, 30 G, and 50 G, respectively, as in (A). The level crossing conditions are marked with black circles.

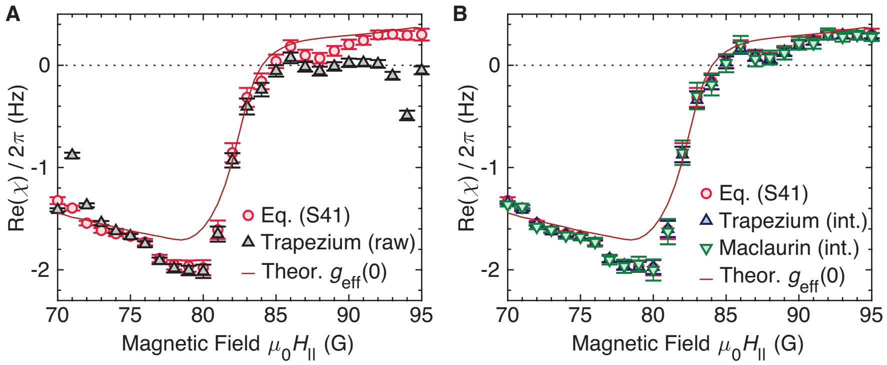

Figure S15: **Kramers-Kronig relation analysis with different procedures.** (**A**) The upper triangular gray markers show the results of the raw trapezium formula without interpolation. The red solid curve and circular markers are the same as in Fig. 4A. (**B**) The blue upper (green lower) triangular markers are based on the trapezium formula (Maclaurin's formula) presented in Ref. (*24*) with interpolation. The red solid curve and circular markers are the same as in Fig. 4A.

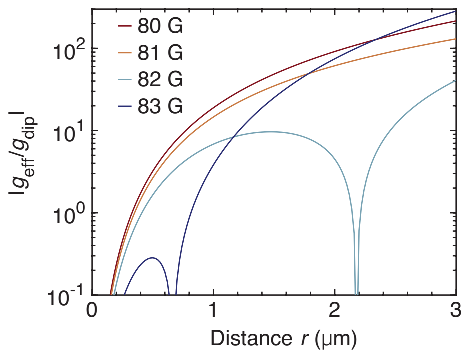

Figure S16: **Ratio of the magnon-mediated coupling $g_{\rm eff}$ to the bare dipole coupling $g_{\rm dip}$ of NV centers.** We show the ratio of the couplings which are individually shown in Fig. 4B. Different colors represent the different external fields.

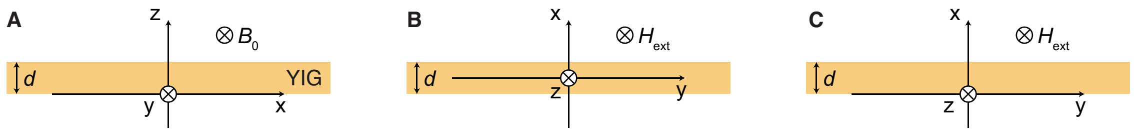

Figure S17: **Different conventions of coordinate systems.** (**A**) Coordinate system used in Ref. (*25*). (**B**) Coordinate system used in Sec. SII. (**C**) Coordinate system used in Sec. SIII.

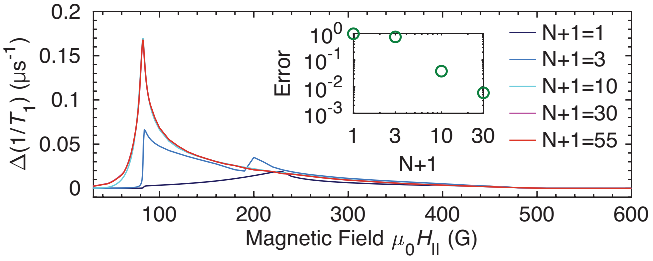

Figure S18: **Convergence of the magnon-induced T_1 decay rate of an NV center in the numerical simulation.** We show the magnetic field dependence of the T_1 decay rate under multiple the number $(N + 1)$ of thickness-direction magnon modes to be taken into account. The YIG thickness is $d = 3\ \mu\mathrm{m}$. The insed shows the error from the case with $N + 1 = 55$, showing that $N + 1 = 55$ is enough for computing the T_1 decay rates.

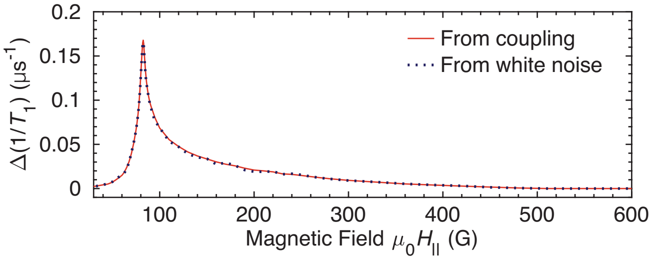

Figure S19: **Comparison between the two approaches of computing the T_1 decay rates of NV centers caused by magnons.** The red solid curve is based on the NV-magnon coupling as described in Sec. SIII.4, while the blue dotted curve is based on the white-noise driven magnons as described in Sec. SIII.5.

References

1. S. Sangtawesin, et al., Origins of diamond surface noise probed by correlating single-spin measurements with surface spectroscopy. *Phys. Rev. X* **9**, 031052 (2019).

2. E. Bauch, et al., Decoherence of ensembles of nitrogen-vacancy centers in diamond. *Phys. Rev. B* **102**, 134210 (2020).

3. K. An, et al., Magnons and phonons optically driven out of local equilibrium in a magnetic insulator. *Phys. Rev. Lett.* **117**, 107202 (2016).

4. G. B. Scott, D. E. Lacklison, J. L. Page, Absorption spectra of $Y_3Fe_5O_{12}$ (YIG) and $Y_3Ga_5O_{12}$: Fe^{3+}. *Phys. Rev. B* **10**, 971–986 (1974).

5. Y. Tateno, M. Fukami, T. Tashiro, K. Ando, Time-resolved spectroscopy of spin-current emission from a magnetic insulator. *Phys. Rev. B* **93**, 174423 (2016).

6. C. S. Wolfe, et al., Off-resonant manipulation of spins in diamond via precessing magnetization of a proximal ferromagnet. *Phys. Rev. B* **89**, 180406 (2014).

7. E. Lee-Wong, et al., Nanoscale detection of magnon excitations with variable wavevectors through a quantum spin sensor. *Nano Letters* **20**, 3284–3290 (2020).

8. P. E. Wigen, *Nonlinear phenomena and chaos in magnetic materials*. (World scientific), (1994).

9. M. Fukami, Y. Tateno, K. Sekiguchi, K. Ando, Wave-vector-dependent spin pumping as a probe of exchange-coupled magnons. *Phys. Rev. B* **93**, 184429 (2016).

10. P. Coleman, *Introduction to many-body physics*. (Cambridge University Press), (2015).

11. A. Rustagi, I. Bertelli, T. van der Sar, P. Upadhyaya, Sensing chiral magnetic noise via quantum impurity relaxometry. *Phys. Rev. B* **102**, 220403 (2020).

12. F. Casola, T. van der Sar, A. Yacoby, Probing condensed matter physics with magnetometry based on nitrogen-vacancy centres in diamond. *Nature Reviews Materials* **3**, 17088 (2018).

13. P.-B. Li, et al., Hybrid quantum device based on NV centers in diamond nanomechanical resonators plus superconducting waveguide cavities. *Phys. Rev. Appl.* **4**, 044003 (2015).

14. M. Fukami, D. R. Candido, D. D. Awschalom, M. E. Flatté, Opportunities for long-range magnon-mediated entanglement of spin qubits via on- and off-resonant coupling. *PRX Quantum* **2**, 040314 (2021).

15. M. K. Bhaskar, et al., Quantum nonlinear optics with a germanium-vacancy color center in a nanoscale diamond waveguide. *Phys. Rev. Lett.* **118**, 223603 (2017).

16. A. Goban, et al., Superradiance for atoms trapped along a photonic crystal waveguide. *Phys. Rev. Lett.* **115**, 063601 (2015).

17. D. E. Chang, A. S. Sørensen, E. A. Demler, M. D. Lukin, A single-photon transistor using nanoscale surface plasmons. *Nature Physics* **3**, 807–812 (2007).

18. E. D. Herbschleb, et al., Ultra-long coherence times amongst room-temperature solid-state spins. *Nature Communications* **10**, 3766 (2019).

19. W. C. Walker, J. Osantowski, Ultraviolet optical properties of diamond. *Phys. Rev.* **134**, A153–A157 (1964).

20. H. R. Phillip, E. A. Taft, Kramers-Kronig analysis of reflectance data for diamond. *Phys. Rev.* **136**, A1445–A1448 (1964).

21. E. Shiles, T. Sasaki, M. Inokuti, D. Y. Smith, Self-consistency and sum-rule tests in the Kramers-Kronig analysis of optical data: Applications to aluminum. *Phys. Rev. B* **22**, 1612–1628 (1980).

22. J. M. Esteban, M. E. Orazem, On the application of the Kramers-Kronig relations to evaluate the consistency of electrochemical impedance data. *Journal of The Electrochemical Society* **138**, 67 (1991).

23. C. A. Emeis, L. J. Oosterhoff, G. D. Vries, Numerical evaluation of Kramers-Kronig relations. *Proceedings of the Royal Society of London. Series A. Mathematical and Physical Sciences* **297**, 54–65 (1967).

24. K. Ohta, H. Ishida, Comparison among several numerical integration methods for Kramers-Kronig transformation. *Applied Spectroscopy* **42**, 952–957 (1988).

25. C. Mühlherr, V. O. Shkolnikov, G. Burkard, Magnetic resonance in defect spins mediated by spin waves. *Phys. Rev. B* **99**, 195413 (2019).

26. V. S. L'vov, *Wave turbulence under parametric excitation: applications to magnets*. (Springer Science & Business Media), (2012).

27. D. D. Stancil, A. Prabhakar, *Spin Waves*. (Springer) Vol. 5, (2009).

28. R. M. Wald, *Quantum field theory in curved spacetime and black hole thermodynamics*. (University of Chicago press), (1994).

29. C. E. Patton, Magnetic excitations in solids. *Physics Reports* **103**, 251–315 (1984).

30. B. A. Kalinikos, A. N. Slavin, Theory of dipole-exchange spin wave spectrum for ferromagnetic films with mixed exchange boundary conditions. *Journal of Physics C: Solid State Physics* **19**, 7013 (1986).

31. B. A. Kalinikos, Excitation of propagating spin waves in ferromagnetic films. *IEE Proceedings H (Microwaves, Optics and Antennas)* **127**, 4–10(6) (1980).

32. B. A. Kalinikos, Spectrum and linear excitation of spin waves in ferromagnetic films. *Soviet Physics Journal* **24**, 718–731 (1981).

33. J. H. P. Colpa, Diagonalization of the quadratic boson hamiltonian. *Physica A: Statistical Mechanics and its Applications* **93**, 327–353 (1978).

34. R. Shindou, R. Matsumoto, S. Murakami, J.-i. Ohe, Topological chiral magnonic edge mode in a magnonic crystal. *Phys. Rev. B* **87**, 174427 (2013).

35. A. G. Gurevich, G. A. Melkov, *Magnetization oscillations and waves*. (CRC press), (1996).

36. H.-P. Breuer, F. Petruccione, , et al., *The theory of open quantum systems*. (Oxford University Press on Demand), (2002).

37. G. S. Agarwal, *Quantum optics*. (Cambridge University Press), (2012).

38. S. Bravyi, D. P. DiVincenzo, D. Loss, Schrieffer-Wolff transformation for quantum many-body systems. *Annals of Physics* **326**, 2793–2826 (2011).

39. J. Rammer, Quantum field theory of non-equilibrium states. *Quantum Field Theory of Non-equilibrium States* (2011).

40. C. Du, et al., Control and local measurement of the spin chemical potential in a magnetic insulator. *Science* **357**, 195–198 (2017).

41. F. Roccati, G. M. Palma, F. Ciccarello, F. Bagarello, Non-hermitian physics and master equations. *Open Systems & Information Dynamics* **29**, 2250004 (2022).

42. D. Manzano, A short introduction to the Lindblad master equation. *AIP Advances* **10** (2020).

43. L. Trifunovic, F. L. Pedrocchi, D. Loss, Long-distance entanglement of spin qubits via ferromagnet. *Phys. Rev. X* **3**, 041023 (2013).
